# Reconfiguring hydrogel assemblies using a photocontrolled metallopolymer adhesive for multiple customized functions

**DOI:** 10.1038/s41557-024-01476-2

**Published:** 2024-03-08

**Authors:** Jiahui Liu, Yun-Shuai Huang, Yazhi Liu, Dachuan Zhang, Kaloian Koynov, Hans-Jürgen Butt, Si Wu

**Affiliations:** 1 Hefei National Research Center for Physical Sciences at the Microscale, CAS Key Laboratory of Soft Matter Chemistry, Anhui Key Laboratory of Optoelectronic Science and Technology, Department of Polymer Science and Engineering, University of Science and Technology of China, Hefei, China; 2 Max Planck Institute for Polymer Research, Mainz, Germany

**Keywords:** Gels and hydrogels, Polymers

## Abstract

Stimuli-responsive hydrogels with programmable shape changes are promising materials for soft robots, four-dimensional printing, biomedical devices and artificial intelligence systems. However, these applications require the fabrication of hydrogels with complex, heterogeneous and reconfigurable structures and customizable functions. Here we report the fabrication of hydrogel assemblies with these features by reversibly gluing hydrogel units using a photocontrolled metallopolymer adhesive. The metallopolymer adhesive firmly attached individual hydrogel units via metal–ligand coordination and polymer chain entanglement. Hydrogel assemblies containing temperature- and pH-responsive hydrogel units showed controllable shape changes and motions in response to these external stimuli. To reconfigure their structures, the hydrogel assemblies were disassembled by irradiating the metallopolymer adhesive with light; the disassembled hydrogel units were then reassembled using the metallopolymer adhesive with heating. The shape change and structure reconfiguration abilities allow us to reprogramme the functions of hydrogel assemblies. The development of reconfigurable hydrogel assemblies using reversible adhesives provides a strategy for designing intelligent materials and soft robots with user-defined functions.

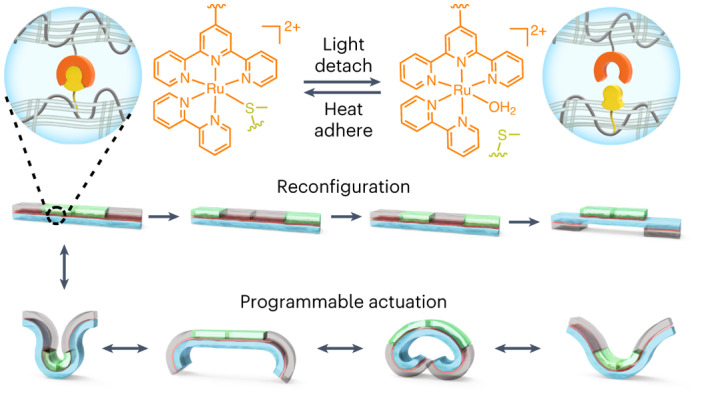

## Abstract (web summary)

Although hydrogels with complex, heterogeneous and reconfigurable structures are promising materials for use in intelligent systems, fabricating such hydrogels is challenging. Now it has been shown that they can be fabricated by reversibly gluing different hydrogel units using a photocontrolled metallopolymer adhesive. This method can be used to design hydrogels with customized functions.

## Main

Science fiction has inspired the development of intelligent materials that can change shape and undergo structure reconfiguration and functional reprogramming in response to external stimuli. Intelligent materials are applicable to soft robots^1–3^, four-dimensional printing^4,5^, biomedical devices^6,7^, sensors^8^ and energy harvesters^9^. Responsive hydrogels are a type of intelligent materials that have been extensively investigated^10–18^. They are active and dynamic networks swollen by water that exhibit volume changes via swelling or shrinkage under the control of pH^15^, temperature^16^, light^12,13,19–21^ and so on^22^. Responsive hydrogels with homogeneous structures undergo only simple expansion/contraction, which limits their applications^19,20^. To endow them with advanced shape change abilities, responsive hydrogels with complex and heterogeneous structures have been fabricated using photolithography^23^, three-dimensional printing^5^ and other techniques^10,24^. These responsive hydrogels displayed various motions, such as bending, winding, rotation, folding and walking^25,26^. To develop intelligent materials using responsive hydrogels, the next challenge is to endow responsive hydrogels with reconfigurable structures and reprogrammable functions. However, the complex and heterogeneous structures in responsive hydrogels are usually linked by covalent bonds, which hinder the reconfiguration of their structures and reprogramming of their functions^5,10^.

A promising approach to address this challenge involves reversibly gluing complex and heterogeneous structures of responsive hydrogels using switchable adhesives. Previous studies have shown that the design of adhesives for wet surfaces of hydrogels is a difficult yet important task^27^, and the development of reversible adhesives for hydrogels is even more challenging. Although some mussel-inspired adhesives and supramolecular adhesives can reversibly glue wet surfaces of hydrogels^28–31^, the use of reversible adhesives to construct reconfigurable hydrogel assemblies is still difficult. This is because ideal reversible adhesives must exhibit strong adhesion to wet surfaces, adapt to shape changes of hydrogels and tolerate external stimuli (for example, pH or temperature) for hydrogel actuation. However, the design of reversible adhesives for hydrogels, which are tolerant to external stimuli and shape changes, poses a challenge. It is desirable to develop advanced adhesives for reversibly gluing intelligent hydrogels.

**Fig. 1:**
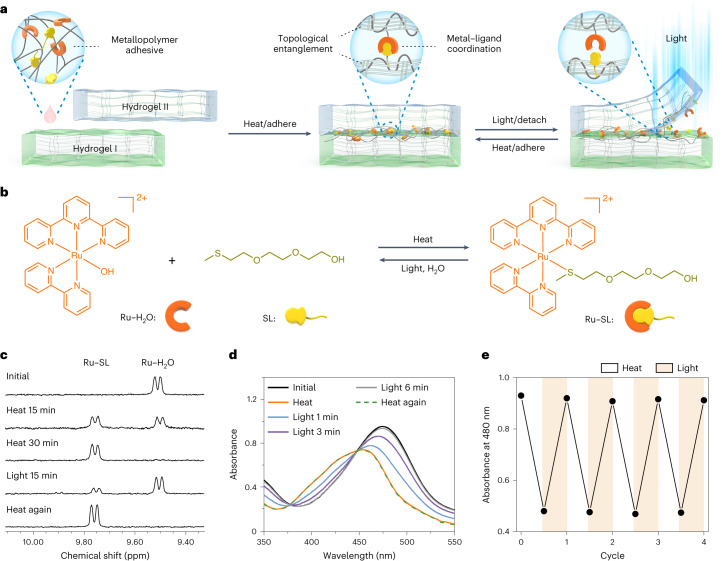
Reversible metal–ligand coordination for photocontrolled metallopolymer adhesives. **a**, Schematic illustration of using the photocontrolled metallopolymer adhesive to reversibly glue hydrogels I and II. **b**, Reversible coordination of the Ru complex (Ru–H_2_O) and the ligand (SL) upon heating and light irradiation. The counterion of Ru–H_2_O is PF_6_^−^. **c**, ^1^H NMR spectra of Ru–H_2_O (4.36 mM) and SL (43.6 mM) in D_2_O before heating, after heating for 15 and 30 min at 70 °C, after light irradiation for 15 min (470 nm, 60 mW cm^−^^2^), and after heating again for 30 min at 70 °C. **d**, UV–vis absorption spectra of Ru–H_2_O (0.1 mM) and SL (1.0 mM) in water before and after heating for 2 h at 70 °C, after light irradiation for 6 min (470 nm, 60 mW cm^−^^2^), and after heating again for 2 h at 70 °C. **e**, Absorption changes of the mixture of Ru–H_2_O and SL in water with alternating heating/light irradiation cycles. Source data

In this Article, we report the fabrication of reconfigurable hydrogel assemblies with reprogrammable functions by reversibly gluing hydrogel units with a metallopolymer adhesive (Fig. 1a,b). The metallopolymer adhesive is a mixture of a Ru-containing polymer and a thioether-containing polymer in water. We designed these polymers because some Ru complexes and (thioether) ligands coordinate and dissociate reversibly via thermal and photochemical reactions, respectively^32–36^. In contrast to the reported reversible adhesives based on mussel-inspired chemistry and supramolecular interactions, reversible adhesives using photocontrolled Ru–ligand coordination have not been investigated. In this work, the metallopolymer adhesive, which contains Ru–S coordination bonds that serve as reversible crosslinks, was used to reversibly glue hydrogel units upon heating and light irradiation (Fig. 1a,b). To reconfigure the glued gel assembly, light irradiation is applied, which induces de-adhesion; the hydrogel units are then separated and can be reused to construct new hydrogel assemblies. The key to designing such hydrogel assemblies is the entanglement/interpenetration of the metallopolymer with the hydrogel. Moreover, the metallopolymer adhesive is a hydrogel that can adapt to the actuation and shape changes of the assembly. Our work demonstrates an approach to fabricate intelligent materials with complex, heterogeneous and reconfigurable structures and reprogrammable functions.

## Results and discussion

### Reversible Ru–S coordination

To demonstrate reversible Ru–S coordination, we synthesized Ru complexes (Ru–H_2_O and Ru–SL) and a thioether ligand (SL) as model compounds (Fig. 1b and Supplementary Figs. 1–19). We studied reversible Ru–S coordination using ^1^H nuclear magnetic resonance (^1^H NMR) spectroscopy (Fig. 1c). When the mixture of Ru–H_2_O (4.36 mM) and SL (43.6 mM) in D_2_O was heated to 70 °C for 30 min, the signal from Ru–H_2_O at 9.50 ppm disappeared, and a new signal from Ru–SL at 9.76 ppm appeared. This result suggests that SL coordinated with the Ru centre. Then, the Ru–SL solution was irradiated with blue light (470 nm, 60 mW cm^−^^2^) for 15 min. The signal at 9.76 ppm notably increased, and the signal at 9.50 ppm greatly decreased, which indicated that most Ru–SL was hydrolysed to form Ru–H_2_O upon light irradiation. The ^1^H NMR data showed that Ru–SL reformed when the sample was heated to 70 °C for 30 min. Thus, Ru–S coordination was reversible upon heating and light irradiation.

We also studied Ru–S coordination using ultraviolet–visible (UV–vis) absorption spectroscopy (Fig. 1d). Initially, the absorption maximum for the mixture of Ru–H_2_O and SL was at 476 nm. This was attributed to the metal-to-ligand charge transfer band of Ru–H_2_O (Supplementary Fig. 10). When the mixture was heated to 70 °C for 2 h, the absorption maximum shifted to 452 nm, which was the same as that of pure Ru–SL (Supplementary Fig. 19). These results showed that SL coordinated with the Ru centre upon heating. Then, the sample was irradiated with blue light (470 nm, 60 mW/cm^2^) for 6 min. The absorption band reverted to the initial state, which suggested that Ru–SL was hydrolysed to form Ru–H_2_O upon light irradiation. Subsequently, the sample was heated again at 70 °C for 2 h, and its absorption maximum reverted to 452 nm. The formation and dissociation of the Ru–S coordination bond was cycled four times (Fig. 1e), which revealed that the Ru–S coordination was reversible. Different from conventional supramolecular interactions or host–guest interactions, the Ru–S coordination did not dissociate upon dilution (Supplementary Fig. 20), which revealed that the Ru–S coordination is a stable yet reversible bond.

### Reversible adhesives for hydrogels

To prepare reversible adhesives based on Ru–S coordination, we synthesized a Ru-containing polymer (P-Ru) and a thioether-containing polymer (P-S) (Fig. 2a and Supplementary Figs. 21–41). P-Ru and P-S were water soluble, and gelation occurred when the mixture of P-Ru (1 wt%) and P-S (1 wt%) in water was heated to 70 °C (Fig. 2b and Supplementary Fig. 45). ^1^H NMR spectroscopy showed that the gelation was attributable to crosslinking via Ru–S coordination (Supplementary Fig. 46). Irradiating the P-Ru/P-S gel with blue light induced a gel-to-sol transition due to light-induced dissociation of Ru–S bonds (Fig. 2b and Supplementary Fig. 46). The sol–gel transitions upon heating and light irradiation were reversible.

**Fig. 2:**
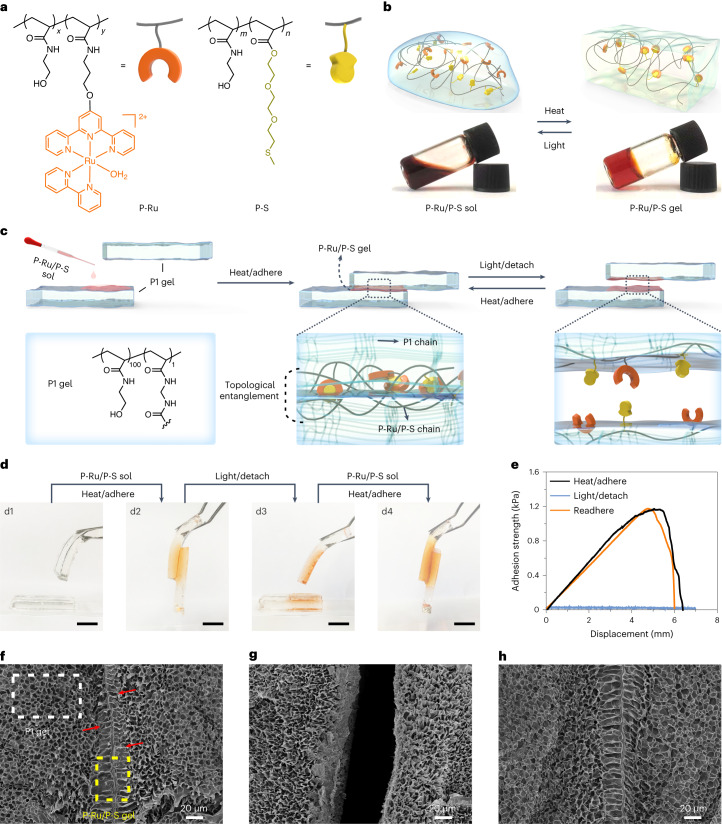
Reversible adhesion of P1 gels based on reversible sol–gel transitions of the P-Ru/P-S adhesive. **a**, Chemical structures of P-Ru (*x*/*y* = 95.3/4.7, number average molecular weight *M*_*n*_ = 16.4 kg mol^−1^ and polydispersity index PDI of 2.91) and P-S (*m*/*n* = 79/21, *M*_*n*_ = 14.9 kg mol^−1^ and PDI of 1.35). The counterion of P-Ru is Cl^−^. **b**, Schematic and photos of reversible sol–gel transitions of P-Ru/P-S on heating and light irradiation. **c**, Schematic illustration of reversibly adhering two P1 gels using the P-Ru/P-S adhesive. **d**, Photos showing reversible adhesion of two P1 gels using the P-Ru/P-S adhesive. Scale bars, 10 mm. **e**, Adhesion strength of adhered P1 gels before irradiation, after light irradiation and after subsequent readhesion. The adhesion measurements were conducted after the samples were immersed in water for 24 h. **f**–**h**, SEM images at the junctions of two adhered P1 gels before irradiation (**f**), after light irradiation (**g**) and after readhesion (**h**). The P1-rich and P-Ru/P-S-rich networks are indicated by the white and yellow frames in **f**, respectively. The interpenetrating networks, which show mixed morphologies of P1 and P-Ru/P-S, are indicated using the red arrows in **f**. Source data

To demonstrate that the P-Ru/P-S mixture can be used as a reversible adhesive, we glued two P1 gels (cross-linked *N*-hydroxyethyl acrylamide) together (Fig. 2c). To glue the P1 gels, P-Ru/P-S sol (6 wt%) was placed between the gels and heated to 70 °C. Heating induced the sol-to-gel transition (Fig. 2d). To separate the glued P1 gels, light irradiation was applied to induce a gel-to-sol transition. The separated P1 gels were readhered by adding P-Ru/P-S sol and heating. Control experiments showed that P1 gels could not be glued using P-Ru or P-S alone (Supplementary Fig. 47), which demonstrated that the sol–gel transition of P-Ru/P-S is essential for adhesion.

We quantified the adhesion of P-Ru/P-S adhesives to P1 gels via lap shear tests (Fig. 2e and Supplementary Figs. 48–51). The adhesion strength of P1 gels glued by P-Ru/P-S (6 wt%) was 1.18 kPa. Light irradiation induced a gel-to-sol transition, and the adhesion strength decreased to almost zero. Thus, P1 gels could be separated after light irradiation. Subsequently, the separated P1 gels were readhered using P-Ru/P-S upon heating. The readhered sample had almost the same adhesion strength as the initial sample.

To understand why the P-Ru/P-S adhesive exhibited strong yet reversible adhesion to P1 gels, the morphologies at the junctions of two P1 gels glued by a P-Ru/P-S adhesive were studied using scanning electron microscopy (SEM) (Fig. 2f–h). Both P1 and P-Ru/P-S formed porous gel networks (Supplementary Fig. 52). The average pore diameter of the P-Ru/P-S gel was approximately three times larger than that of the P1 gel. For the adhered and readhered samples, some network structures with both small and large pores overlapped at the junctions of the P1 and P-Ru/P-S gels (Fig. 2f,h), which indicated that the networks of P1 and P-Ru/P-S interpenetrated with each other. For the sample after light irradiation, the P-Ru/P-S network disappeared (Fig. 2g), which resulted in de-adhesion.

**Fig. 3:**
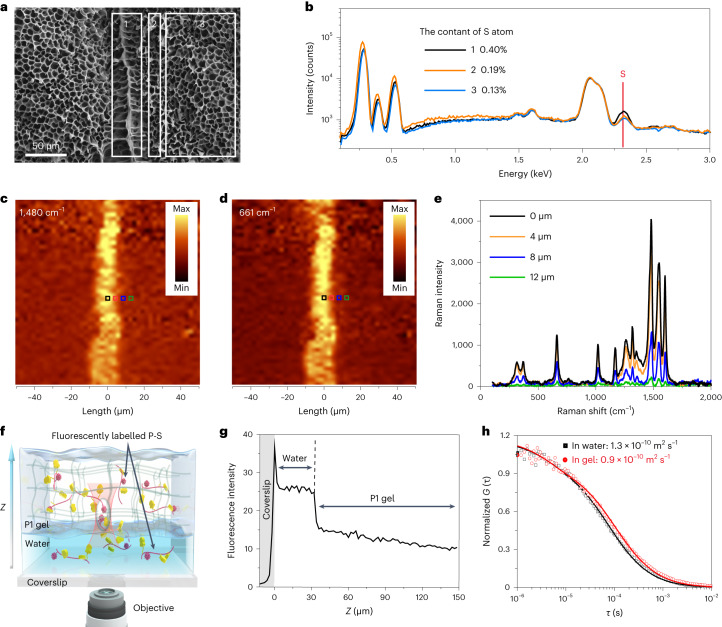
Characterization of the interpenetrating network and the mechanism for its formation. **a**, SEM image at the junctions of two P1 gels glued by a P-Ru/P-S adhesive. The P-Ru/P-S-rich region, junctional region and P1-rich region are labelled with 1, 2 and 3, respectively. **b**, EDS data of regions 1, 2 and 3. The contents of S atoms in these regions are 0.40%, 0.19% and 0.13%, respectively. **c**–**e**, Raman mapping at the junctions of two P1 gels glued by a P-Ru/P-S adhesive based on the Raman signals at 1,480 cm^−^^1^ (**c**) and 661 cm^−^^1^ (**d**) and Raman spectra at different areas in the Raman maps (**e**). The areas highlighted by coloured boxes in **c** and **d** correspond to the signals in **e**. **f**, Scheme for the FCS experiment. The distribution of the fluorescently labelled P-S along the *Z* direction was scanned by adjusting the distance between the sample and the objective. **g**, Fluorescence intensity along the *Z* direction. **h**, Normalized fluorescence intensity autocorrelation functions *G*(*τ*) recorded for the fluorescently labelled P-S in a P1 gel (red circles) and in the water phase (black squares). The solid lines represent the fittings with Supplementary Equation 1. Source data

To characterize the structures at the junctions of P1 and P-Ru/P-S, energy-dispersive spectroscopy (EDS) of SEM was used. The signals of S in P-S and Ru in P-Ru were measured to show the distributions of P-S and P-Ru. The EDS data showed that the contents of S in a P-Ru/P-S-rich region (region 1), junctional region (region 2) and P1-rich region (region 3) were 0.40%, 0.19% and 0.13%, respectively (Fig. 3a,b). These data showed that P-S penetrated into P1. EDS also detected Ru in these three regions (Supplementary Fig. 53). However, the contents of Ru measured by EDS were lower than 0.1%, which is the lower limit for quantitative analysis. The contents of Ru were lower than those of S because the ratio of Ru/S in P-Ru/P-S was 4.7/21. To further investigate the distribution of P-Ru, we used the Raman mapping technique, which is described below.

P-Ru/P-S showed strong Raman signals. The C–C and C=N stretching vibrations of the polypyridine skeleton of P-Ru were at 1,480, 1,544 and 1,600 cm^−^^1^, and the C–S–C stretching vibration of P-S was at 661 cm^−^^1^ (Supplementary Fig. 54). The P1 gel did not have any characteristic peak in the Raman spectrum (Supplementary Fig. 55). Raman maps at the junctions of two P1 gels glued by a P-Ru/P-S adhesive were obtained by scanning a 100 µm^2^ × 100 µm^2^ region using a focused laser beam with a diameter of ~2 µm (Fig. 3c–e and Supplementary Fig. 56). Although the Raman signals of P-Ru/P-S decreased as the laser focus moved from the P-Ru/P-S-rich region to the P1-rich region, P-Ru/P-S was detected in the P1-rich region. These results showed that P-Ru/P-S interpenetrated with P1.

To understand the mechanism for the formation of the interpenetration network, we synthesized fluorescently labelled P-S (Supplementary Figs. 42–44) and studied its diffusion using fluorescence correlation spectroscopy (FCS). A P1 gel was placed in a sample cell that contained an aqueous solution of fluorescently labelled P-S (10 nM). A layer of aqueous solution remained between the P1 gel and the coverslip at the bottom of the sample cell. The sample cell was placed on top of the objective of the inverted confocal microscope (Fig. 3f). The fluorescence intensity along the perpendicular direction was scanned (Fig. 3g). The appearance of fluorescence in the P1 gel suggested that some fluorescently labelled P-S diffused from the water phase into the P1 gel. Next, FCS autocorrelation curves were recorded in both the water phase and the P1 gel (Fig. 3h) and fitted using Supplementary Equation 1. The diffusion coefficients of the fluorescently labelled P-S in water and the P1 gel were 1.3 × 10^−^^10^ and 0.9 × 10^−^^10^ m^2^ s^−1^, respectively. The P1 gel caused a 31% slowdown of the diffusion, which indicated that the network of the P1 gel hindered the diffusion of the fluorescently labelled P-S. Because the decrease of the diffusion coefficient was not large, the fluorescently labelled P-S can still penetrate into the P1 gel.

To provide molecular-level insight into the interpenetration, we performed computer simulations (Supplementary Figs. 57–61). Initially, P-Ru and P-S out of the P1 gel diffused freely. After diffusion for a few nanoseconds, P-S and P-Ru came into contact with P1, and hydrogen bonds between P1 and P-Ru/P-S were formed. Although steric hindrance and hydrogen bonding slowed their diffusion, P-S and P-Ru still penetrated into the P1 gel because P1 is porous and hydrogen bonds are dynamic, which could break and reconfigure. The computer simulation revealed that P-Ru and P-S penetrated into the P1 gel via free diffusion and diffusion under steric hindrance and hydrogen bonding reconfiguration.

To study the effects of interpenetration on adhesion, we performed control experiments by replacing P1 gels with polyethylene and Teflon substrates (Supplementary Fig. 62). Because polyethylene and Teflon substrates are hydrophobic and water insoluble, the aqueous solutions of the adhesive cannot penetrate into them. The adhesion energy of P-Ru/P-S-glued P1 gels is more than 375% of that of P-Ru/P-S-glued polyethylene or Teflon substrates, which suggested that the interpenetration of P-Ru/P-S with P1 enhanced the adhesion.

### Hydrogel actuators assembled by the reversible adhesive

Importantly, the P-Ru/P-S adhesive is tolerant to temperature and pH, both of which are frequently used stimuli for hydrogel actuation. After the P1 gels, glued with a P-Ru/P-S adhesive, were treated at different temperatures (25 °C and 70 °C) and pH values (4, 7 and 10) for 24 h, the adhesion strength did not change (Supplementary Fig. 63). This environmental tolerance enabled the fabrication of responsive hydrogel actuators using the P-Ru/P-S adhesive, as described below.

**Fig. 4:**
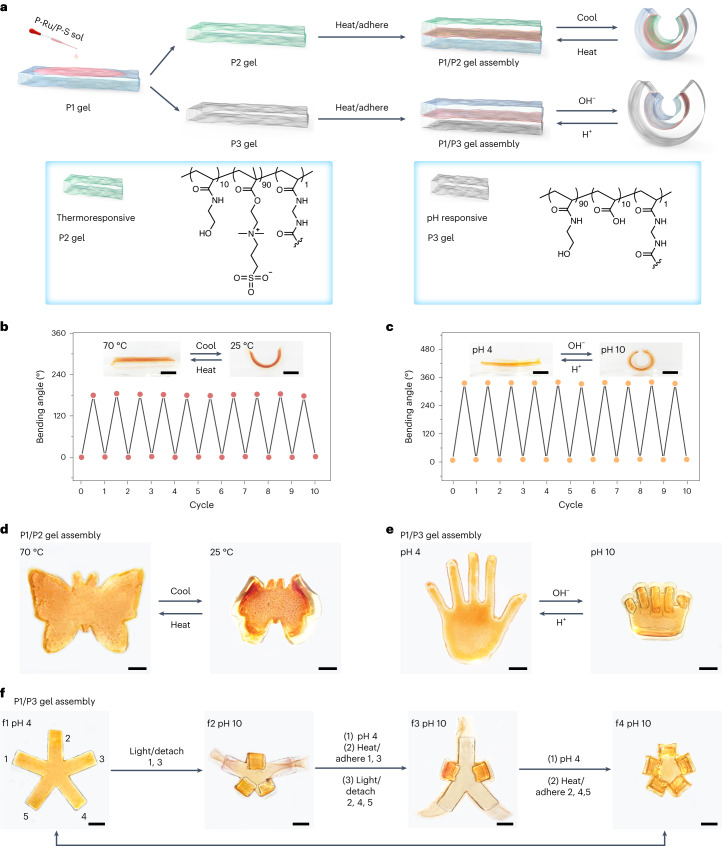
Preparation and actuation of responsive hydrogel assemblies. **a**, Schematic showing preparation of responsive hydrogel assemblies using P-Ru/P-S as an adhesive and shape changes of the hydrogel assemblies actuated by temperature or pH. **b**, Reversible bending of a P1/P2 gel assembly at different temperatures. The bending angles were measured after immersing the gel in water at 25 °C or 70 °C for 1 h. **c**, Reversible bending of a P1/P3 gel assembly at different pH values. The bending angles were measured after immersing the gel in an aqueous solution with pH 4 or 10 for 30 min. **d**, Photos of a butterfly-shaped P1/P2 gel assembly at different temperatures. **e**, Photos of a hand-shaped P1/P3 gel assembly at different pH values. **f**, Reversible detachment and adhesion of some arms of a five-arm P1/P3 gel assembly and shape changes of the gel assembly at different pH values. Scale bars, 5 mm. Source data

To prepare responsive hydrogel actuators, we used the P1 gel, thermoresponsive P2 gel, and pH-responsive P3 gel as units (Fig. 4a). P1/P2 gel assemblies and P1/P3 gel assemblies were fabricated by gluing the corresponding gels using the P-Ru/P-S adhesive. The P1 gel was inert to temperature or pH change. The P2 gel swelled and shrank upon heating to 70 °C and cooling to 25 °C because of the hydration and dehydration of the zwitterionic moiety (Supplementary Fig. 64). Therefore, the P1/P2 gel assembly reversibly bent and unbent upon cooling and heating (Fig. 4b). We prepared a butterfly-shaped P1/P2 assembly, which beat its wings via temperature actuation (Fig. 4d).

We also prepared pH-actuated P1/P3 gel assemblies (Fig. 4c,e). The P3 gel swelled at pH 10 and shrank at pH 4 because of the deprotonation and protonation of the acrylic acid moiety (Supplementary Fig. 65). Thus, pH changes induced bending/unbending of a rectangular P1/P3 gel assembly and triggered opening/closing of a hand-shaped P1/P3 gel assembly (Fig. 4c,e). Bending and unbending were fully reversible for at least ten cycles (Fig. 4c). This observation demonstrated that the P-Ru/P-S adhesive glued pH-responsive hydrogel was firmly assembled, even under large shape changes. Lap shear measurements showed that the P1/P2 gel assembly and P1/P3 gel assembly were glued firmly even after ten bending/unbending cycles (Supplementary Figs. 66 and 67). We interpret that the strong adhesion is attributed to the following reasons: (1) Ru–S coordination and polymer chain entanglement cooperate (Fig. 3); (2) the adhesion of P-Ru/P-S is independent of pH and temperature (Supplementary Fig. 63); and (3) the P-Ru/P-S adhesive is a hydrogel that can change shape and maintain its integrated network structure during swelling and shrinkage (Supplementary Fig. 68). Thus, the P-Ru/P-S adhesive can adapt to the shape changes of the gel assemblies.

To verify that the P-Ru/P-S adhesive is strong yet reversible, we prepared a five-arm P1/P3 gel assembly using the adhesive (Fig. 4f). The gel assembly showed reversible shape changes when varying the pH (Fig. 4f, f1 and f4). To illustrate the advantage of light and its high spatial resolution, some of the adhered arms were selectively detached via light irradiation (Figs. 4f, f2 and f3), and the gel assembly was changed to different shapes via pH activation. The detached arms could be readhered to form the initial five-arm structure (Fig. 4f, f4).

### Reconfigurable hydrogel assemblies with customized shapes

**Fig. 5:**
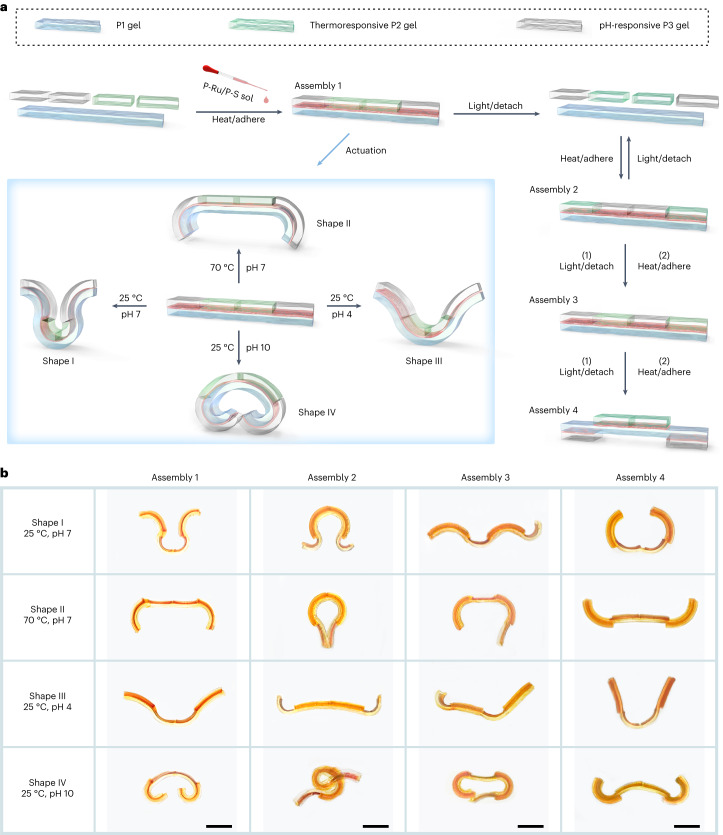
Reconfiguring the structures of responsive hydrogel assemblies for multiple customized actuation. **a**, Schematic showing fabrication and reconfiguration of hydrogel assemblies with complex and heterogeneous structures and shape changes at different temperatures and pH values. Five hydrogel units can be glued using the P-Ru/P-S adhesive to form assembly 1. Assembly 1 can be reconfigured to assemblies 2–4. Each assembly can be changed to four shapes. **b**, Photos of hydrogel assemblies at different temperatures and pH values. Each of the four assemblies underwent a different shape change under each of the four conditions tested. Scale bars, 10 mm.

The reversible P-Ru/P-S adhesive enables the fabrication of reconfigurable hydrogel assemblies with reprogrammable shape changes. We prepared assembly 1 with complex and heterogeneous structures by gluing a P1 gel, two P2 gels and two P3 gels together (Fig. 5a). Assembly 1 changed into four shapes at different temperatures and pH values (Fig. 5b, first column). Moreover, the structure of assembly 1 could be reconfigured by light-induced detachment and readhesion using the P-Ru/P-S adhesive. Therefore, we prepared assemblies 2, 3 and 4 via reconfiguration using the same hydrogel units. Each gel assembly changed into four shapes upon pH and temperature stimulation (Fig. 5b). These hydrogel assemblies are intelligent multishape transformers. The results showed that the use of the reversible metallopolymer adhesive is a strategy for the preparation of complex, heterogeneous and reconfigurable hydrogel assemblies with reprogrammable actuation functions.

### Soft robot based on hydrogel assemblies for maze navigation

**Fig. 6:**
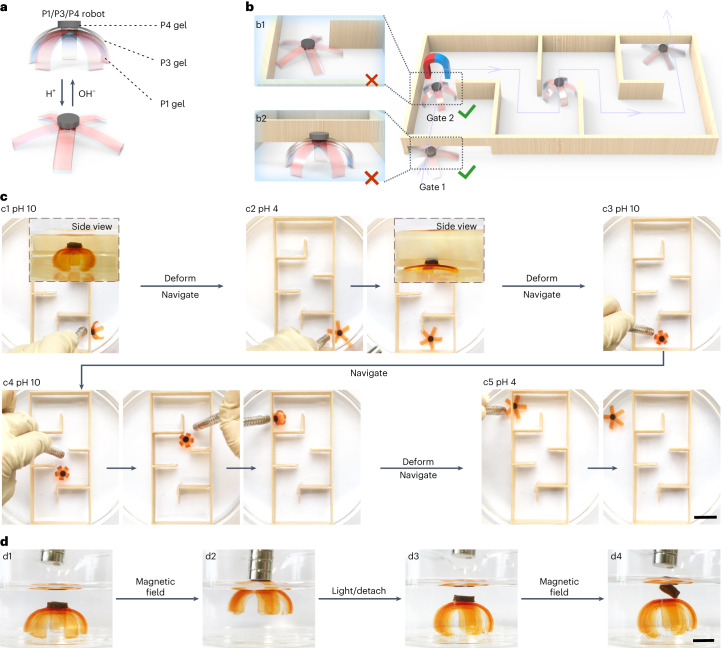
Soft robot based on a responsive gel assembly for maze navigation. **a**, Schematic of a P1/P3/P4 robot prepared by gluing the gel units using the P-Ru/P-S adhesive. P1 is a non-responsive gel, P3 is a pH-responsive gel and P4 is a magnetic particle-containing gel. **b**,**c**, Schematic illustration (**b**) and photos (**c**) of the P1/P3/P4 robot passing through a maze under the control of pH and a magnetic field. The inset of c1 shows that the robot was too tall to pass through the gate. The inset of c2 shows that the flattened robot passed the gate. Scale bar: 20 mm. **d**, P1/P3/P4 robot under the action of a magnetic field (d1 and d2). After light irradiation, the P1/P3 gels and P4 gel were separated (d3 and d4). Scale bar, 5 mm.

We fabricated a soft robot with complex and heterogeneous structures by gluing a non-responsive P1 gel, a pH-responsive P3 gel and a magnetic particle-containing P4 gel using the P-Ru/P-S adhesive (Fig. 6a). The P4 gel contained magnetic Ni particles so that it could move in a magnetic field. The P1/P3/P4 robot was firmly glued using the P-Ru/P-S adhesive. Thus, the P1 and P3 units followed the movement of the P4 unit in a magnetic field (Supplementary Movie 1).

The P1/P3/P4 robot could go through a maze under the stimulation of pH and a magnetic field (Supplementary Movie 2). At pH 10, the robot was too tall to pass through Gate 1 (Fig. 6b,c, b2 and c1). When the pH was adjusted to 4, the robot became flat and passed through gate 1 under the guidance of a magnetic field (Figs. 6b,c, b2 and c2). However, the flattened robot could not pass through Gate 2 (Fig. 6b, b1). Therefore, the robot was reshaped by changing the pH to 10, and it passed through gate 2 under the guidance of a magnetic field (Figs. 6b,c, b1 and c3). In similar ways, the robot went through the additional gates and tortuous path of the maze (Fig. 6c, c4 and c5).

To demonstrate that the robot is reconfigurable, the P1/P3 gels and P4 gel were separated via light irradiation (Fig. 6d and Supplementary Movie 3). The P1/P3 gels were reused as an actuator, which is the same as the actuator demonstrated in Fig. 4f. The P4 gel was reused by gluing it to a five-arm P1/P2 assembly to prepare a P1/P2/P4 robot (Supplementary Fig. 69 and Supplementary Movie 4). Because P2 was thermoresponsive and P4 was a magnetic particle-containing gel, the P1/P2/P4 robot passed through a maze under the control of temperature and a magnetic field (Supplementary Fig. 69 and Supplementary Movie 5).

## Conclusion

In conclusion, we have demonstrated the fabrication of complex, heterogeneous, multiresponsive hydrogel assemblies with reconfigurable structures and reprogrammable functions by reversibly gluing hydrogel units using a metallopolymer adhesive. The newly designed metallopolymer adhesive adheres firmly and reversibly to the wet surfaces of hydrogels. It adapts to the actuation and shape changes of hydrogels. It is also tolerant to external stimuli (for example, pH or temperature) for hydrogel actuation. These features are unique. The combination of photocontrolled Ru–S coordination and polymer chain entanglement is distinct from the mussel-inspired chemistry and supramolecular chemistry for adhesives reported in the literature and represents a strategy for designing strong yet reversible adhesives. We anticipate that, similar to the actuators and soft robots reported here, other intelligent materials with multiple components, reconfigurability, programmability and customizable functions can be fabricated by reversibly gluing intelligent building blocks. Our study opens up an avenue for the design of responsive materials, four-dimensional printing materials, biomaterials and soft robots.

## Methods

### Synthesis

The synthesis and characterization of Ru complexes, thioethers, P-Ru, P-S and fluorescently labelled P-S are provided in Supplementary Information (Supplementary Figs. 1–44).

### Preparation of hydrogels as adherends

#### Non-responsive P1 gel

*N*-hydroxyethyl acrylamide (HEA) (690 mg, 5.9 mmol) was dissolved in 6 ml de-ionized water. *N*,*N*′-methylenebisacrylamide (Bis) (9 mg, 0.06 mmol) and Irgacure 2959 (12 mg, 0.05 mmol) were added to the HEA solution. The precursor solution was de-gassed and filled with argon for 20 min. After that, the solution was poured into a Teflon mold, covered with a glass plate and exposed to UV light (8 W, 366 nm curing light, CAMAG) for 2 h. The obtained gel was immersed in water to reach swelling equilibrium.

#### Thermoresponsive P2 gel

3-Dimethyl (methacryloyloxyethyl) ammonium propane sulfonate (1,508 mg, 5.39 mmol) and HEA (69 mg, 0.60 mmol) were dissolved in 6 ml de-ionized water. Bis (9 mg, 0.06 mmol) and Irgacure 2959 (12 mg, 0.05 mmol) were sequentially added to the mixture. The mixture was de-gassed and filled with argon for 20 min. After that, the mixture was poured into a Teflon mold, covered with a glass plate and exposed to UV light (8 W, 366 nm curing light, CAMAG) for 2 h. The obtained gel was immersed in water to reach swelling equilibrium.

#### pH-responsive P3 gel

HEA (621 mg, 5.3 mmol) and acrylic acid (39 mg, 0.54 mmol) were dissolved in 6 ml de-ionized water. Bis (9 mg, 0.06 mmol) and Irgacure 2959 (24 mg, 0.10 mmol) were added to the mixture. The mixture was de-gassed and filled with argon for 20 min. After that, the mixture was poured into a Teflon mold, covered with a glass plate and exposed to UV light (8 W, 366 nm curing light, CAMAG) for 2 h. The obtained gel was immersed in water to reach swelling equilibrium.

#### Magnetic particle-containing P4 gel

HEA (230.26 mg, 2 mmol) was dissolved in 1 ml of de-ionized water. Bis (0.33 mg, 0.0002 mmol), MagneHis Ni-Particle suspension (0.1 ml) and VA-044 (5 mg, 0.015 mmol) were added to the HEA solution. The solution was de-gassed and filled with argon for 20 min. After that, the solution was poured into a Teflon mold, sealed with a glass plate and placed in an oven at 50 °C for 12 h. The obtained gel was immersed in water to reach swelling equilibrium.

### Preparation of hydrogel assemblies using P-Ru/P-S as an adhesive

An aqueous solution of P-Ru/P-S (6 wt%, 10 μl cm^−^^2^) was spread on the surfaces of the hydrogels. Then, one hydrogel was pressed on top of another hydrogel. The hydrogels were sealed and placed in a container at 70 °C for 6 h. After that, a gel assembly was obtained and the gel assembly was immersed in water before further investigation.

## Online content

Any methods, additional references, Nature Portfolio reporting summaries, source data, extended data, supplementary information, acknowledgements, peer review information; details of author contributions and competing interests; and statements of data and code availability are available at 10.1038/s41557-024-01476-2.

### Supplementary information

Supplementary Information Supplementary Figs. 1–69, Tables 1–2 and discussion.

Supplementary Data 1 Source data for supplementary figures.

Supplementary Data 2 Computational data.

Supplementary Video 1 Magnetic and pH-responsive hydrogel robot.

Supplementary Video 2 Maze navigation by magnetic and pH-responsive hydrogel robot.

Supplementary Video 3 Magnetic and pH-responsive hydrogel robot after light irradiation.

Supplementary Video 4 Magnetic and thermoresponsive hydrogel robot.

Supplementary Video 5 Maze navigation by magnetic and thermoresponsive hydrogel robot.

### Source data

Source Data Fig. 1 ^1^H NMR and UV–vis spectra of Ru–H_2_O and SL in D_2_O before heating, after heating, after light irradiation and after heating again. Absorption changes of the mixture of Ru–H_2_O and SL in water with alternating heating/light irradiation cycles.

Source Data Fig. 2 Adhesion strength of adhered P1 gels before irradiation, after light irradiation and after subsequent readhesion.

Source Data Fig. 3 EDS data, Raman data, fluorescence intensity along the *Z* direction and normalized fluorescence intensity autocorrelation function.

Source Data Fig. 4 The bending angles of P1/P2 gel assembly at different temperatures, and the bending angles of P1/P3 gel assembly at different pH.

## Data Availability

All data generated or analysed during this study are included in this published article and its supplementary information files. Source data are provided with this paper.
