# Supplementary material for: Reconfiguring hydrogel assemblies using a photocontrolled metallopolymer adhesive for multiple customized functions
Source: Nat Chem. 2024 Mar 8;16(6):1024–33. doi: 10.1038/s41557-024-01476-2 (PMC11164683; doi:10.1038/s41557-024-01476-2)
Supplement: Supplementary file 1 — Supplementary Figs. 1–69, Tables 1–2 and discussion. [file 41557_2024_1476_MOESM1_ESM.pdf]

nature chemistry

Article https://doi.org/10.1038/s41557-024-01476-2

Reconfiguring hydrogel assemblies using a photocontrolled metallopolymer adhesive for multiple customized functions

In the format provided by the authors and unedited

Table of Contents

Materials.. 2

Instruments and Characterization.. 2

Synthesis.. 4

Preparation of P-Ru/P-S hydrogels.. 34

Photocontrolled reversible coordination of P-Ru and P-S.. 35

Control experiments for adhesion of hydrogels.. 37

Measurements of adhesion strength of glued P1 gels.. 37

SEM images of P-Ru/P-S gel and P1 gel.. 42

EDS results of P-Ru/P-S-glued P1 gel.. 43

Raman spectra and imaging.. 43

Computer simulations.. 45

Adhesion strength of different substrates glued by P-Ru/P-S adhesives.. 51

Adhesion strength of P-Ru/P-S-glued P1 gels in different aqueous environments.. 52

Volume changes of P2 and P3 gels under different stimuli.. 52

Measurements of adhesion strength of P1/P2 and P1/P3 gel assemblies.. 53

Shape changes of a P-Ru/P-S gel and swelling of a P-Ru/P-S gel in water.. 55

Soft robot based on a responsive hydrogel assembly for maze navigation.. 56

References.. 57

Materials

$RuCl_3$•xH_2O (99.9%) was purchased from Fisher Scientific. Acryloyl chloride (97.0%), 3-amino-1-propanol (99%), N,N'-methylenebisacrylamide (Bis) (>99.5%), magnesium sulfate (>98%), sodium chloride (>99%), sodium thiomethoxide (95%) and Irgacure 2959 (98%) were purchased from Sigma-Aldrich. 2,2'-Bipyridine (bpy) (>99%), silver hexafluorophosphate (>98%), potassium hexafluorophosphate (>98%), 2-[2-(2-chloroethoxy)ethoxyl] ethanol (>96%), N-hydroxyethyl acrylamide (HEA) (>98%), 2,2'-azobis[2-(2-imidazolin-2-yl) propane]dihydrochloride (VA-044) (>98%), acrylic acid (AAc) (>99%) and 3-dimethyl (methacryloyloxyethyl) ammonium propane sulfonate (DMAPS) (95%) were purchased from TCI. 2,2':6',2''-Terpyridine (tpy) (97%) was purchased from Alfa Aesar. 4'-Chloro-2,2',6',2"-terpyridine (>98%) was purchased from AEchem Scientific Corporation. MagneHis™ Ni-Particle was purchased from Promega. N-(2-Aminoethyl)acrylamide hydrochloride was purchased from Aldrich Partner. Atto514 N-hydroxysuccinimidyl (NHS)-ester was purchased from ATTO-ETC GmbH. All solvents (HPLC grade) were purchased from Sigma-Aldrich or Fisher Scientific.

Instruments and Characterization

^1H nuclear magnetic resonance (^1H NMR) and ^{13}C nuclear magnetic resonance (^{13}C NMR) spectra were recorded on a 300-MHz Bruker Spectrospin NMR spectrometer. The molecular weights were determined using mass spectrometry (Bruker Time-of-flight MS Reflex III and Expression L Compact Mass Spectrometer). UV-vis absorption spectra were measured using a Lambda 900 spectrometer (Perkin Elmer). The molecular weights and polydispersity indexes of the polymers were determined using a PSS-Win GPC (pump: SECcurity) equipped with UV and RI detectors running in dimethylformamide (DMF) with 1 g/L LiBr at 60 °C calibrated against polystyrene standards. Lap shear tests were performed using a Zwick/Roell system with a deformation rate of 20 mm min^{-1}. To fix the gel assemblies between the clamps of the tensile

machine, a polyester film (thickness: 0.35 mm) was used as a back layer glued on the gels using a commercial superglue. The morphologies of the gels and energy dispersive spectroscopy (EDS) were observed using scanning electron microscopy (SEM, HITACHI). Raman spectra and Raman mapping were recorded using a confocal Raman imaging system (WITEC Alpha 300 R), which was equipped with a laser at 532 nm and a 50× objective lens. The diameter of the laser at the focus was ~2 μm.

Fluorescence correlation spectroscopy (FCS) experiments were performed on an LSM 880 confocal microscope system (Carl Zeiss, Germany). The samples were excited using an argon laser at 488 nm, which was focused through a C-Apochromat 40×/1.2 W water immersion objective (Carl Zeiss, Germany). The emission was collected with the same objective. After the emitted light passed through a confocal pinhole, it was directed to a spectral detector (Quasar, Carl Zeiss) to detect the light from 500 to 600 nm. An Attofluor™ stainless steel chamber with a mounted glass coverslip bottom was used as a sample cell for this study. We placed a P1 gel in a sample cell containing an aqueous solution of fluorescently labeled P-S (10 nM). The sample cell with a glass coverslip at the bottom was placed on top of the FCS setup. A thin spacer was placed between the bottom of the sample cell and the P1 gel to ensure that a water layer was present below the P1 gel. Fluorescence intensity along the Z direction was recorded to identify the locations of the P1 gel and the fluorescently labeled P-S. Then, the confocal volume was positioned either in the P1 gel or in the water phase below it. A series of 10 FCS measurements with a total duration of 5 min were performed at each position. The time-dependent fluctuations of the fluorescent intensity $\delta I(t) = I(t) - \langle I(t) \rangle$ were recorded and analyzed by an autocorrelation function $G(\tau) = 1 + \langle \delta I(t) \cdot \delta I(t+\tau) \rangle / \langle I(t) \rangle^2$. The obtained experimental autocorrelation curves were fitted with the analytical expression for freely diffusing fluorescence species:

$$G(\tau) = 1 + \left[1 + \frac{f_T}{1-f_T} e^{-\tau/\tau_T}\right] \frac{1}{N} \frac{1}{\left[1+\frac{\tau}{\tau_D}\right]\sqrt{1+\frac{\tau}{S^2\tau_D}}} \qquad \text{Equation (1)}$$

Here, N is the average number of diffusing fluorescence species in the observation volume, and f_T and τ_T are the fraction and decay time of the triplet state, respectively. τ_D is the diffusion time of the species, and S is the structure parameter, $S = z_0/r_0$, where z_0 and r_0 represent the axial and radial dimensions of the confocal volume, respectively. Furthermore, the diffusion time, τ_D, is related to the respective diffusion coefficient, D, through: $\tau_D = \frac{r_0^2}{4D}$. The fitting yielded the corresponding diffusion times and subsequently the diffusion coefficients of the fluorescently labeled P-S in the water phase and P1 gel. The diffusion coefficient and hydrodynamic radius of the fluorescently labeled P-S were obtained using the Stokes-Einstein equation. As the value of r_0 depends strongly on the specific characteristics of the optical setup, a calibration was performed using a fluorescent tracer (Alexa Fluor 488) that has a known diffusion coefficient[1,2].

Synthesis

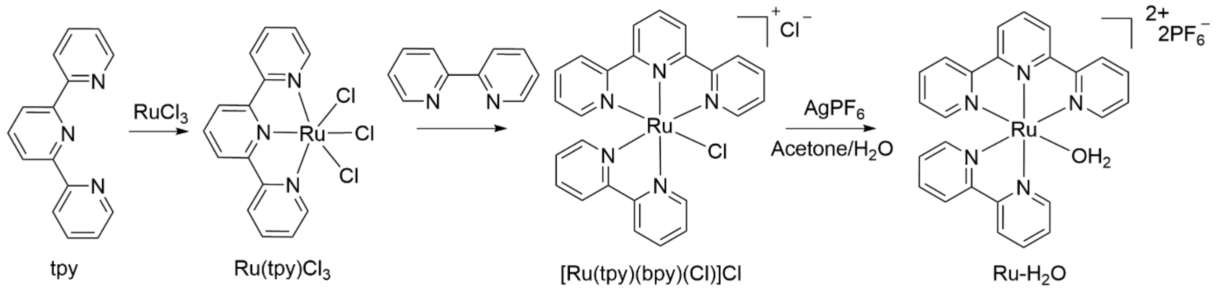

Supplementary Figure 1. Synthetic route for Ru-H_2O.

Synthesis of Ru(tpy)Cl_3: $RuCl_3 \cdot xH_2O$ (262 mg, 1.0 mmol) and 2,2':6',2''-terpyridine (tpy, 233.3 mg, 1.0 mmol) were dissolved in ethanol (50 mL). The mixture was heated at reflux for 4 h with magnetic stirring. Then, the mixture was cooled to room temperature. Brown powders were obtained and filtered from the reddish-yellow solution. The brown powders were washed with ethanol and diethyl ether, and dried to obtain the compound Ru(tpy)Cl_3 as a brown solid. Yield: 363 mg (82%).

Synthesis of [Ru(tpy)(bpy)(Cl)]Cl: Ru(tpy)Cl_3 (171 mg, 0.39 mmol) and 2,2'- bipyridine (bpy, 61 mg, 0.39 mmol) were mixed in a 2/1 (v/v) ethanol/H_2O mixture (30 mL). The solution was degassed for 5 min and filled with argon before refluxing at 85 ℃ for 24 h under an argon atmosphere. After that, the mixture was filtered hot, and the filtrate was evaporated under reduced pressure to obtain the crude product. The product was purified by column chromatography with silica gel (eluent: methanol/dichloromethane (v/v) = 1/8 to 1/4). The solvent was evaporated and the product was obtained as violet powders. Yield: 180 mg (77.6%).

^{1}H NMR of [Ru(tpy)(bpy)(Cl)]Cl (300 MHz, CD_3OD, 25°C) δ (ppm): 10.21 (d, J = 4.7 Hz, 1H, H-A1), 8.79 (d, J = 8.8 Hz, 1H, H-A4), 8.67 (d, J = 8.8 Hz, 2H, H-T2'), 8.56-8.51 (dd, J = 7.8 Hz, 3H, H-T4, B4), 8.34 (t, J = 8.4 Hz, 1H, H-A3), 8.17 (t, J = 8.8 Hz, 1H, H-T3'), 8.02 (t, J = 7.8 Hz, 1H, H-A2), 7.95 (t, J = 7.8 Hz, 2H, H-T3), 7.77-7.70 (m, 3H, H-B3, 2H-T1), 7.40-7.32 (m, 2H-T2, H-B1), 7.05 (t, J = 7.4 HZ, 1H, H-B2).

^{13}C NMR of [Ru(tpy)(bpy)(Cl)]Cl (75 MHz, CD_3OD, 25 °C) δ (ppm): 158. 96 (T1', T5), 158.17 (B5), 156.25 (A5), 152.18 (B1), 151.98 (T1), 151.38 (A1), 137.21 (T3), 136.52 (T3'), 135.57 (B3), 134.16 (A3), 127.18 (T2), 126.73 (B2), 126.08 (A2), 123.37 (T4), 123.07 (B4), 125.98 (A4) and 122.24 (T2').

MALDI-TOF-MS of [Ru(tpy)(bpy)(Cl)]Cl: *m/z* calculated for $C_{25}H_{19}N_5Cl_2Ru$ ($[M-Cl]^+$): 526.04; found 525.03.

UV-vis of [Ru(tpy)(bpy)(Cl)]Cl: The absorption maximum in acetone is at 507 nm, which is attributed to the metal-to-ligand charge transfer (MLCT) band of [Ru(tpy)(bpy)(Cl)]Cl.

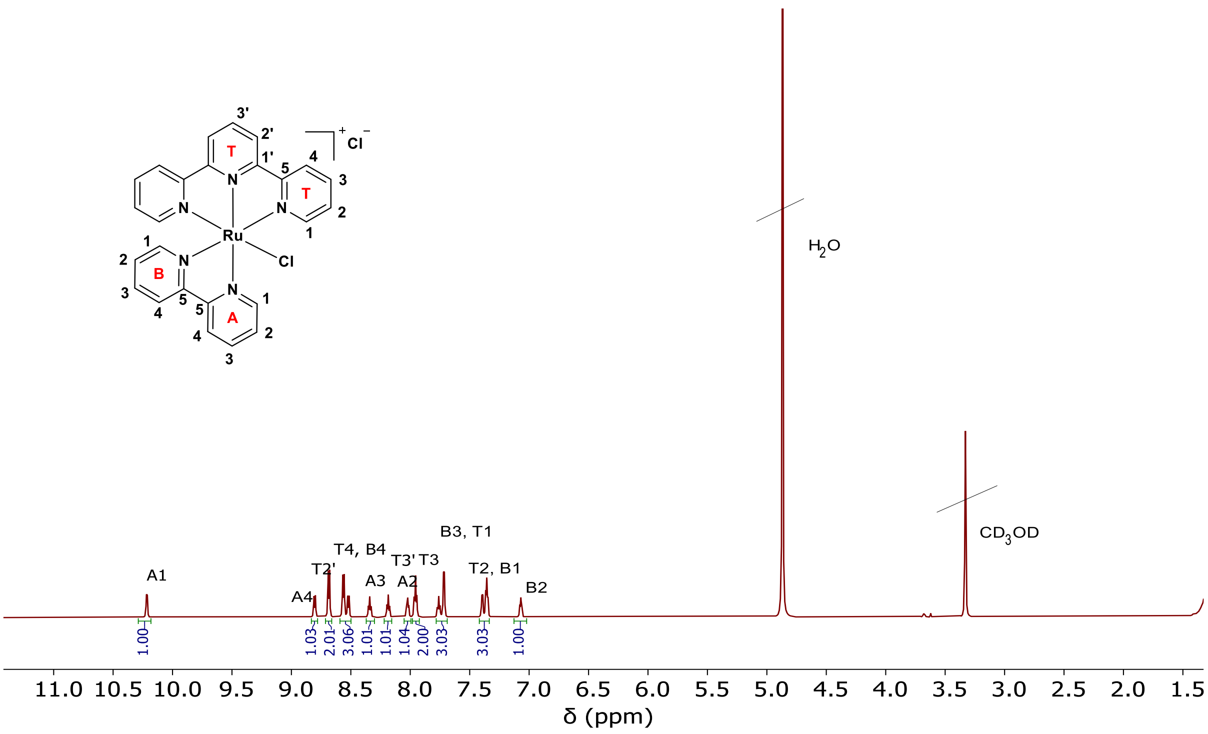

Supplementary Figure 2. ^{1}H NMR spectrum of [Ru(tpy)(bpy)(Cl)]Cl (300 MHz, CD_3OD, 25°C).

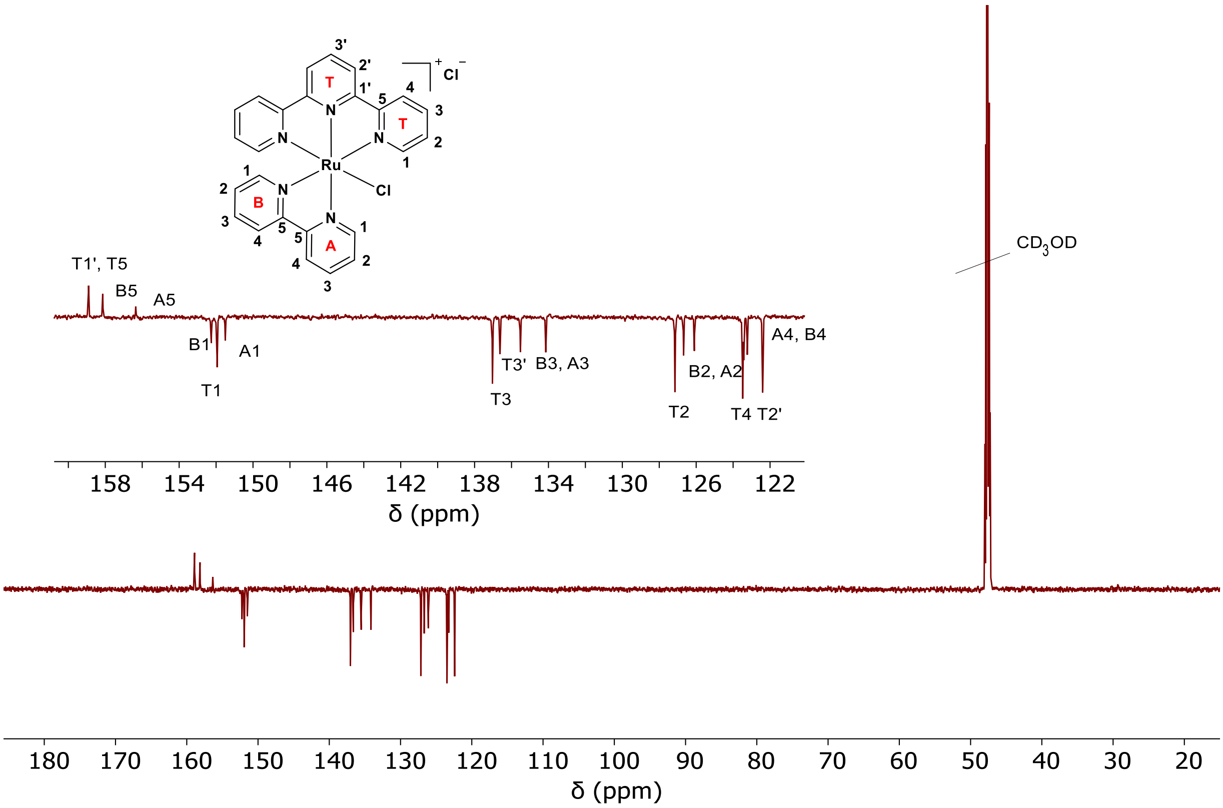

Supplementary Figure 3. ^{13}C NMR spectrum of [Ru(tpy)(bpy)(Cl)]Cl (75 MHz, CD_3OD, 25°C).

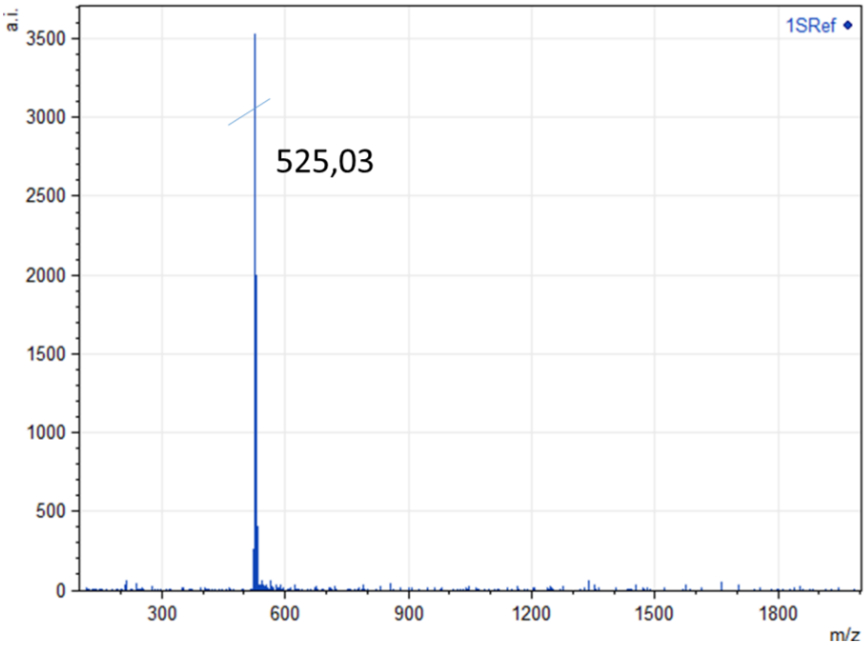

Supplementary Figure 4. MALDI-TOF-MS spectrum of [Ru(tpy)(bpy)(Cl)]Cl.

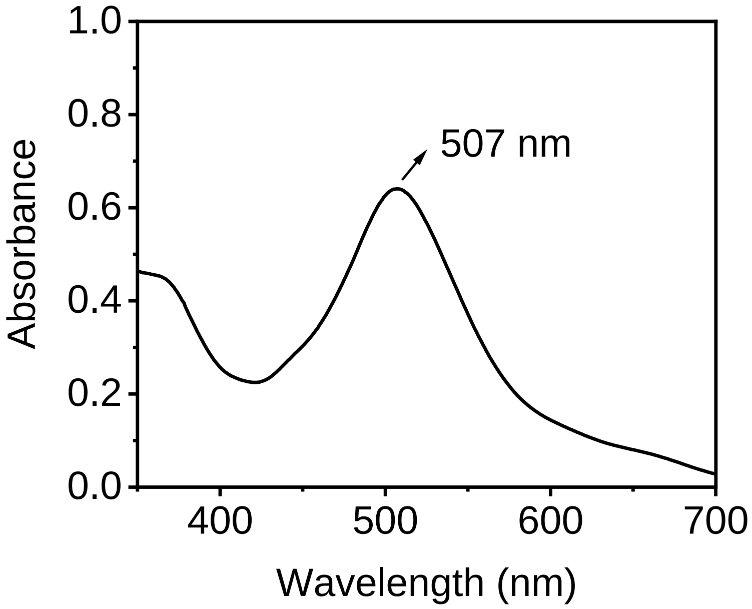

Supplementary Figure 5. UV-vis absorption spectrum of [Ru(tpy)(bpy)(Cl)]Cl in acetone.

Synthesis of Ru-H_2O: [Ru(tpy)(bpy)(Cl)]Cl (28 mg, 0.049 mmol) and $AgPF_6$ (28 mg, 0.11 mmol) were mixed in a 3/1 (v/v) H_2O/acetone mixture (8 mL). The solution was degassed for 5 min and filled with argon before refluxing at 85 °C for 24 h under an argon atmosphere. After that, the mixture was filtered over Celite and concentrated to 1 mL under reduced pressure. The

dark red solid was precipitated from a saturated KPF_6 aqueous solution (26 mg, 0.032 mmol, 66.4%).

^{1}H NMR of Ru-H_2O (300 MHz, D_2O, 25°C) δ (ppm): 9.50 (d, J = 5.3 Hz, 1H, H-A1), 8.58 (d, J = 5.5 Hz, 1H, H-A4), 8.67 (d, J = 4.6 Hz, 2H, H-T2'), 8.36 (d, J = 4.5 Hz, 2H, H-T4), 8.27-8.22 (m, 2H, H-B4, H-T3'), 8.13 (t, J = 7.3 Hz, 1H, H-A3), 7.96-7.85 (m, 3H, H-A2, 2H-T3), 7.71 (d, J = 5.5 Hz, 2H, H-T1), 7.58 (t, J = 7.6 Hz, 1H, H-B3), 7.23 (m, 3H, 2H-T2, H-B1), 6.84 (t, J = 7.7 Hz, 1H, H-B2).

^{13}C NMR of Ru-H_2O (75 MHz, CD_3OD, 25 °C) δ (ppm): 159.24 (B5), 159.07 (T1'), 158.66 (T5), 156.03 (A5), 152.88 (T1), 152.34 (B1), 150.22 (A1), 138.46 (T3), 137.51 (T3'), 136.29 (B3), 136.08 (A3), 128.10 (T2), 127.52 (B2), 126.39 (A2), 124.21 (T4), 124.00 (B4), 123.38 (A4) and 123.30 (T2').

MALDI-TOF-MS of Ru-H_2O: *m/z* calculated for $C_{25}H_{21}N_5ORuP_2F_{12}$ ($[M\text{-}2PF_6\text{-}H]^+$): 508.55; found 508.09.

UV-vis of Ru-H_2O: The absorption maximum in water is at 476 nm, which is attributed to the MLCT band of Ru-H_2O.

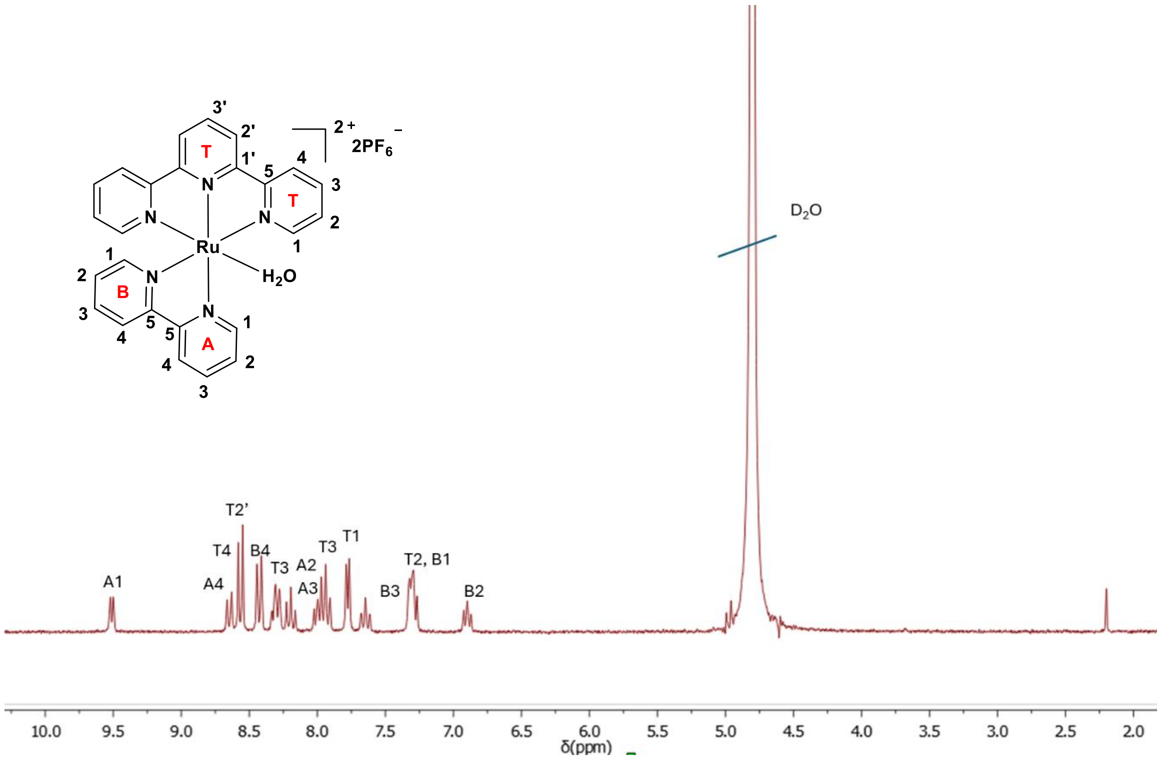

Supplementary Figure 6. ^{1}H NMR spectrum of Ru-H_2O (300 MHz, D_2O, 25°C).

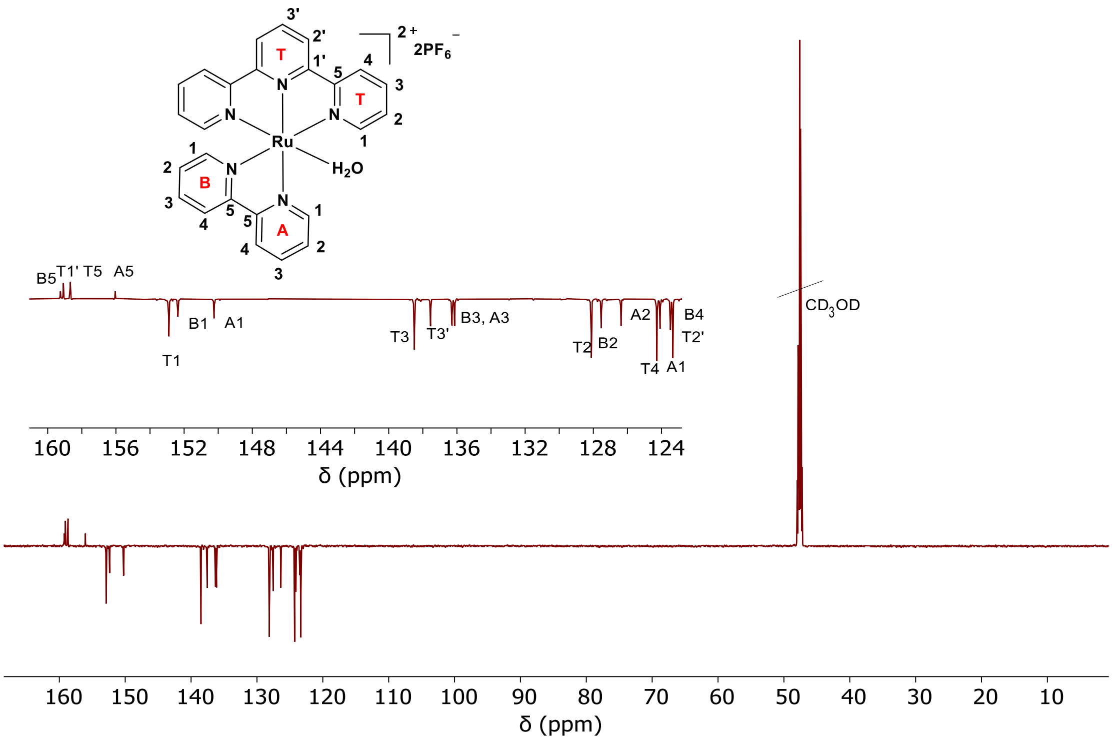

Supplementary Figure 7. ^{13}C NMR spectrum of Ru-H_2O (75 MHz, CD_3OD, 25°C).

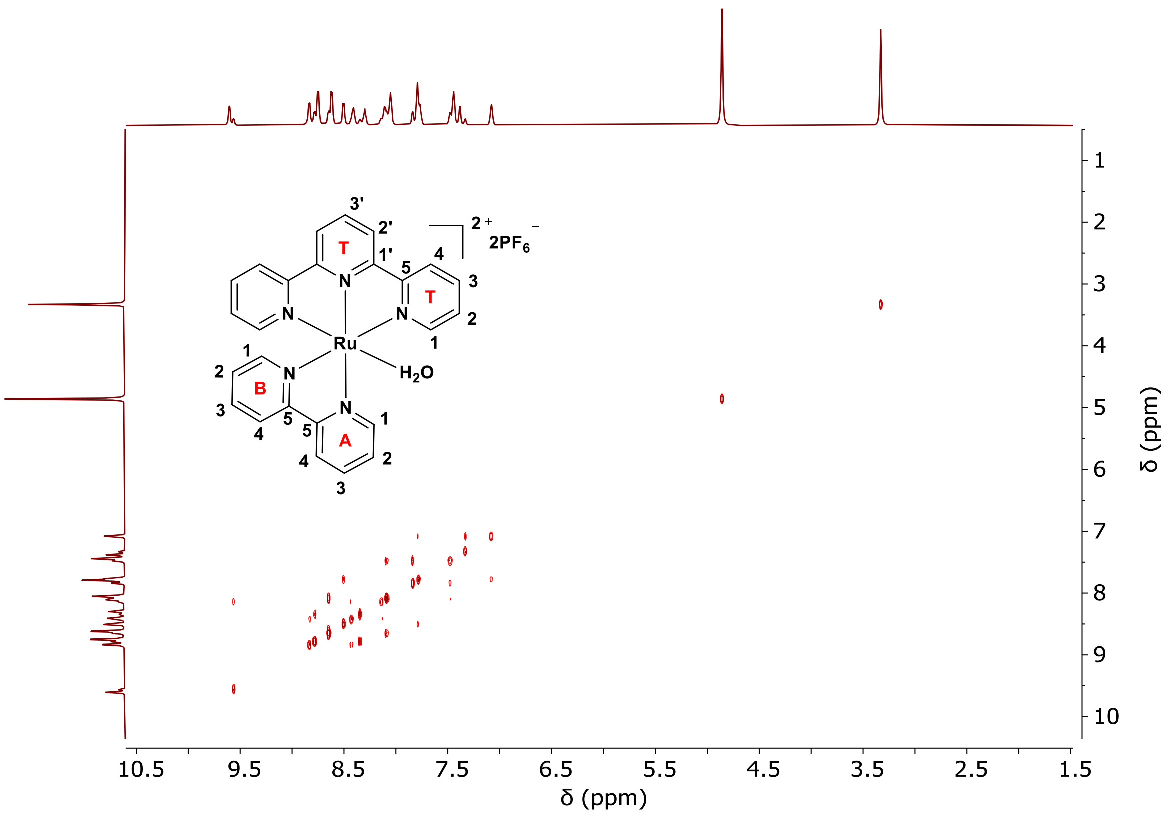

Supplementary Figure 8. H-H COSY spectrum of Ru-H_2O (300 MHz, CD_3OD, 25°C).

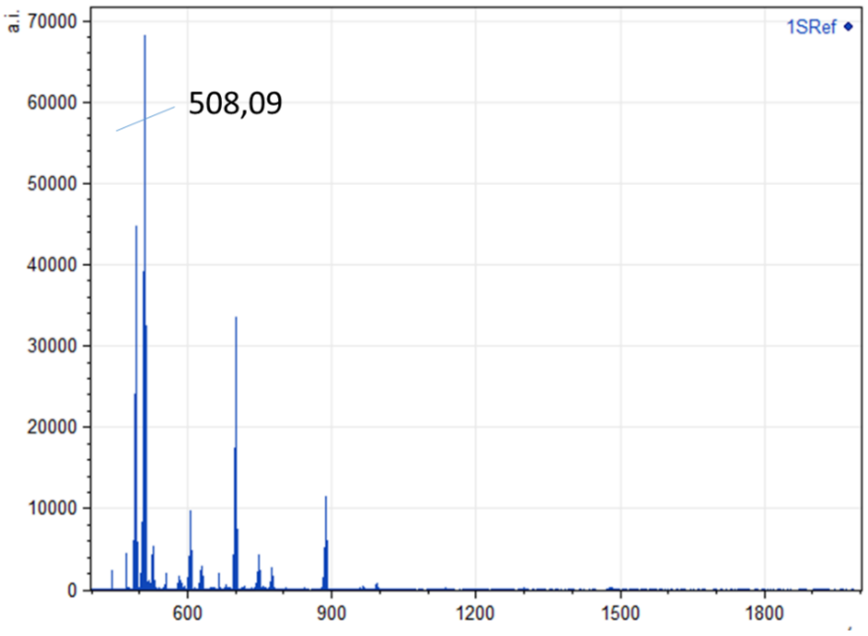

Supplementary Figure 9. MALDI-TOF-MS spectrum of Ru-H_2O.

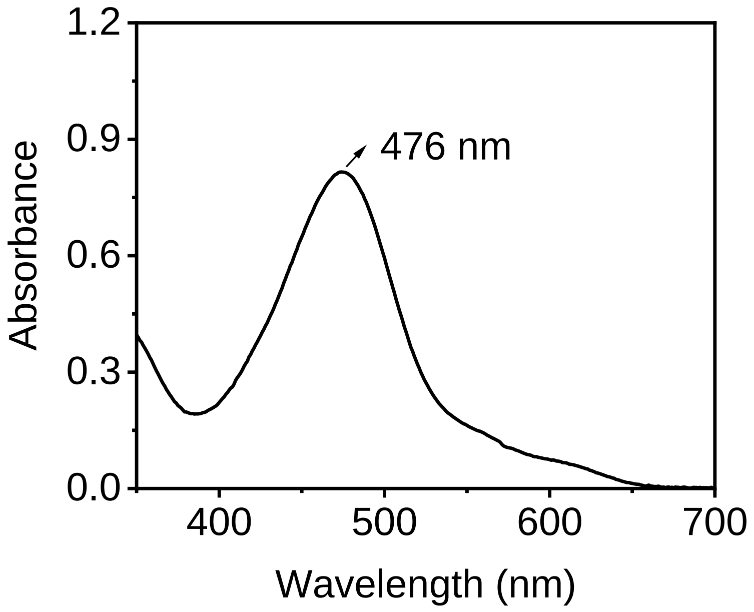

Supplementary Figure 10. UV-vis absorption spectrum of Ru-H_2O in water.

Synthesis of 2-(2-(2-(methylthio)ethoxy)ethoxy)ethanol (SL)

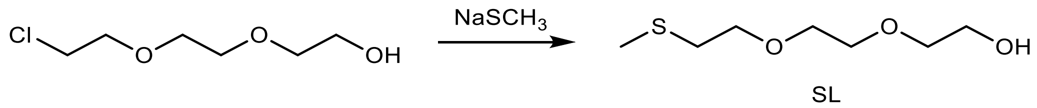

Supplementary Figure 11. Synthetic route for compound SL.

Synthesis of 2-(2-(2-(methylthio)ethoxy)ethoxy)ethanol (SL): Sodium thiomethoxide (2 g, 28 mmol) was added to 30 mL deionized water and stirred for 10 min. 2-[2-(2-Chloroethoxy)ethoxyl]ethanol (2.36 g, 14 mmol) was added to the flask, and the mixture was stirred for 2 days at room temperature. After the reaction, sodium chloride was added to the mixture until saturation. Then, the mixture was extracted with dichloromethane. The organic layer was dried with magnesium sulfate and concentrated under reduced pressure. The product was a colorless oil. Yield: 1.84 g (65%).

^{1}H NMR of SL (300 MHz, D_2O, 25°C) δ (ppm): 3.65 (m, 10H, 2H-3, 2H-4, 2H-5, 2H-6, 2H-7), 2.71 (t, J = 6.4 Hz, 2H, H-2), 2.11 (S, 3H, H-1).

^{13}C NMR of SL (75 MHz, D_2O, 25 °C) δ (ppm): 71.78 (7), 69.52 (6), 69.50 (5), 69.47 (4), 69.12 (3), 32.61 (2) and 14.49 (1).

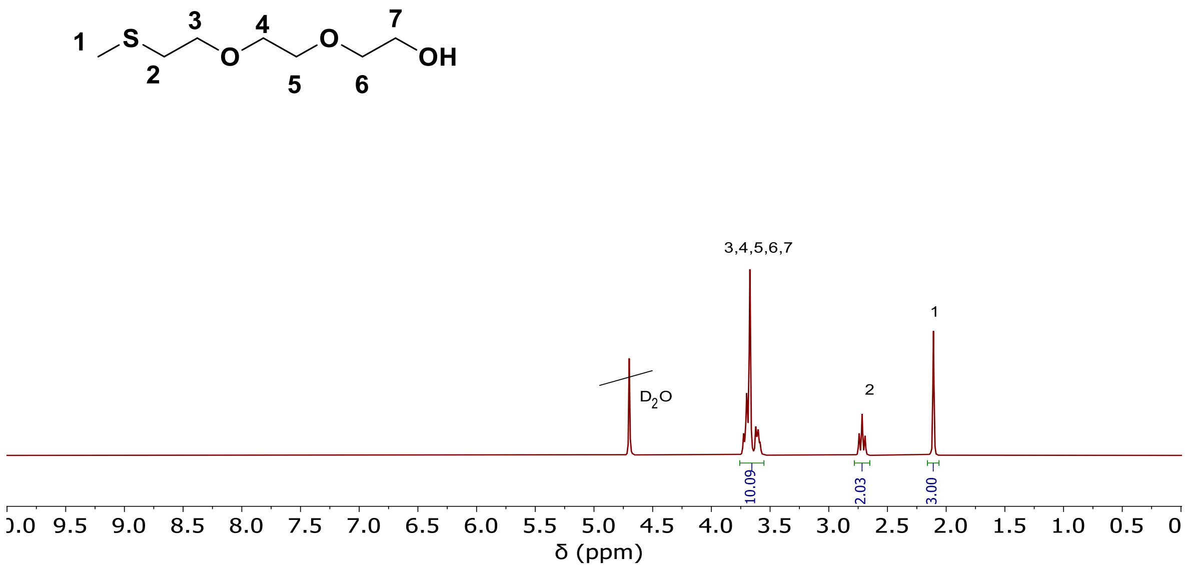

Supplementary Figure 12. ^{1}H NMR spectrum of SL (300 MHz, D_2O, 25°C).

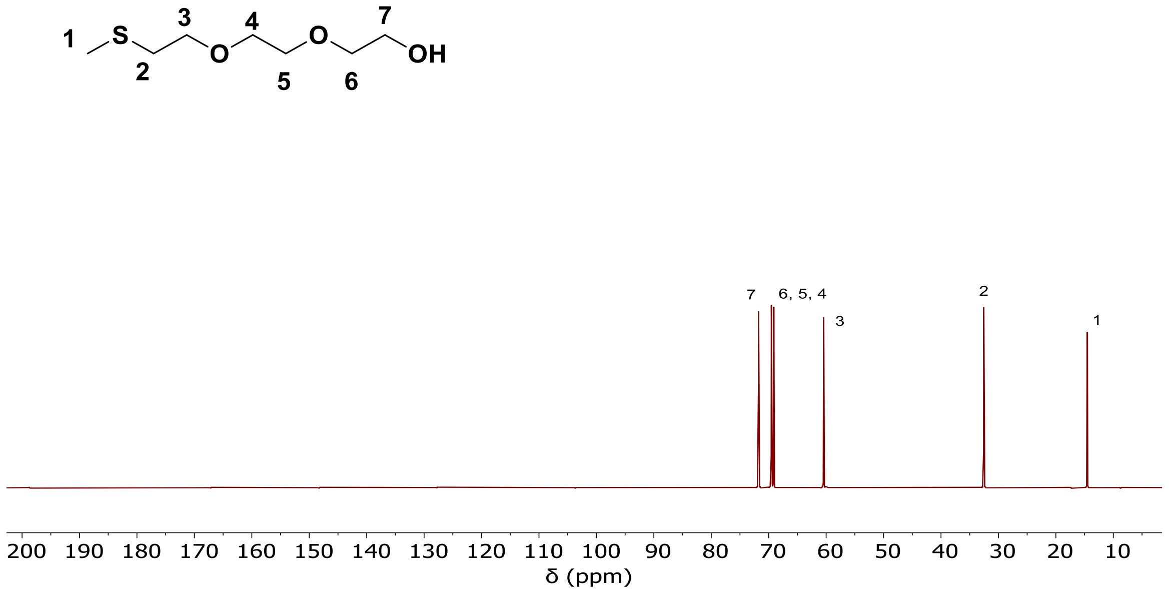

Supplementary Figure 13. ^{13}C NMR spectrum of SL (75 MHz, D_2O, 25°C).

Synthesis of Ru-SL

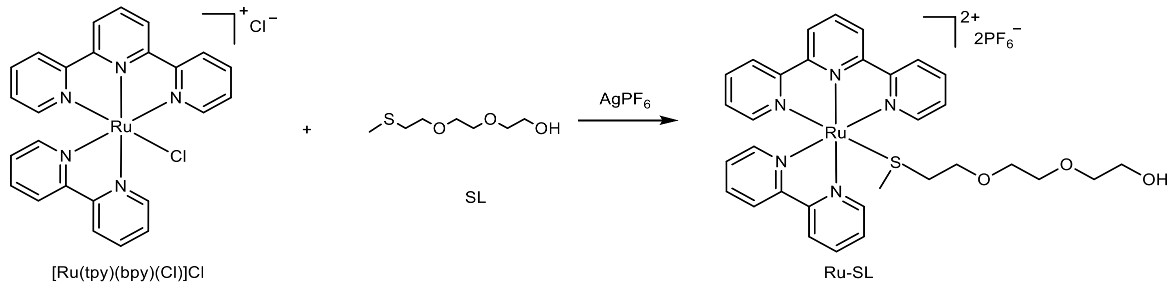

Supplementary Figure 14. Synthetic route for Ru-SL.

Synthesis of Ru-SL: [Ru(tpy)(bpy)(Cl)]Cl (28 mg, 0.049 mmol) and $AgPF_6$ (28 mg, 0.11 mmol) were dissolved in a 3/5 (v/v) acetone/H_2O mixture (8 mL). SL (90 mg, 0.5 mmol) was added to the mixture solution. The mixture was refluxed at 85 °C under argon for 12 h. After that, it was filtered hot over Celite and concentrated to 1 mL under reduced pressure. The red organic solid was precipitated from a saturated KPF_6 solution. Yield: 23 mg, 0.023 mmol (46.9%)

^{1}H NMR of Ru-SL (300 MHz, D_2O, 25°C) δ (ppm): 9.76 (d, J = 4.1 Hz, 1H, H-A1), 8.60-8.54 (m, 3H, H-A4, 2H-T2'), 8.40-8.21 (m, 5H, 2H, H-T4, H-B4, H-A3, H-T3'), 7.96-7.87 (m, 3H, H-A2, 2H-T3), 7.79-7.70 (m, 3H, H-B3, 2H-T1), 7.25 (t, J = 5.2 Hz, 2H, H-T2) 7.16 (d, J = 3.5 Hz, 1H, H-B1), 6.99 (t, J = 7.0 Hz, 1H, H-B2), 3.59-3.25 (m, 10H, 2H-c, 2H-d, 2H-e, 2H-f, 2H-g), 1.78 (t, J = 7.6, 2H, H-b), 1.28 (s, 3H, H-a).

^{13}C NMR of Ru-SL (75 MHz, acetone-d_6, 25 °C) δ (ppm): 158.06 + 157.56 (T1', T5), 156.88 + 156.78(B5, A5), 153.60 (T1), 152.37 (B1), 150.12 (A1), 139.98 (T2), 138.29 + 138.16 (B3, A3), 137.03 (T3'), 128.61 (T3), 127.99 + 127.28 (B2, A2), 125.07 (T4), 124.74 (A4), 124.39 (T2'),123.90 (B4), 72.52 (g), 70.05 + 69.84 (e, f), 67.22 (d), 60.92 (c), 34.45 (b), 14.44 (a).

ESI-Mass of Ru-SL: *m/z* calculated for $C_{32}H_{37}N_5O_3RuSP_2F_{12}$ ($[M\text{-}2PF_6\text{-}H]^+$): 817.17; found 816.4.

UV-vis absorption spectrum of Ru-SL: The absorption maximum in water is at 452 nm, which is attributed to the MLCT band of Ru-SL.

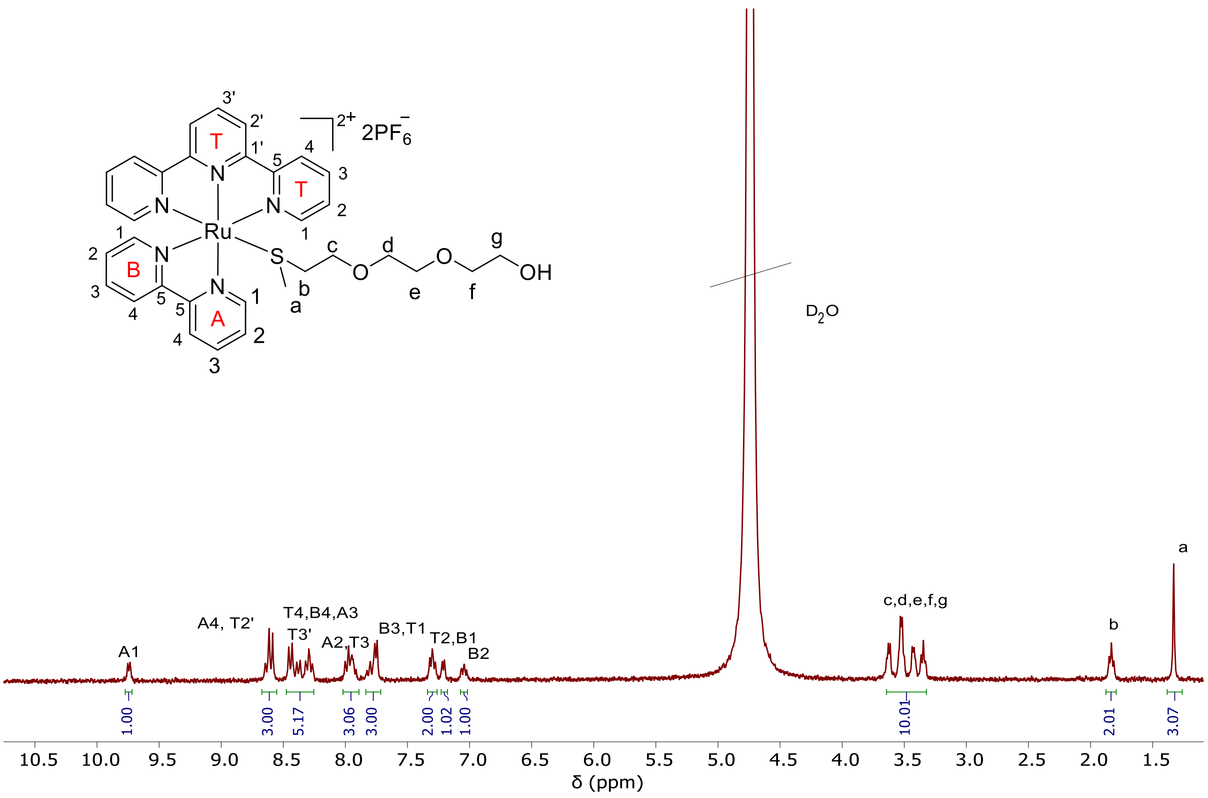

Supplementary Figure 15. ^{1}H NMR spectrum of Ru-SL (300 MHz, D_2O, 25°C)

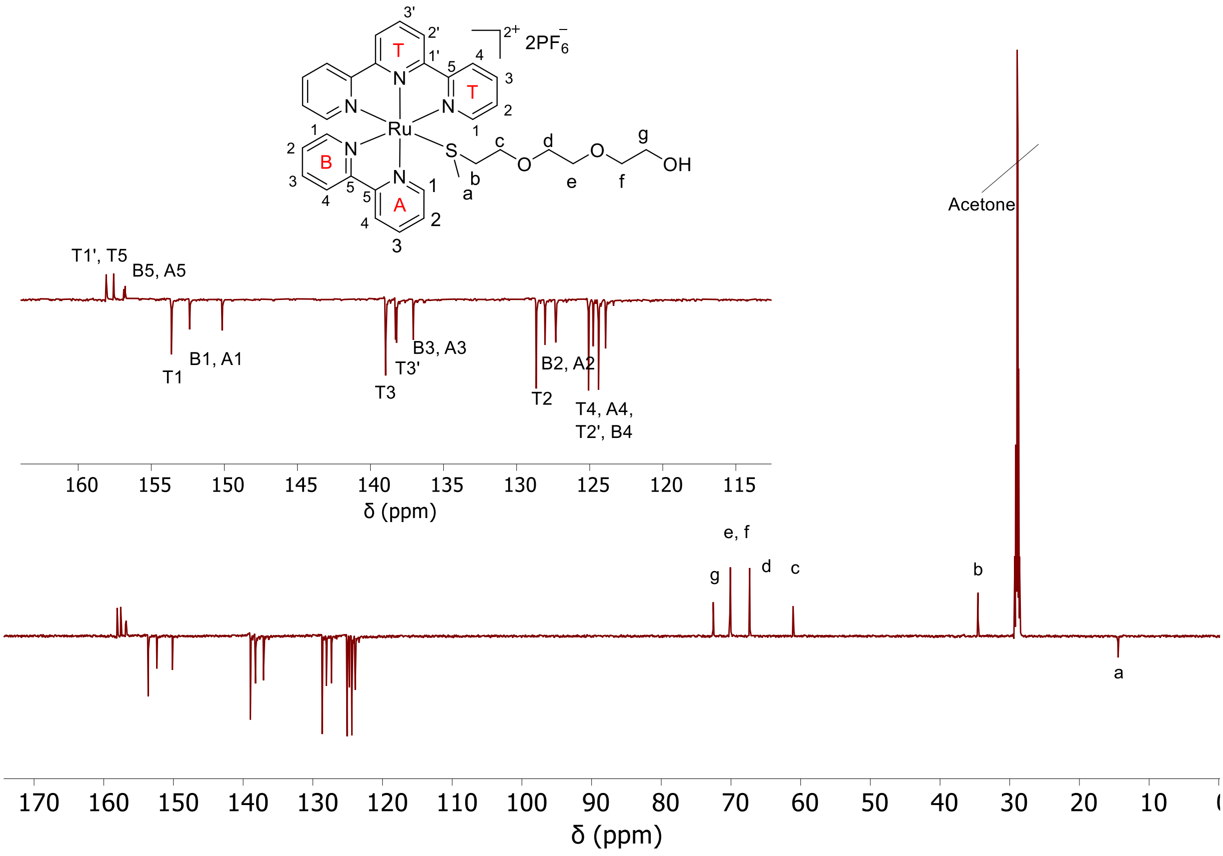

Supplementary Figure 16. ^{13}C NMR spectrum of Ru-SL (75 MHz, acetone-d_6, 25°C).

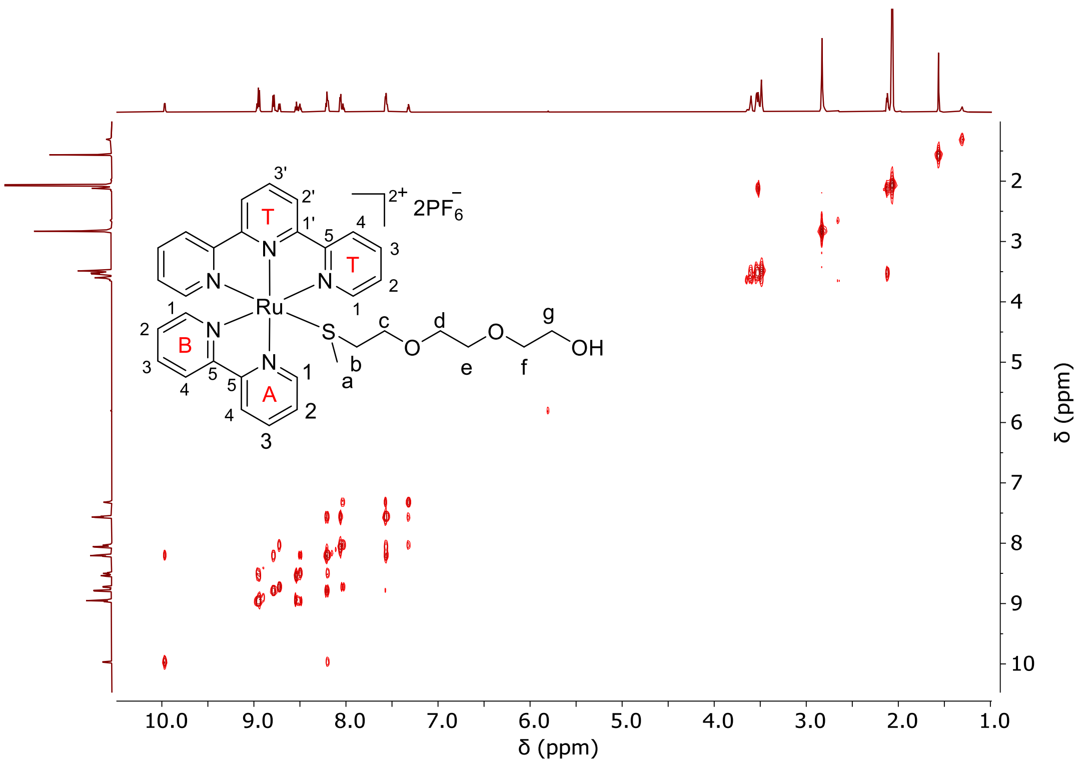

Supplementary Figure 17. H-H COSY spectrum of Ru-SL (300 MHz, acetone-d_6, 25°C).

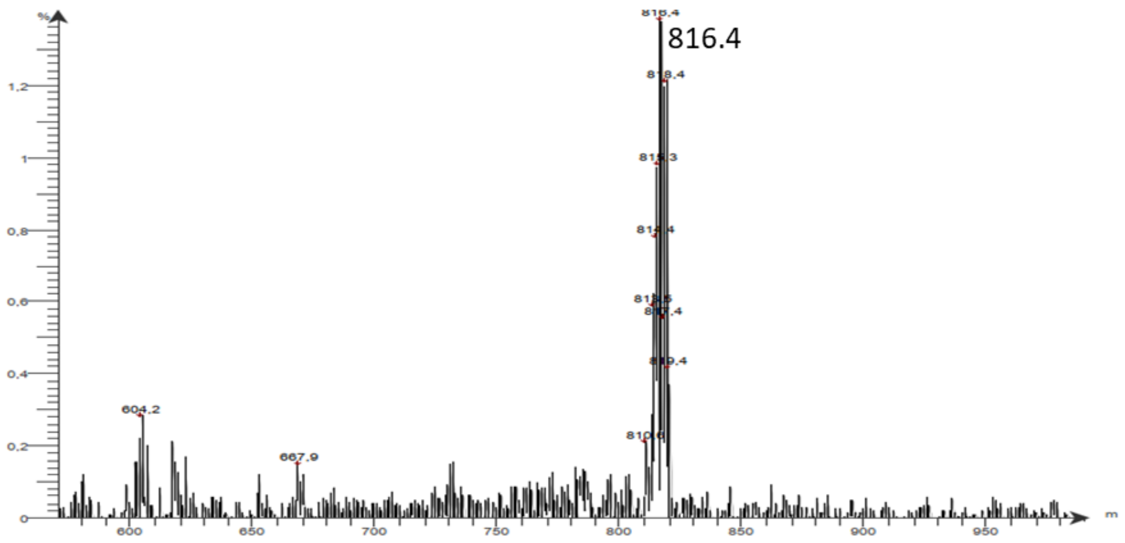

Supplementary Figure 18. ESI-Mass spectrum of Ru-SL.

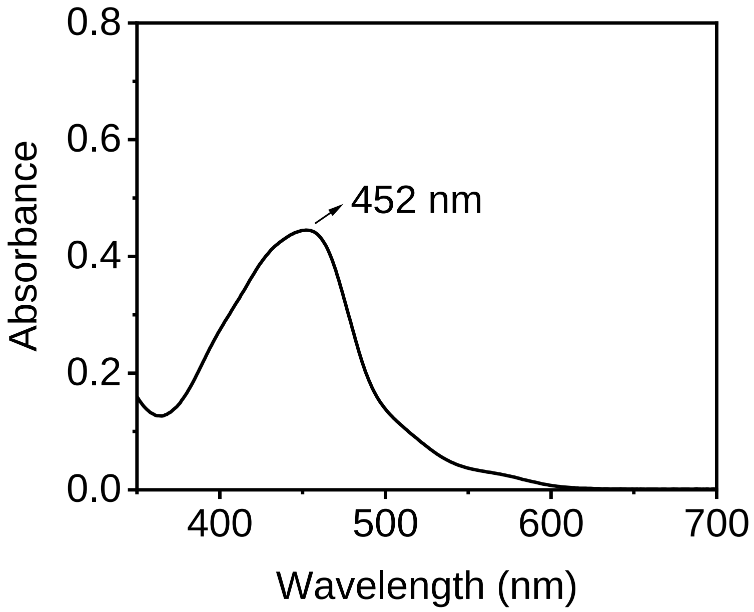

Supplementary Figure 19. UV-vis absorption spectrum of Ru-SL in water.

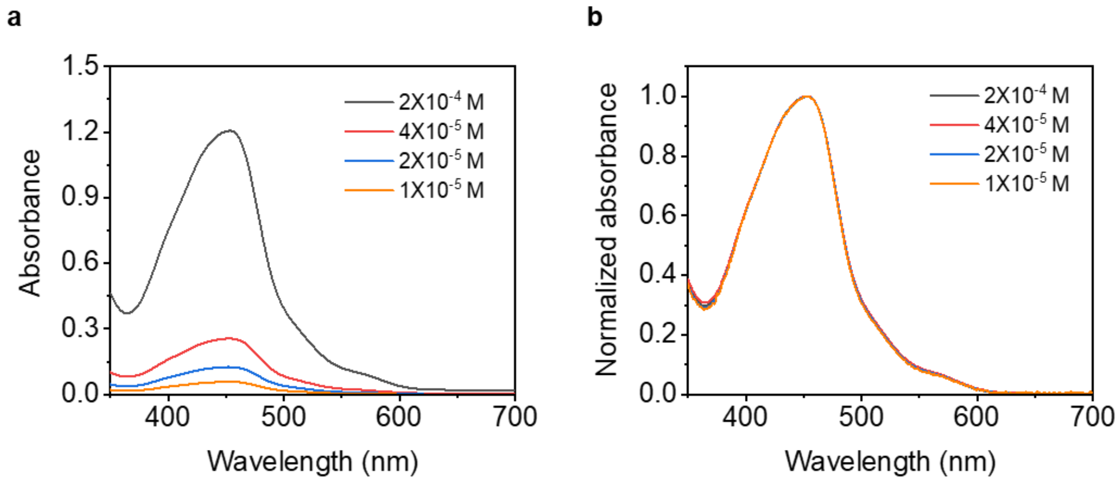

Supplementary Figure 20. UV-vis absorption spectra (a) and normalized absorption spectra (b) of Ru-SL in water before and after being diluted 5, 10 and 20 times. The initial concentration of Ru-SL was 2×10^{-4} M. The absorption band did not change upon dilution. It is well known that the dissociation of the Ru-S coordination bond causes a shift in the absorption band. No change in the absorption band indicates that the Ru-S coordination bond is stable in water even after dilution 20 times.

The Ru-SL coordination reported here is robust yet reversible. The Ru-SL compound could be isolated (Supplementary Figs. 14-19), which revealed that it is more stable than those compounds that could not be isolated. The success of this work is because we designed the Ru moiety with a 2,2'-bipyridine ligand that avoids distortion of the structure and weakening of the coordination bond.

Synthesis of [Ru(AAm-tpy)(bpy)Cl]Cl

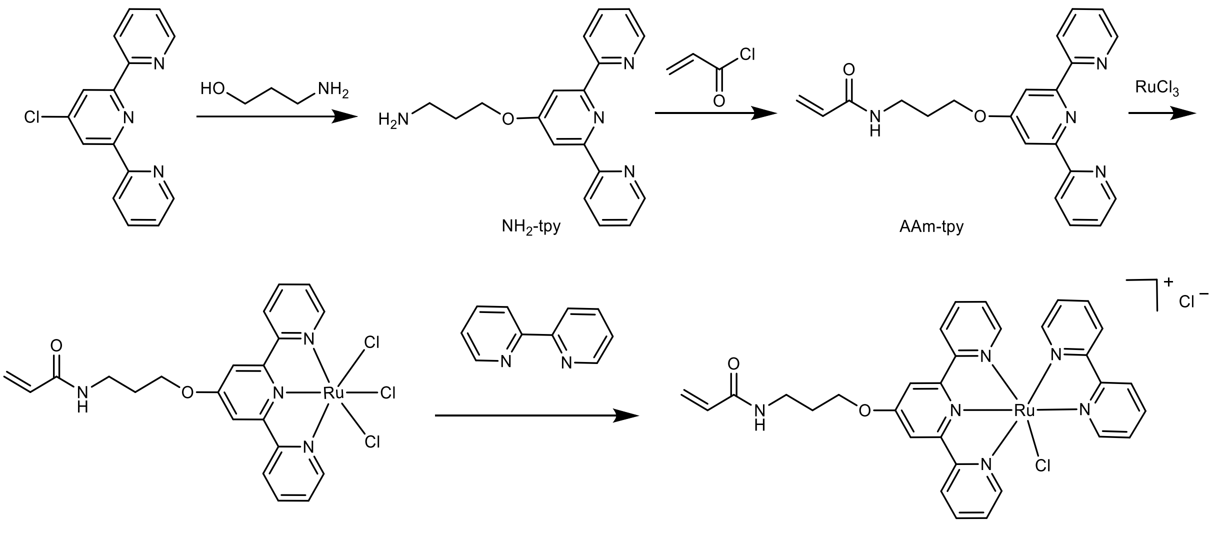

Supplementary Figure 21. Synthetic route for [Ru(AAm-tpy)(bpy)Cl]Cl.

Synthesis of 3-([2, 2': 6',2''-terpyridin]-4'-yloxy)propane-1-amine (NH$_2$-tpy): Potassium hydroxide (392 mg, 7.0 mmol) was added to dimethyl sulfoxide (20 mL) and heated to 65 °C under stirring. 3-Amino-1-propanol (555 mg, 7.4 mmol) was added dropwise into the solution. After 30 min, 4'-chloro-2,2',6',2''-terpyridine (1000 mg, 3.7 mmol) was added to the solution. After stirring for 2 days at 65 °C, the reaction solution was cooled to room temperature and poured into 200 mL deionized water. The crude product was extracted with dichloromethane and dried with magnesium sulfate. After filtration and removal of the solvent under vacuum, the crude product was recrystallized from ethyl acetate to obtain light-yellow solids. Yield: 1900 mg (84%).

^{1}H NMR of NH_2-tpy (300 MHz, CD_2Cl_2, 25°C) δ (ppm): 8.70 (d, J = 5.4 Hz, 2H, H-2), 8.65 (d, J = 7.0 Hz, 2H, H-5), 8.06 (s, 2H, H-3'), 7.90 (t, J = 7.7 Hz, 2H, H-4), 7.37 (t, J = 7.7 Hz, 2H, H-3), 4.35 (t, J = 5.7 Hz, 2H, H-7), 2.95 (t, J = 8.0 Hz, 2H, H-9), 2.02 (m, 2H, H-8).

^{13}C NMR of NH_2-tpy (75 MHz, CD_3OD, 25°C) δ (ppm): 168.49 (4'), 158.32 (2'), 157.00 (6), 150.60 (2), 138.57 (4), 125.41 (3), 122.96 (5), 108.48 (3'), 67. 62 (7), 39.38 (9) and 32.44 (8).

MALDI-TOF-MS of NH_2-tpy: *m/z* calculated for $C_{18}H_{18}N_4O$ ($[M]^+$): 306.37; found 306.96.

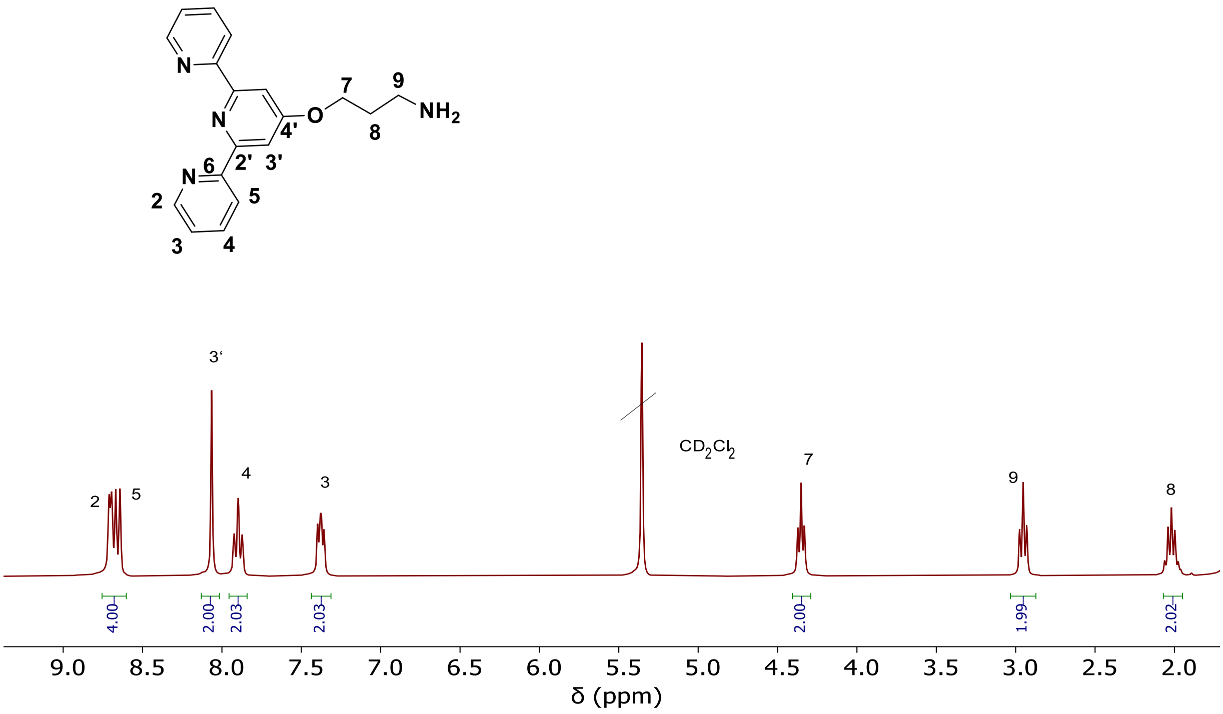

Supplementary Figure 22. ^{1}H NMR spectrum of NH_2-tpy (300 MHz, CD_2Cl_2, 25°C).

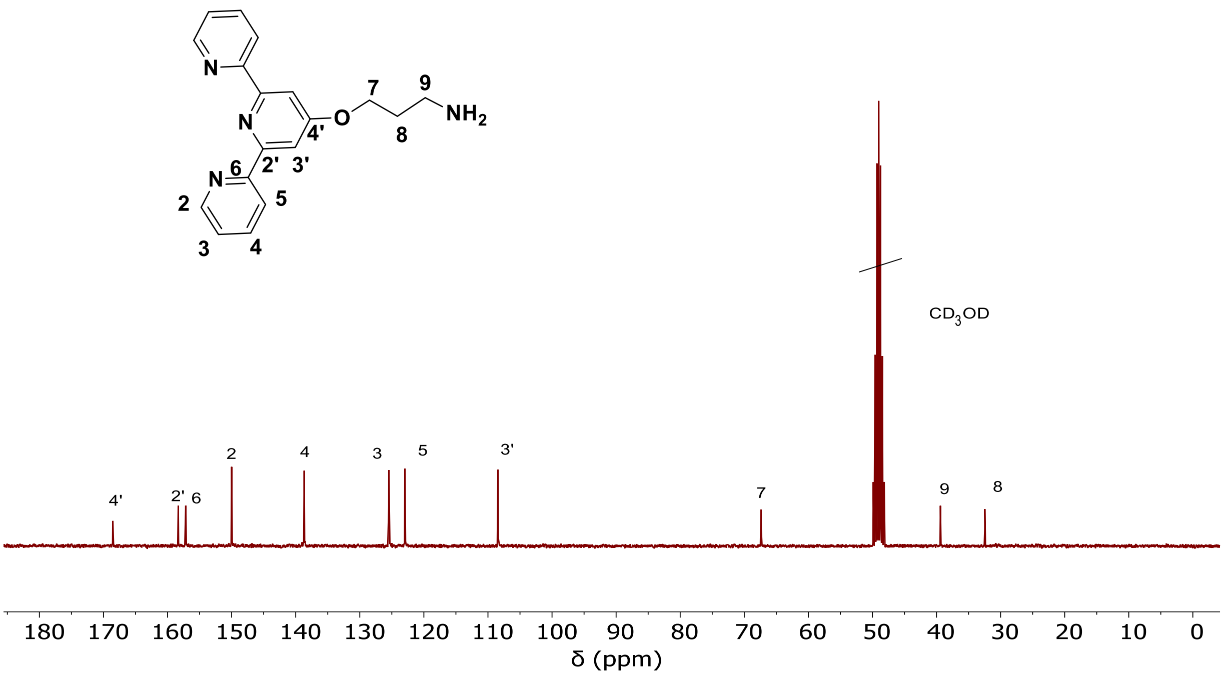

Supplementary Figure 23. ^{13}C NMR spectrum of NH_2-tpy (75 MHz, CD_3OD, 25°C).

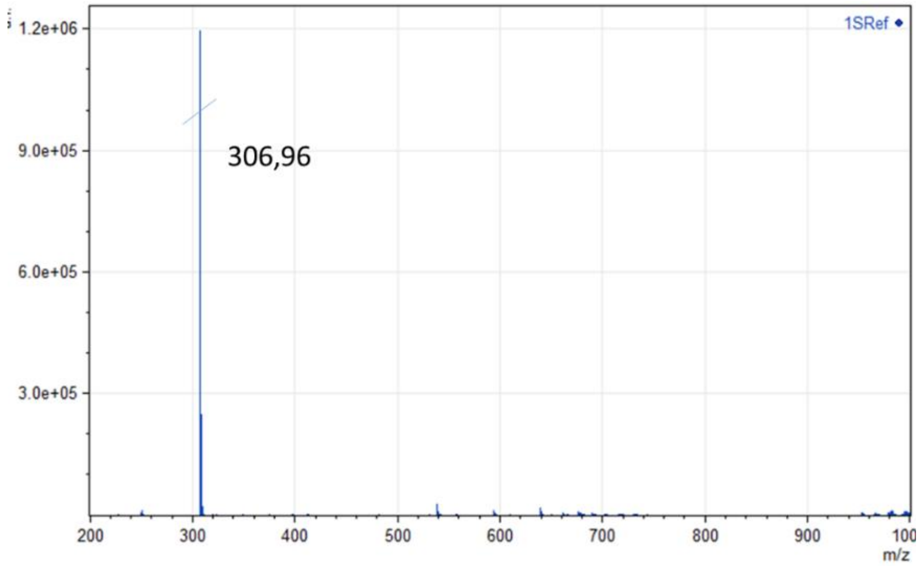

Supplementary Figure 24. MALDI-TOF-MS spectrum of NH_2-tpy ($[M]^+$: found 306.96).

Synthesis of N-(3-([2,2':6',2''-terpyridin]-4'-yloxy)propyl)acrylamide (AAm-tpy): NH_2-tpy (1360 mg, 4.7 mmol) was added to 40 mL tetrahydrofuran. Triethylamine (1.8 mL) was added to the solution. Then, acryloyl chloride (607 mg, 6.71 mmol) was slowly added under argon at 0 °C after the mixture was stirred for 30 min. The reaction solution was stirred for 2 h

at 0 °C and stirred for another 21 h at room temperature. After that, the solvent was removed under vacuum, and the residue was dissolved in dichloromethane and washed with water and saturated brine. The organic solution was dried with magnesium sulfate, filtered, and reduced in vacuum to give crude solids. The crude solids were purified by column chromatography (Al_2O_3) using methanol/dichloromethane (v/v, 1/40) as the eluent. The solvent was evaporated and the product was obtained as a light-yellow solid. Yield: 1293 mg (77%).

^{1}H NMR of AAm-tpy (300 MHz, CD_2Cl_2, 25°C) δ (ppm): 8.70 (d, J = 4.7 Hz, 2H, H-2), 8.61 (d, J = 8.0 Hz, 2H, H-5), 8.02 (s, 2H, H-3'), 7.85 (t, J = 6.0 Hz, 2H, H-4), 7.34 (t, J = 6.0 Hz, 2H, H-3), 6.25 (m, 2H, H-11, H-12), 5.64 (dd, J = 11.5 Hz, 1H, H-12), 4.34 (t, J = 6.0 Hz, 2H, H-7), 3.05 (t, J = 6.75 Hz, 2H, H-9), 2.09 (m, 2H, H-8).

^{13}C NMR of AAm-tpy (75 MHz, CD_2Cl_2, 25°C) δ (ppm): 168.49 (4'), 167 (10), 158.32 (2'), 157.00 (6), 150.60 (2), 138.57 (4), 132 (11), 127 (12), 125.41 (3), 122.96 (5), 108.48 (3'), 67 (7), 39.38 (9) and 32.44 (8).

MALDI-TOF-MS of AAm-tpy: *m/z* calculated for $C_{21}H_{20}N_4O_2$ ($[M]^+$): 360.42; found 360.83.

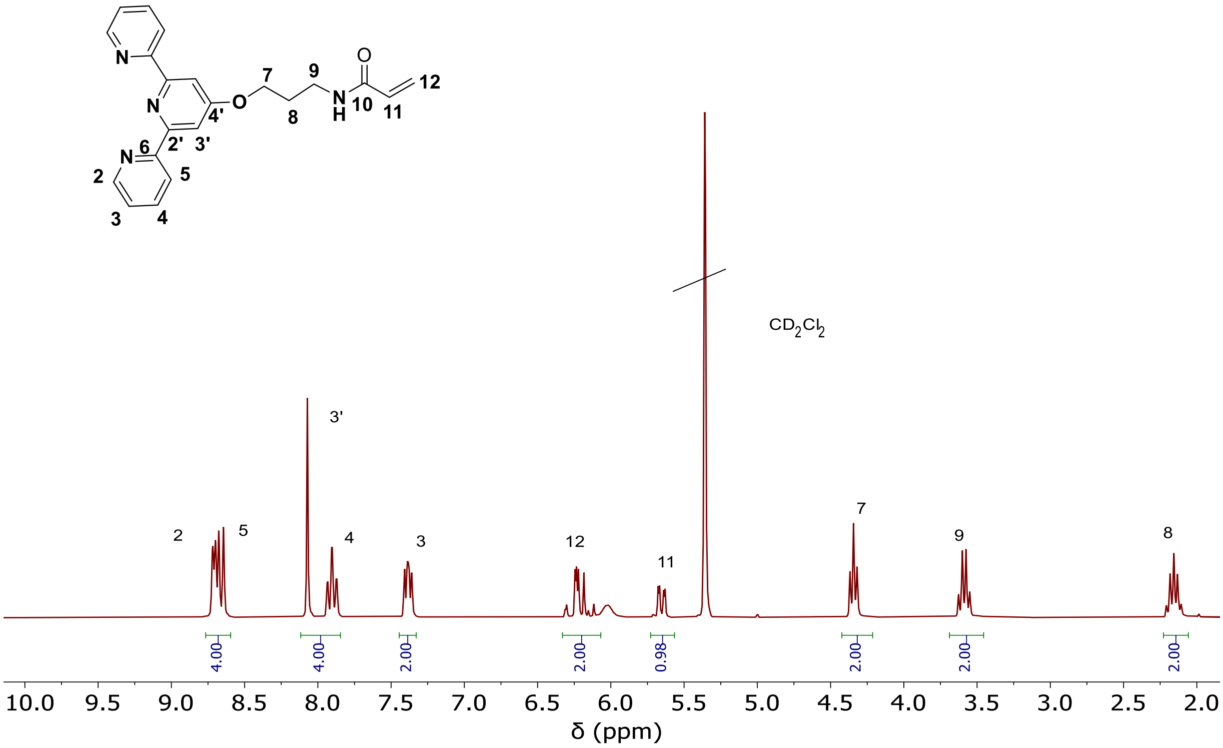

Supplementary Figure 25. ^{1}H NMR spectrum of AAm-tpy (300 MHz, CD_2Cl_2, 25°C).

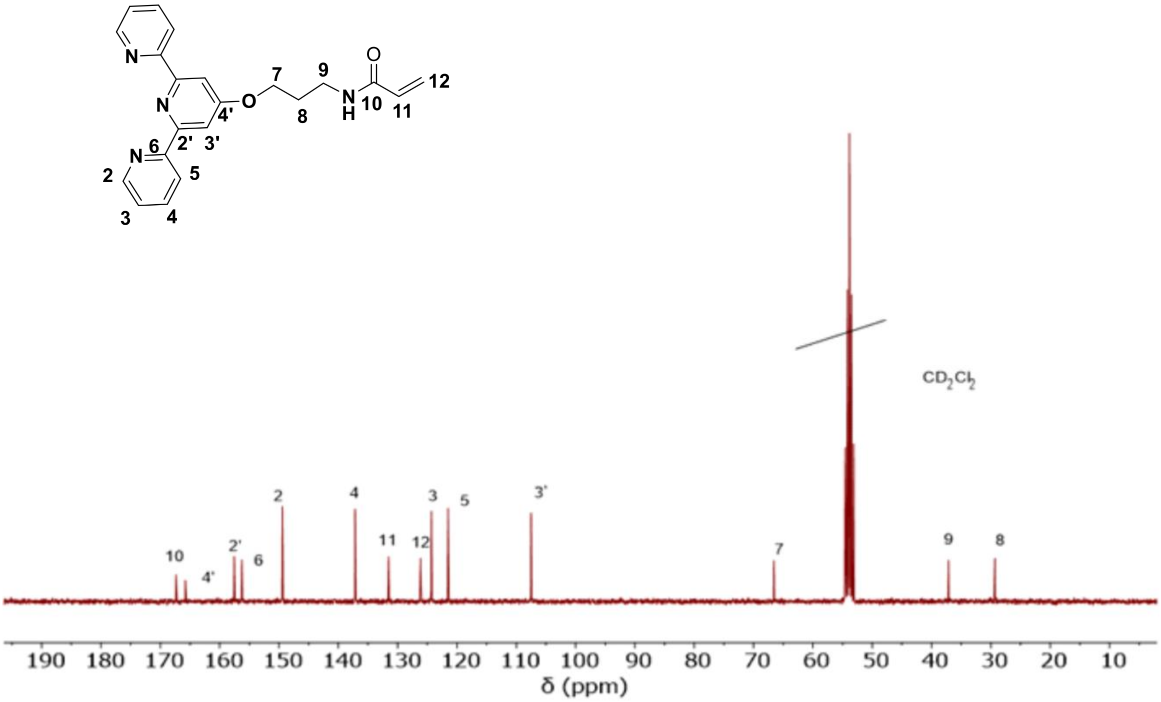

Supplementary Figure 26. ^{13}C NMR spectrum of AAm-tpy (75 MHz, CD_2Cl_2, 25°C).

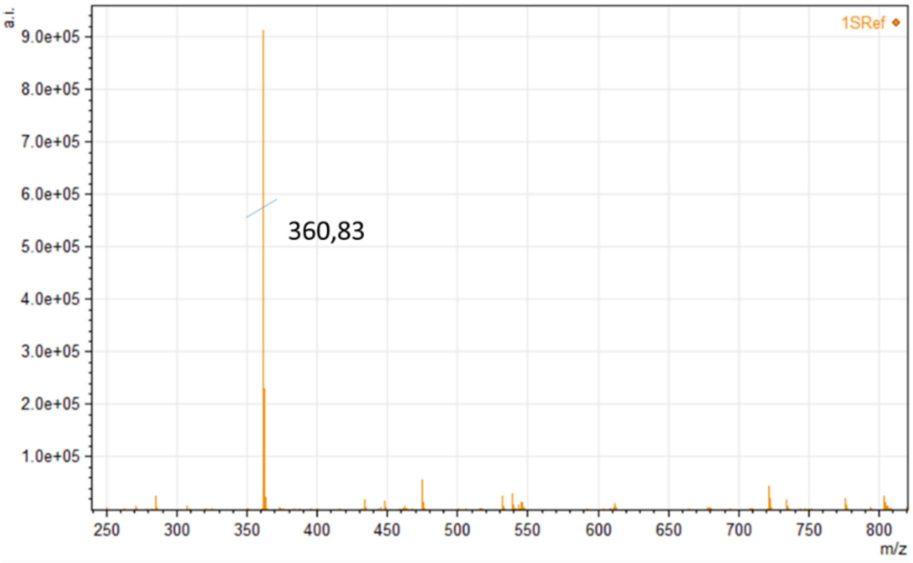

Supplementary Figure 27. MALDI-TOF-MS spectrum of AAm-tpy ($[M]^+$: found 360.83).

Synthesis of Ru(AAm-tpy)Cl_3: $RuCl_3$•xH_2O (262 mg, 1 mmol) and AAm-tpy (359.4 mg, 1 mmol) were mixed in ethanol (60 mL). The mixture was heated at reflux for 4 h. After cooling to room temperature, the mixture was filtered. The filtered brown powder was washed with ethanol and diethyl ether sequentially to obtain the product. Yield: 462 mg (94%).

Synthesis of [Ru(AAm-tpy)(bpy)(Cl)]Cl: Ru(AAm-tpy)Cl_3 (221 mg, 0.39 mmol) and 2,2'-bipyridine (bpy, 61 mg, 0.39 mmol) were mixed in a 3/1 (v/v) ethanol/H_2O mixture (30 mL). The solution was degassed for 5 min and filled with argon. Then, the reaction mixture was refluxed under argon for 1 day in the dark. After that, the mixture was filtered hot. The product was collected by evaporation under reduced pressure and purified by column chromatography with silica gel (eluent: methanol/dichloromethane = 1/8 to 1/4 (v/v)). Yield: 174 mg (65%).

^{1}H NMR of [Ru(AAm-tpy)(bpy)(Cl)]Cl (300 MHz, CD_3OD, 25°C) δ (ppm): 10.19 (d, J = 5.3 Hz, 1H, H-A1), 8.75 (d, J = 10.7 Hz, H-A4), 8.55 (d, J = 11.6 Hz, 2H, H-T4), 8.48 (d, J = 8.9 Hz, 1H, H-B4), 8.33 (s, 2H, H-T2'), 8.28 (t, J = 11.6 Hz, 1H, H-A3), 8.00-7.90 (m, 1H-A2, 2H-T3), 7.77-7.70 (m, 3H, 2H-T1, 1H-B3), 7.48 (d, J = 5.4 Hz, 1H, H-B1), 7.30 (t, J = 10.72 Hz, 2H, H-T2), 7.07 (t, J = 8.9 Hz, 1H, H-B2), 6.36-6.24 (m, 2H, H-10, H-11), 5.70 (d, J =

11.61 Hz, 1H, H-11), 4.53 (t, J = 6.25 Hz, 2H, H-6), 3.62 (t, J = 6.25 Hz, 2H, H-8), 2.24 (t, J = 8.93 Hz, 2H, H-7).

^{13}C NMR of [Ru(AAm-tpy)(bpy)(Cl)]Cl (75 MHz, CD_3OD, 25°C) δ (ppm): 168.22 (9), 167.12 (T3'), 160.68 (B5), 160.34 (T1'), 159.61 (T5), 158.39 (A5), 153.82 (B1), 153.48 (T1), 153.09 (A1), 138.16 (T3), 137.62 (B3), 136.60 (A3), 132.03 (10), 128.33 (T2), 127.99 (B2), 127.26 (A2), 126.73 (11), 124.78 (B4), 124.44 (A4), 110.26 (T2'), 68.70 (6), 37.23 (8) and 29.7 (7).

MALDI-TOF-MS of [Ru(AAm-tpy)(bpy)(Cl)]Cl: *m/z* calculated for $C_{31}H_{28}Cl_2N_6O_2Ru$ ([M-Cl]$^+$): 653.13; found 652.79.

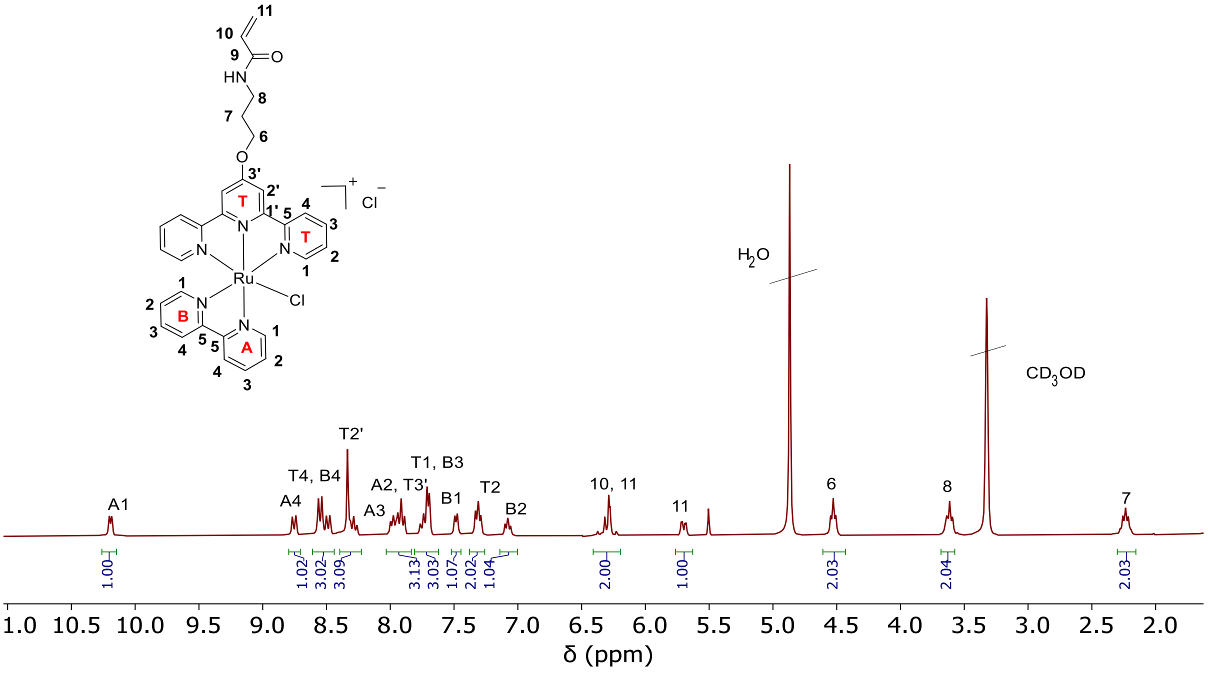

Supplementary Figure 28. ^{1}H NMR spectrum of [Ru(AAm-tpy)(bpy)Cl]Cl (300 MHz, CD_3OD, 25°C).

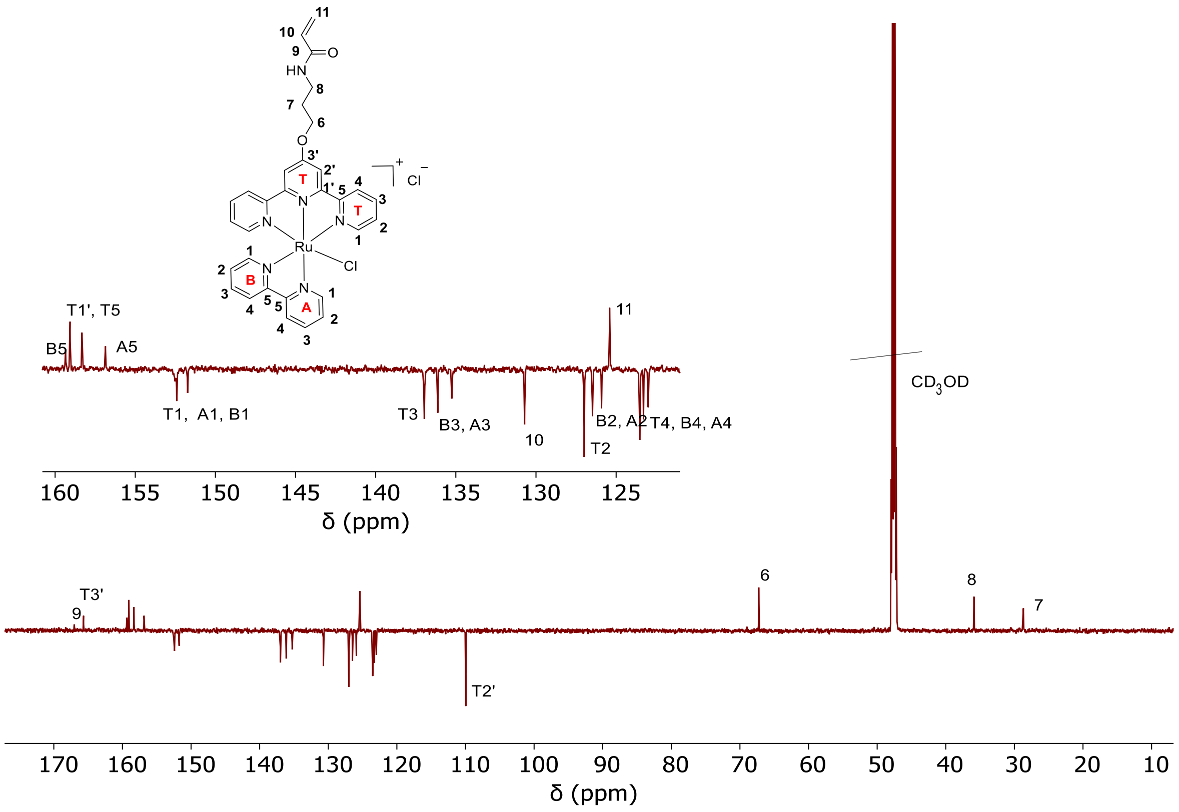

Supplementary Figure 29. ^{13}C NMR spectrum of [Ru(AAm-tpy)(bpy)Cl]Cl (75 MHz, CD_3OD, 25°C).

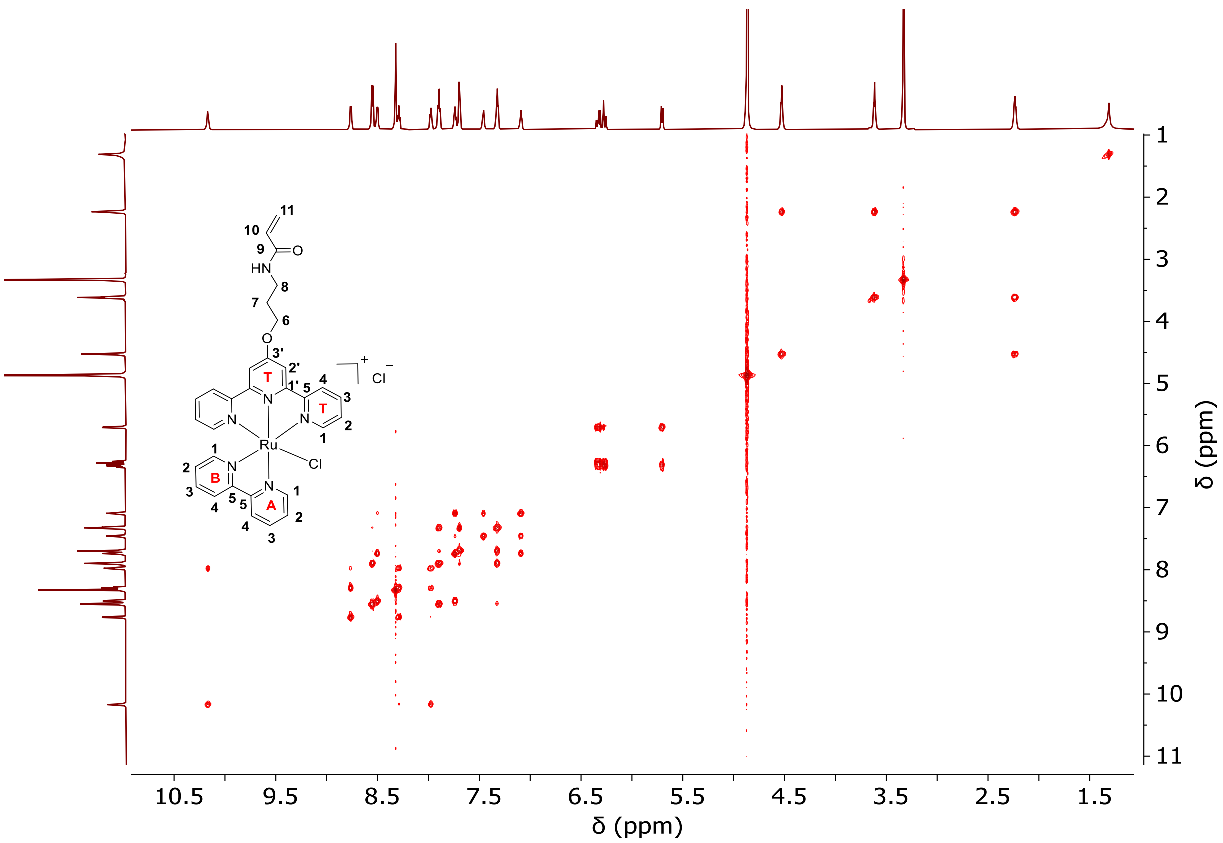

Supplementary Figure 30. H-H COSY spectrum of [Ru(AAm-tpy)(bpy)(Cl)]Cl (300 MHz, CD_3OD, 25°C).

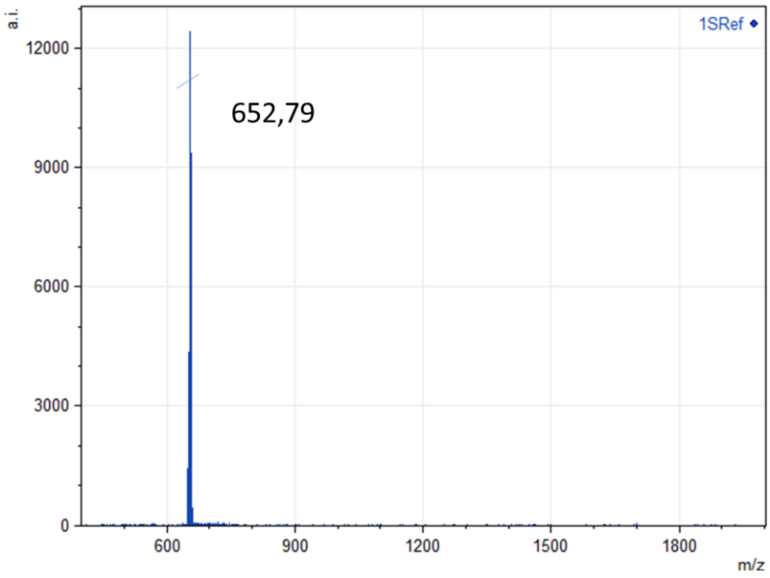

Supplementary Figure 31. MALDI-TOF-MS spectrum of [Ru(AAm-tpy)(bpy)Cl]Cl.

Synthesis of 2-(2-(2-(methylthio)ethoxy)ethoxy)ethyl acrylate

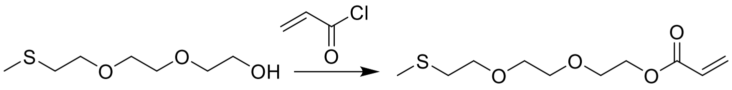

Supplementary Figure 32. Synthesis route for 2-(2-(2-(methylthio)ethoxy)ethoxy)ethyl acrylate.

Synthesis of 2-(2-(2-(methylthio)ethoxy)ethoxy)ethyl acrylate: 2-(2-(2-(Methylthio)ethoxy)ethoxy)ethan-1-ol (788.9 mg, 4.38 mmol) and triethylamine (531.85 mg, 5.26 mmol, 1.73 mL) were added to dichloromethane and stirred for 10 min at room temperature. Acryloyl chloride (475.6 mg, 5.26 mmol, 0.43 mL) was then added dropwise under argon at 0 °C for 60 min. The mixture was reacted at room temperature overnight. After that, the mixture was washed with saturated brine three times, and then extracted with dichloromethane. The organic layer was dried with magnesium sulfate and concentrated under reduced pressure. The product was a colorless oil. Yield: 513 mg (50%).

^{1}HNMR of 2-(2-(2-(methylthio)ethoxy)ethoxy)ethyl acrylate (300 MHz, $CDCl_3$, 25°C) δ (ppm): 6.42(d, J = 16 Hz, 1H, H-10), 6.10 (m, 1H, H-9), 5.76 (d, J = 12 Hz, 1H, H-10), 4.25 (t, J = 5 Hz, 2H, H-7), 3.63 (m, 8H, 2H-6,2H-5, 2H-4, 2H-3), 2.63 (t, J = 6.5 Hz, 2H, H-2), 2.07 (S, 3H, H-1).

^{13}C NMR of 2-(2-(2-(methylthio)ethoxy)ethoxy)ethyl acrylate (75 MHz, D_2O, 25°C) δ (ppm): 168.42 (8), 132.50 (9), 127.28 (10), 69.62 (7), 69.22 (6), 69.00 (5), 68.40 (4), 63.88 (3), 32.50 (2) and 14.37 (1).

ESI of 2-(2-(2-(methylthio)ethoxy)ethoxy)ethyl acrylate: *m/z* calculated for $C_{10}H_{18}O_4SNa$ ($[M+Na]^+$): 257.31, found 257.50.

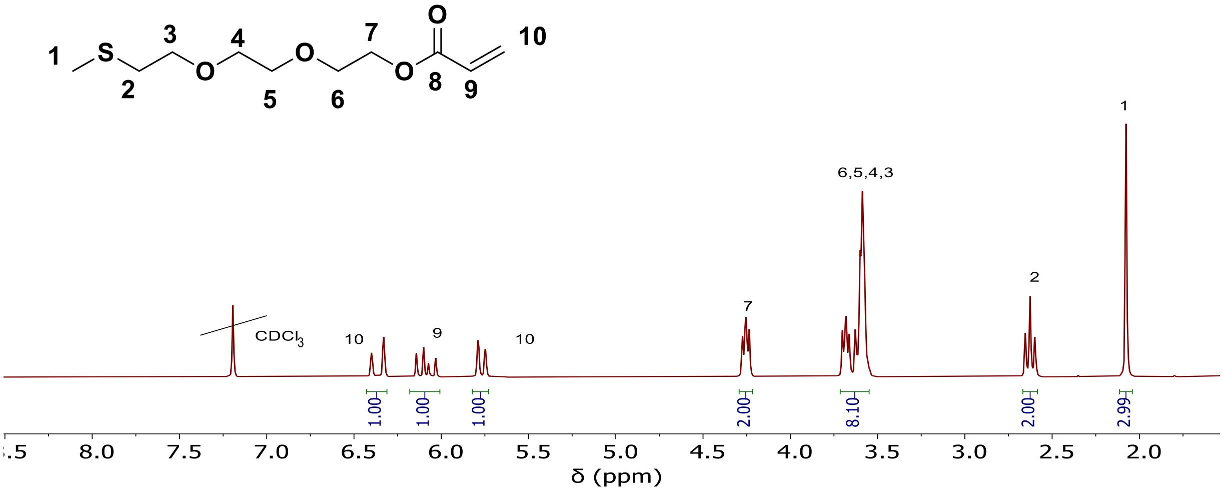

Supplementary Figure 33. ^{1}H NMR spectrum of 2-(2-(2-(methylthio)ethoxy)ethoxy)ethyl acrylate (300 MHz, $CDCl_3$, 25°C).

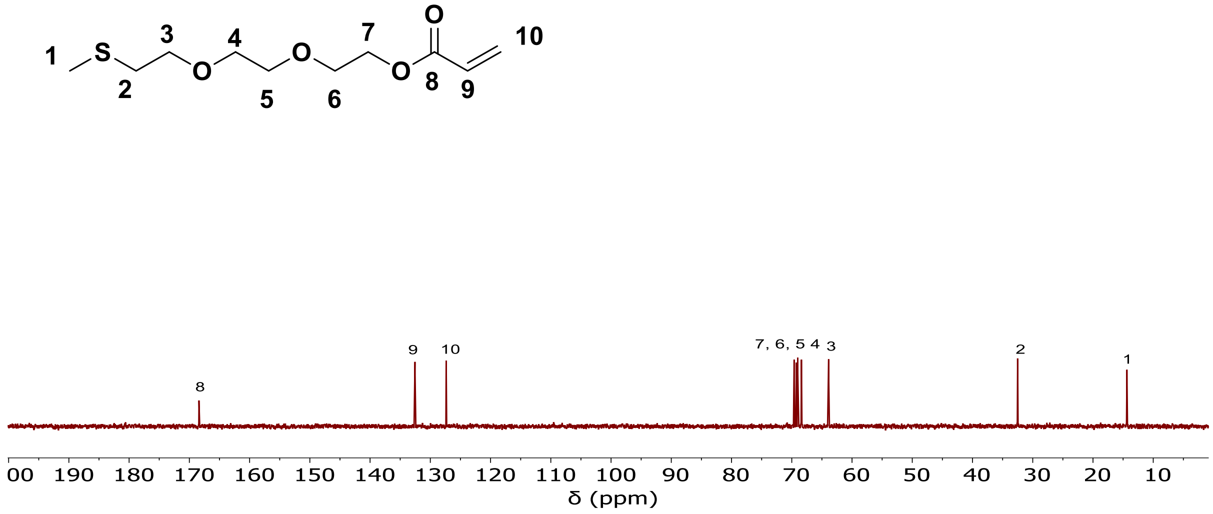

Supplementary Figure 34. ^{13}C NMR spectrum of 2-(2-(2-(methylthio)ethoxy)ethoxy)ethyl acrylate (75 MHz, D_2O, 25°C).

Synthesis of Ru-containing polymer (P-Ru)

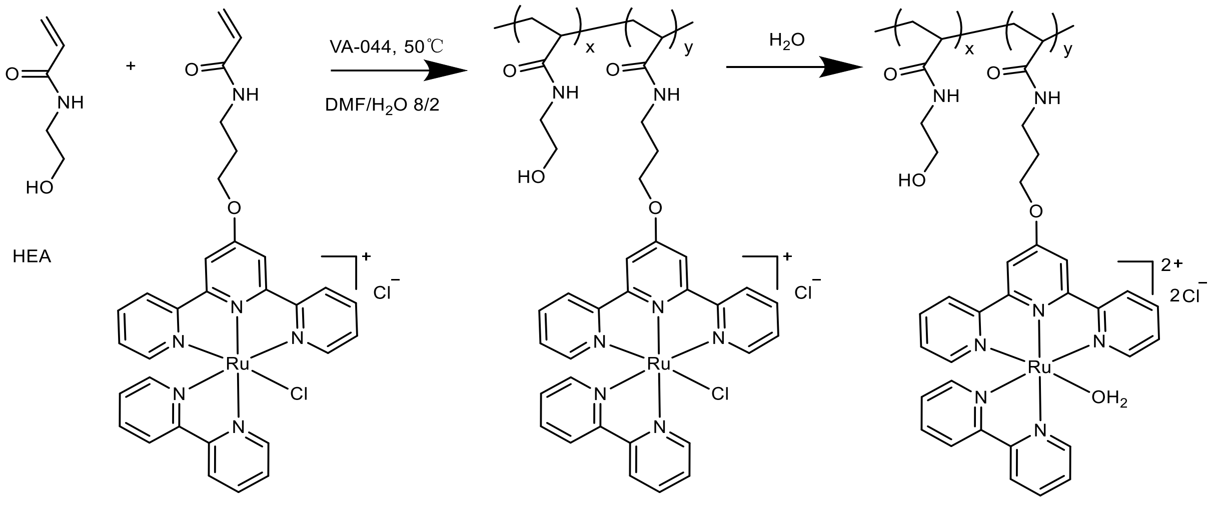

Supplementary Figure 35. Synthetic route for P-Ru (x/y = 95.3/4.7).

Synthesis of P-Ru: HEA (115.13 mg, 1 mmol) and [Ru(AAm-tpy)(bpy)Cl]Cl (34.3 mg, 0.05 mmol) were dissolved in a 3/1 (v/v) DMF/H_2O mixture (2 mL). Then, VA-044 (3.2 mg, 0.01 mmol) was added, and the resulting mixture was degassed and filled with argon for 20 min at 0 °C. The sealed flask was immersed in an oil bath at 50 °C. The polymerization was stopped after 12 h by cooling the flask to 0 °C. The polymer P-RuCl was isolated by precipitation in acetone and dried under reduced pressure. The ratio of HEA and Ru complex in the polymer was measured according to the integrals in the ^{1}H NMR spectrum (Supplementary Fig. 36). The peak at approximately 10.2 ppm corresponds to the signal of the proton connected to the Ru complex. The signal at approximately 1.0-2.5 corresponds to the signal of the proton connected to the main chain. The ratio of HEA and Ru complex parts in the polymer was 95.3/4.7, which was in accordance with the feed ratio of the monomers. P-Ru was obtained by heating P-RuCl in water for 30 min. The resulting ^{1}H NMR showed a peak at 9.50 ppm, which corresponds to the signal of the proton connected to the Ru complex part (Supplementary Fig. 37). P-Ru has a number average molecular weight of Mn = 16.4 kg/mol and a polydispersity index (PDI) of 2.91 (Supplementary Fig. 38).

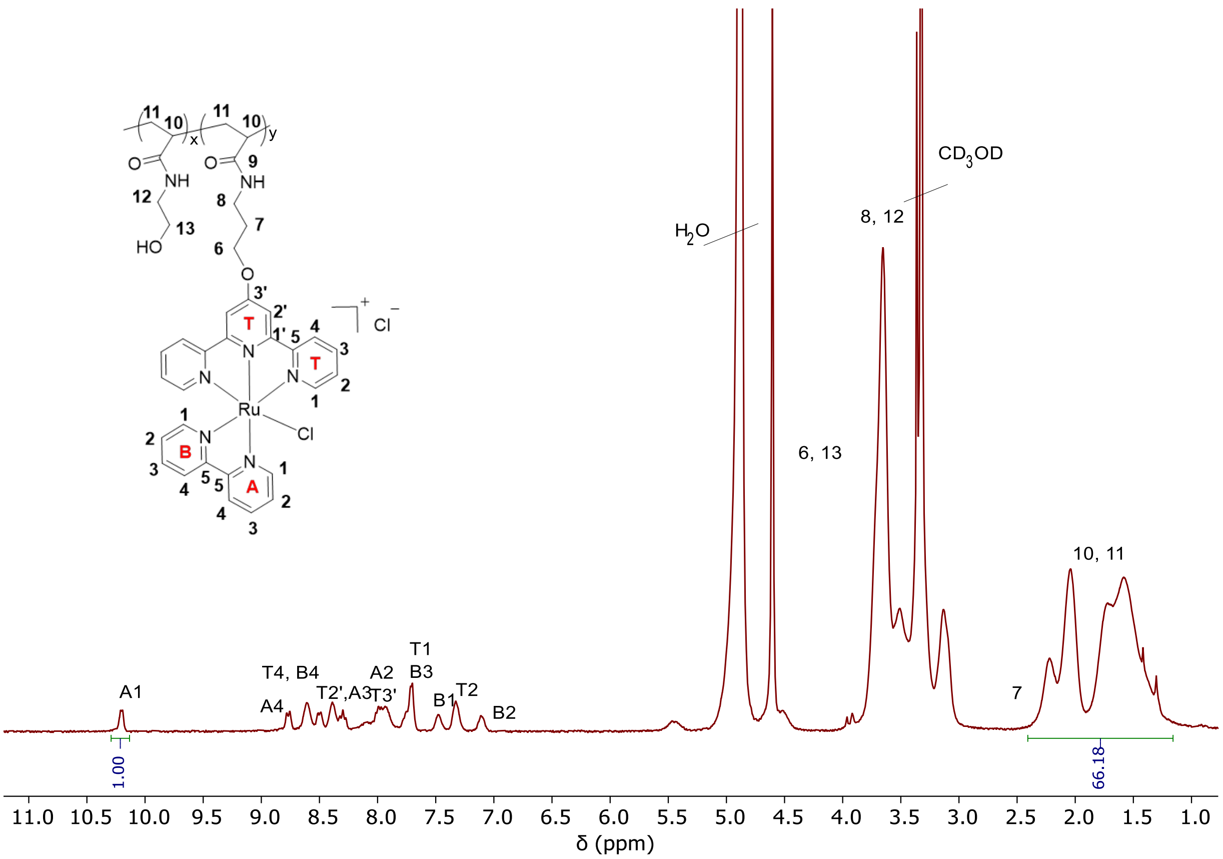

Supplementary Figure 36. ^{1}H NMR spectrum of P-RuCl (300 MHz, CD_3OD, 25°C) (x/y = 95.3/4.7).

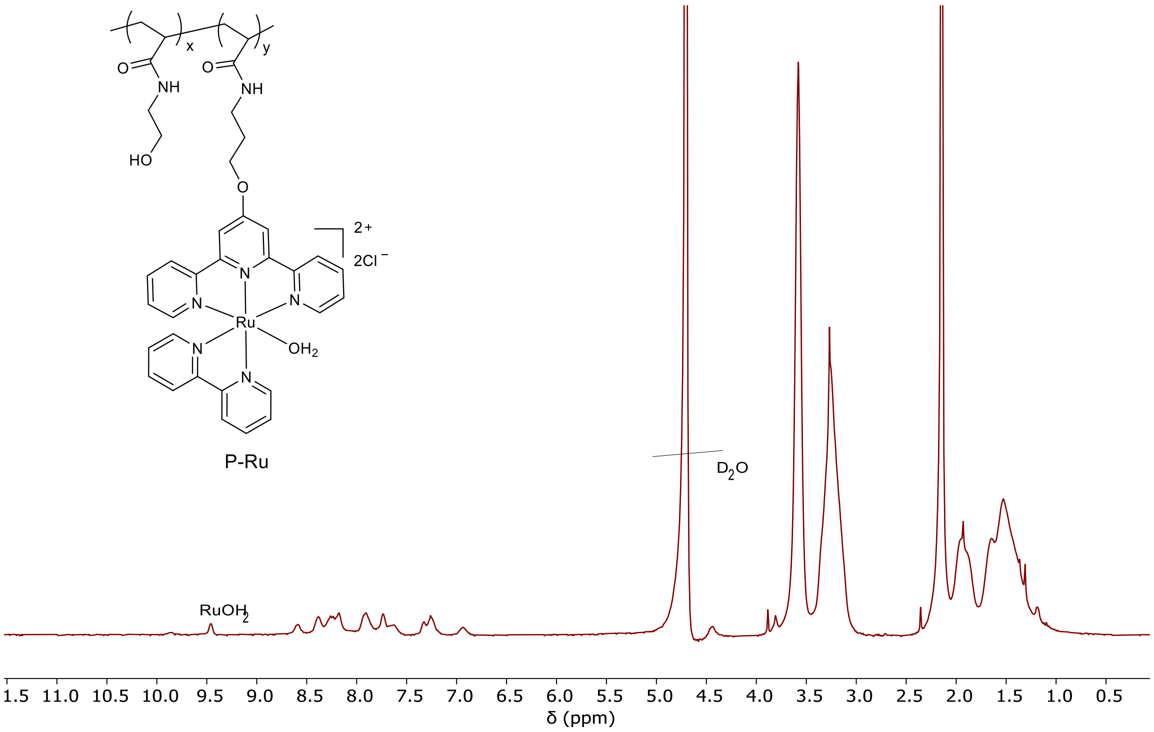

Supplementary Figure 37. ^{1}H NMR spectrum of P-Ru (300 MHz, D_2O, 25°C) (x/y = 95.3/4.7).

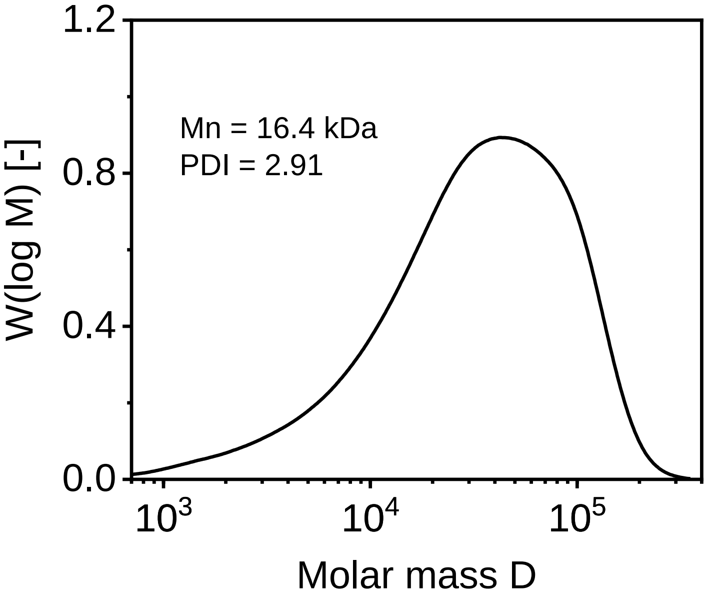

Supplementary Figure 38. GPC trace of P-Ru.

Synthesis of thioether-containing polymer (P-S)

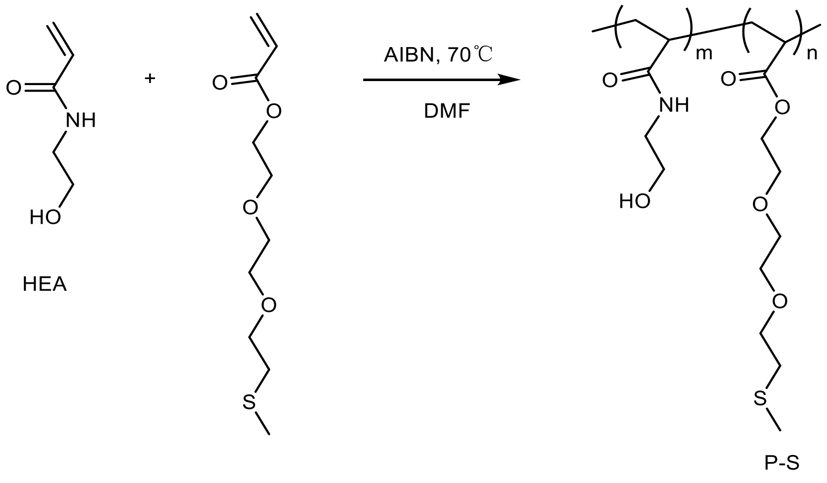

Supplementary Figure 39. Synthetic route for P-S (m/n = 79/21).

Synthesis of P-S: P-S was synthesized by free radical copolymerization of HEA and 2-(2-(2-(methylthio)ethoxy)ethoxy)ethyl acrylate at a feed mole ratio of 80:20. HEA (184 mg, 1.60 mmol) and 2-(2-(2-(methylthio)ethoxy)ethoxy)ethyl acrylate (94 mg, 0.40 mmol) were dissolved in DMF (1 mL). Then, AIBN (3.2 mg, 0.02 mmol) was added, and the resulting mixture was degassed and filled with argon for 20 min at 0 °C. The sealed flask was immersed

in an oil bath at 70 °C. The polymerization was stopped after 12 h by cooling the flask to 0 °C. Then, P-S was isolated by precipitation in acetone and dried under reduced pressure. P-S was characterized using ^{1}H NMR spectroscopy (Supplementary Fig. 40) and GPC (Supplementary Fig. 41). P-S has an M_n of 14.9 kg/mol and a PDI of 1.35. The ratio of HEA and thioether parts in P-S measured using ^{1}H NMR spectroscopy was 79/21.

We changed the ratios of the monomers and synthesized three additional thioether-containing polymers with different contents of thioether moieties (5%, 35% and 50%) using the abovementioned method. These polymers are referred to as P-S (5% SL), P-S (35% SL) and P-S (50% SL), which were used for control experiments in Supplementary Fig. 51.

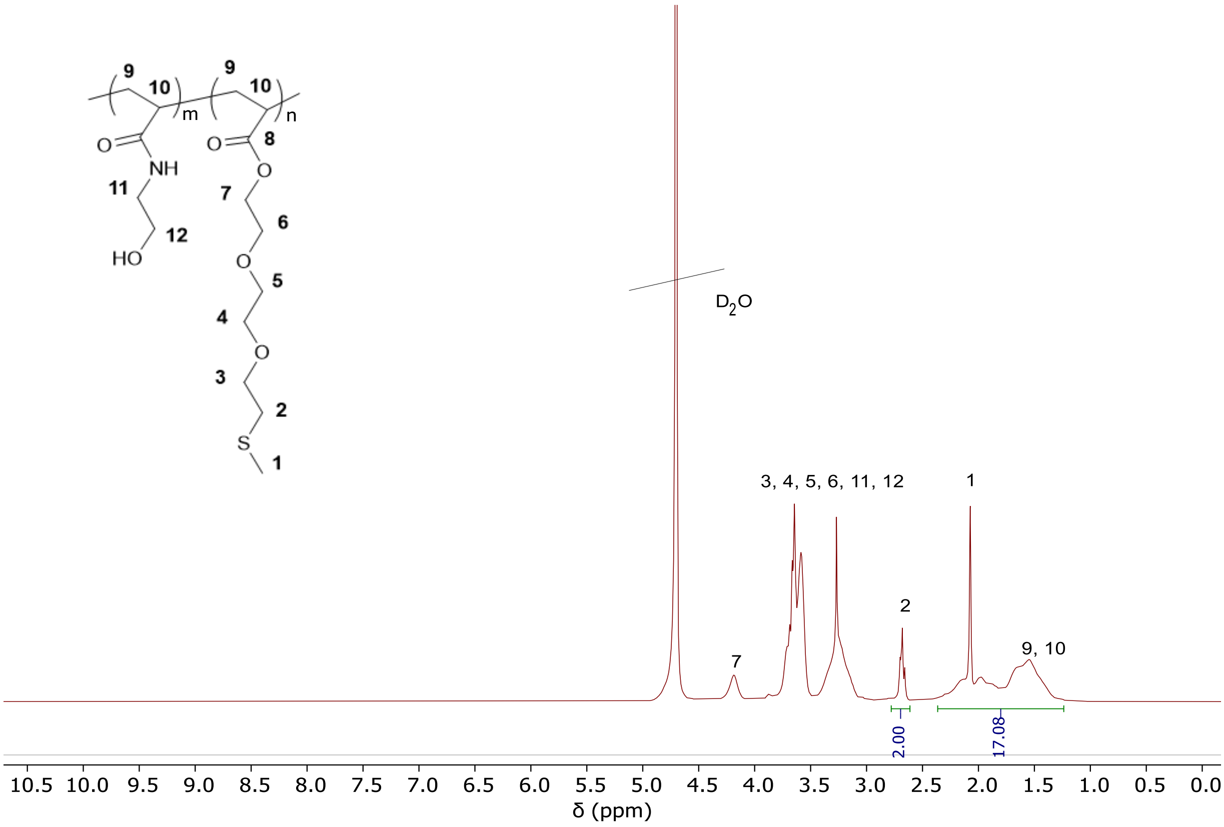

Supplementary Figure 40. ^{1}H NMR spectrum of P-S (300 MHz, D_2O, 25°C).

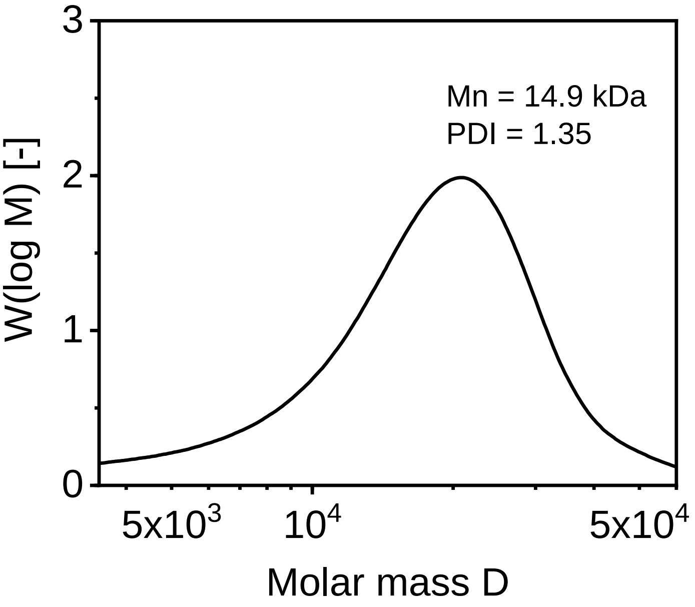

Supplementary Figure 41. GPC trace of P-S.

Synthesis of fluorescently labeled P-S

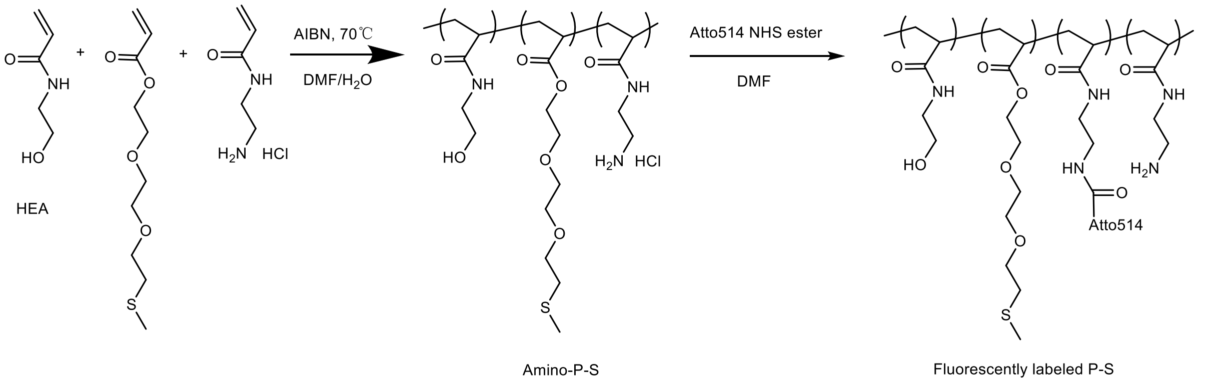

Supplementary Figure 42. Synthetic route for the fluorescently labeled P-S.

Amino-P-S was synthesized by free radical copolymerization of HEA, 2-(2-(2-(methylthio)ethoxy)ethoxy)ethyl acrylate and N-(2-aminoethyl)acrylamide hydrochloride at a feed mole ratio of 75:20:5. HEA (86 mg, 0.75 mmol), 2-(2-(2-(methylthio)ethoxy)ethoxy)ethyl acrylate (46 mg, 0.20 mmol) and N-(2-aminoethyl)acrylamide hydrochloride (8 mg, 0.05 mmol) were dissolved in a 1/1 (v/v) DMF/H_2O mixture (1 mL). Then, AIBN (1 mg, 0.006 mmol) was added, and the resulting mixture was degassed and filled with argon for 20 min at 0 °C. The

sealed flask was immersed in an oil bath at 70 °C. The polymerization was stopped after 12 h by cooling the flask to 0 °C. Then, Amino-P-S was isolated by precipitation in acetone and dried under reduced pressure. The fluorescently labeled P-S was synthesized by grafting the pendant primary amino groups in Amino-P-S with a dye that contains an NHS ester group (Atto514 NHS ester). Amino-P-S (46 mg), Atto514 NHS ester (2.3 mg) and triethyl amine (2 mg, 20 μmol) were mixed in DMF (200 μL). The mixture was stirred at room temperature for 24 h. The fluorescently labeled P-S was isolated by precipitation in acetone and dried under reduced pressure. The polymer was dialyzed for two weeks in water to remove free dyes. The fluorescently labeled P-S was characterized using ^{1}H NMR spectroscopy (Supplementary Fig. 43) and GPC with a 514 nm fluorescence detector (Supplementary Fig. 44). The fluorescently labeled P-S has an M_n of 18.1 kg/mol and a PDI of 1.20.

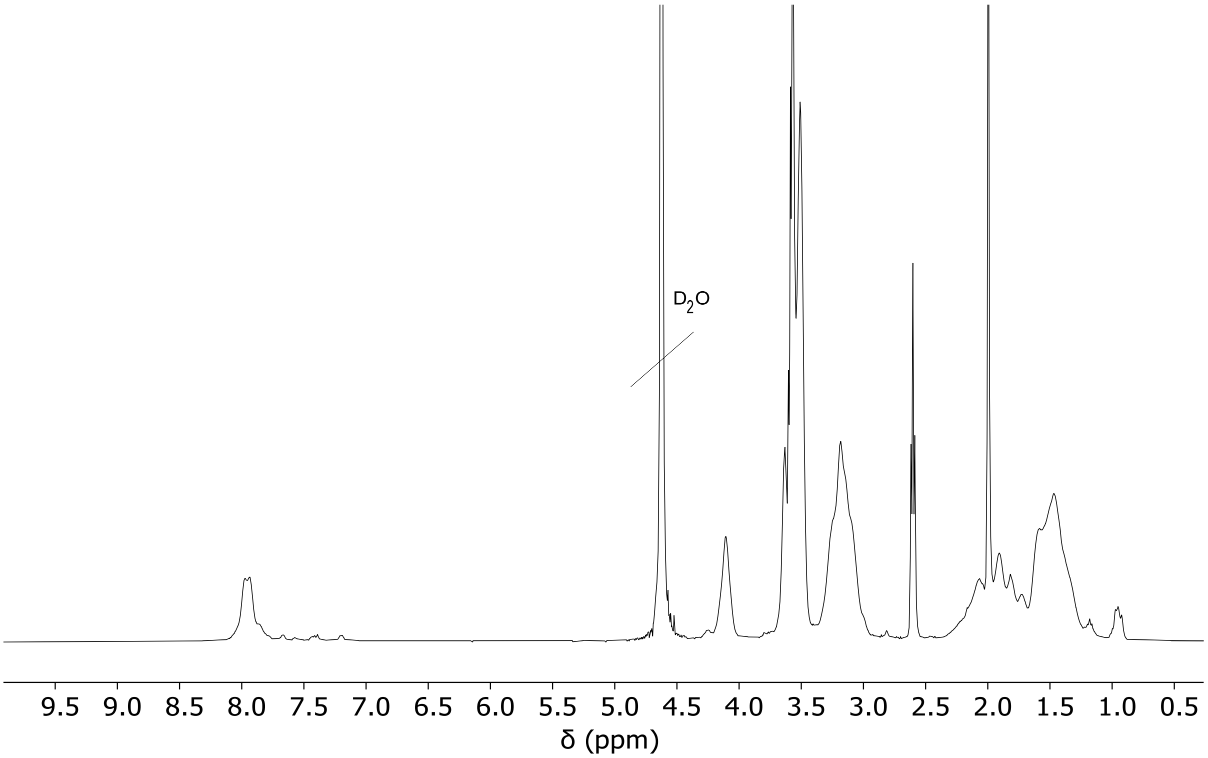

Supplementary Figure 43. ^{1}H NMR spectrum of fluorescently labeled P-S (300 MHz, D_2O, 25°C).

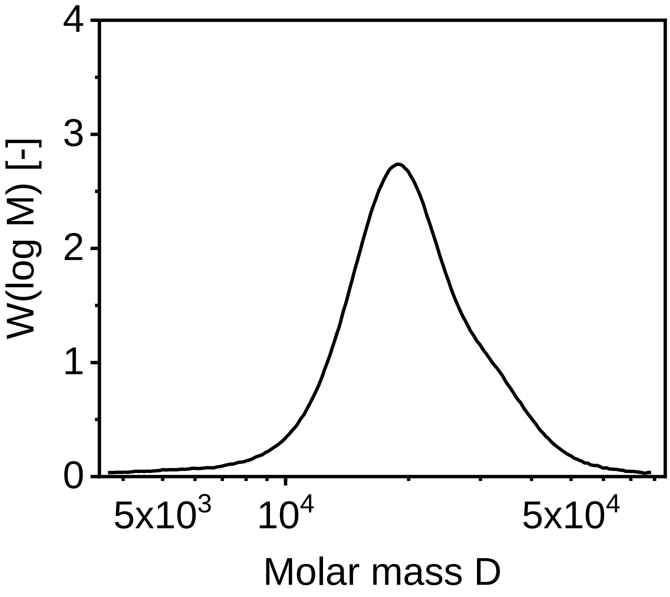

Supplementary Figure 44. GPC trace of the fluorescently labeled P-S.

Preparation of P-Ru/P-S hydrogels

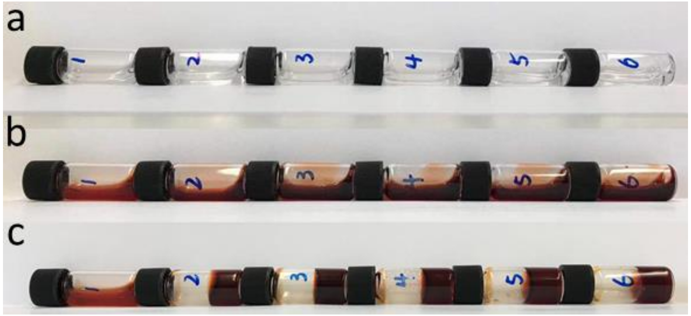

Supplementary Figure 45. Photos of P-Ru, P-S, and P-Ru/P-S in water at 70 °C. (a) P-S in water at 70 °C for 6 h. The weight percentages of P-S in water were from 1 wt% to 6 wt% (left to right). No gel was formed. (b) P-Ru in water at 70 °C for 6 h. The weight percentages of P-Ru in water were from 1 wt% to 6 wt% (left to right). No gel was formed. (c) P-Ru/P-S in water at 70 °C for 1 h. The weight percentages of P-Ru/P-S (1/1) were from 1 wt% to 6 wt% (left to right). Gels were formed in the samples with weight percentages from 2 wt% to 6 wt%.

Photocontrolled reversible coordination of P-Ru and P-S

We studied the reversible coordination of P-Ru and P-S in water using ^{1}H NMR spectroscopy (Supplementary Fig. 46). We prepared a mixture of P-Ru (10 mg) and P-S (10 mg) in 0.55 mL D_2O. Then, the mixture was immediately measured using ^{1}H NMR spectroscopy (the initial state in Supplementary Fig. 46). In the initial state, there was a doublet at 9.50 ppm that corresponds to the signal of P-Ru. When the sample was heated to 70 °C, a new doublet at 9.71 ppm appeared. The signal at 9.71 ppm increased with time, while the signal at 9.50 ppm decreased. This result indicated that Ru-thioether coordination bonds were formed upon heating. After heating for 6 h, the Ru-S coordination was complete. Then, the sample was irradiated with light (470 nm, 60 mW/cm^2, 20 min) to induce photosubstitution. After light irradiation, the P-Ru signals almost returned to the initial state. These results indicated that the Ru-S bond was cleaved by light irradiation. Upon heating the sample at 70 °C again for 6 h, the ^{1}H NMR spectrum reverted, which suggests that the Ru-S bond was formed. Thus, P-S can reversibly coordinate with P-Ru during heating/light irradiation cycles.

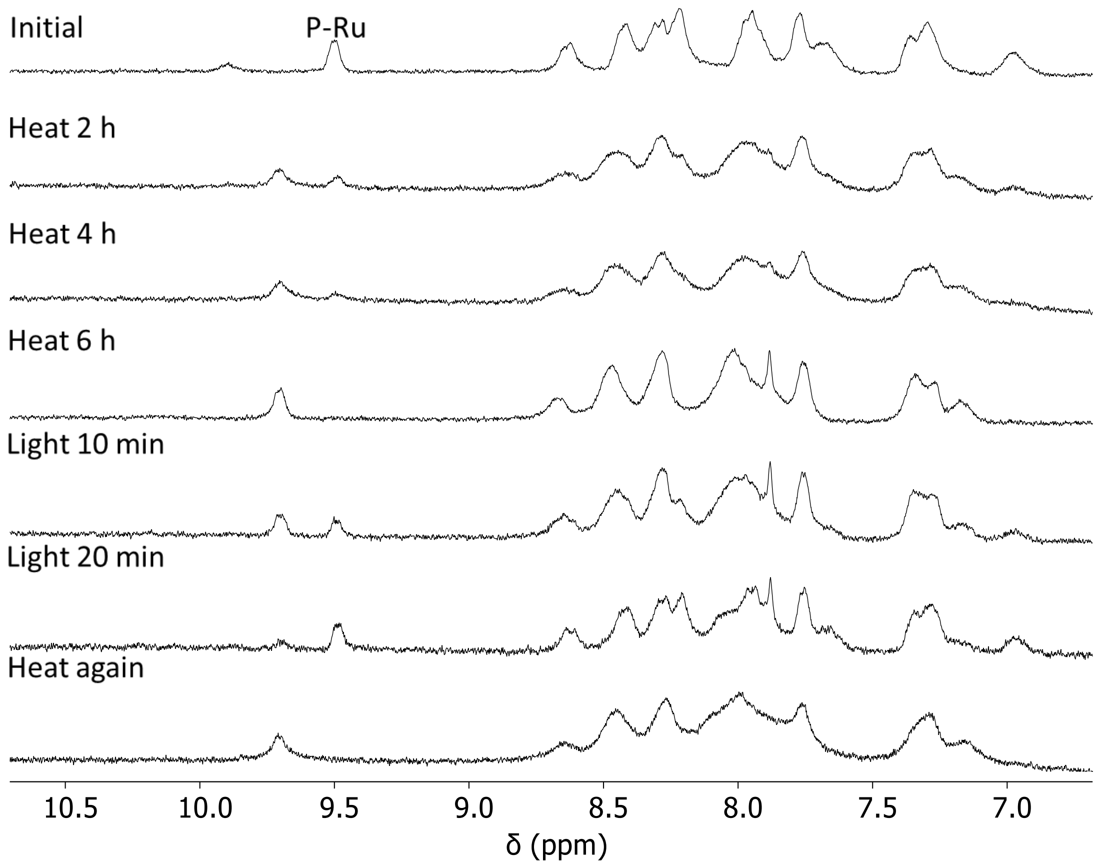

Supplementary Figure 46. ^{1}H NMR spectra showing the reversible Ru-S coordination in the mixture of P-Ru and P-S in D_2O during heating/light irradiation cycles.

Control experiments for adhesion of hydrogels

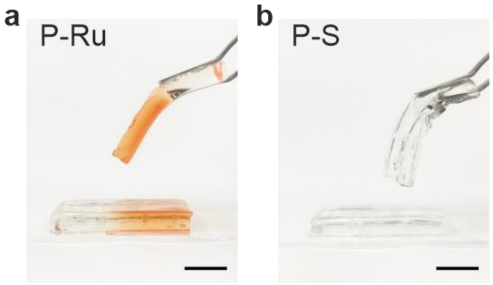

Supplementary Figure 47. P1 gels could not be glued by applying (a) P-Ru solution alone or (b) P-S solution alone to P1 gels. Scale bars: 10 mm.

Measurements of adhesion strength of glued P1 gels

Typically, a P-Ru/P-S sol (6 wt%, 5 μL/cm^2) was spread on the surfaces of two P1 gels (length: 3 cm; width: 1.5 cm; thickness: 0.2 mm). Then, a piece of P1 gel was pressed on top of the other P1 gel (overlap area: 2 cm^2). The gels were placed in a sealed container at 70 °C for 6 h. Lap shear adhesion tests were performed for the glued P1 gels using a tensile testing machine (Zwick/Roell) before and after immersing the glued P1 gels in water for 24 h.

Three tests were conducted under each condition, which showed that the adhesion measurements were reproducible (Supplementary Figs. 48 and 49). Compared with the as-prepared samples, the swollen samples showed decreased adhesion strength (Supplementary Fig. 49). The decrease in adhesion strength for swollen hydrogels is a common phenomenon.[3] Although the adhesion strength of the swollen samples decreased, the adhesive exhibited sufficient adhesion strength to glue hydrogels.

Moreover, we studied the adhesion of P1 gels glued by P-Ru/P-S with different concentrations (2, 4 and 6 wt%). The adhesion strength was adjustable using P-Ru/P-S with different concentrations (Supplementary Figs. 48 and 49).

We also studied P1 gels that were glued by P-Ru/P-S at different temperatures (50, 60, 70 and 80 °C) (Supplementary Fig. 50). As the temperature increased, the adhesion strength increased. According to these results, we infer that a higher temperature may promote the diffusion of P-Ru and P-S chains and crosslinking. Thus, the adhesion strength increased.

The content of thioether moieties on the polymer chain also affected the adhesion strength of the glued P1 gels (Supplementary Fig. 51). We prepared adhesives by mixing P-Ru with P-S (5% SL), P-S (21% SL), P-S (35% SL) and (50% SL), respectively. Their concentrations were 6 wt%. We glued P1 gels with these adhesives and found that the P1 gels glued by the adhesive with P-S (21% SL) showed the highest adhesive strength. Therefore, we used P-S (21% SL) throughout this work. The P-S (21% SL) used throughout this work is called P-S for short.

Hydrogels can be readhered, and the readhesion process is similar to that of the preparation of hydrogel assemblies using P-Ru/P-S as an adhesive, which is shown in the Methods section in the manuscript. To readhere two separate hydrogels, they were placed at room temperature for 1 h after separation, and then an aqueous solution of P-Ru/P-S (6 wt%, 10 μL/cm^2) was applied onto the hydrogel surfaces. After that, a hydrogel was placed on top of the other hydrogel, and the two hydrogels were tightly sealed together. Subsequently, the sealed hydrogels were placed inside a container at 70 °C for 6 h.

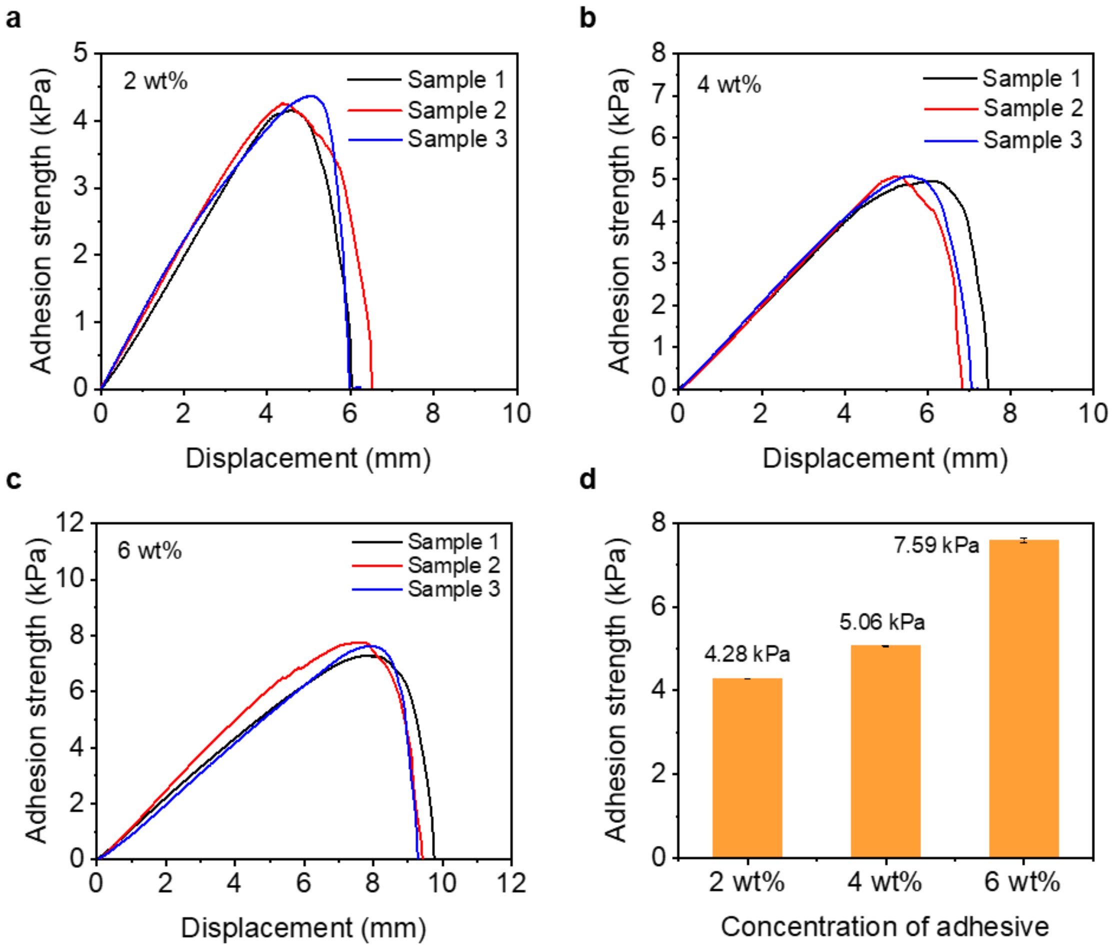

Supplementary Figure 48. (a-c) Representative adhesion strength curves of two P1 gels glued by P-Ru/P-S adhesives with different concentrations (2, 4 and 6 wt%). The adhesion measurements were conducted directly after crosslinking. Three tests were conducted under each condition, which showed that the adhesion measurements were reproducible. (d) Adhesion strength of two P1 gels glued by P-Ru/P-S adhesives with different concentrations (2, 4 and 6 wt%). Data are represented as the mean values of three measurements ± standard error.

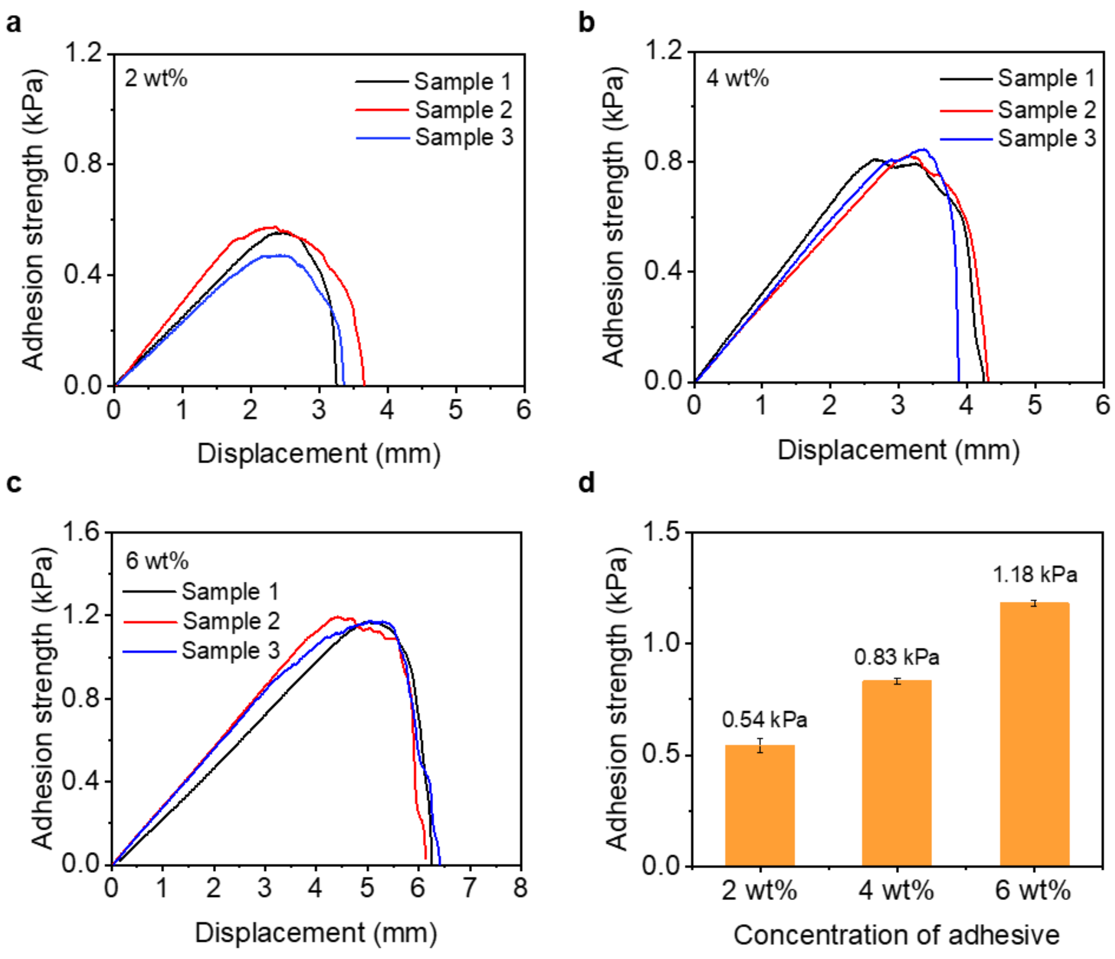

Supplementary Figure 49. (a-c) Representative adhesion strength curves of two P1 gels glued by P-Ru/P-S adhesives with different concentrations (2, 4 and 6 wt%). The adhesion measurements were conducted after the samples were immersed in water for 24 h. Three tests were conducted under each condition, which showed that the adhesion measurements were reproducible. (d) Adhesion strength of two P1 gels glued by P-Ru/P-S adhesives with different concentrations (2, 4 and 6 wt%). Data are represented as the mean values of three measurements ± standard error.

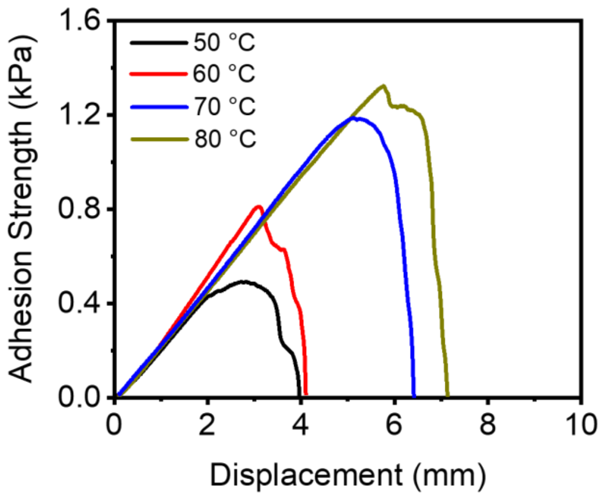

Supplementary Figure 50. Adhesion measurements of two P1 gels that were glued with 6 wt% P-Ru/P-S adhesive at different temperatures.

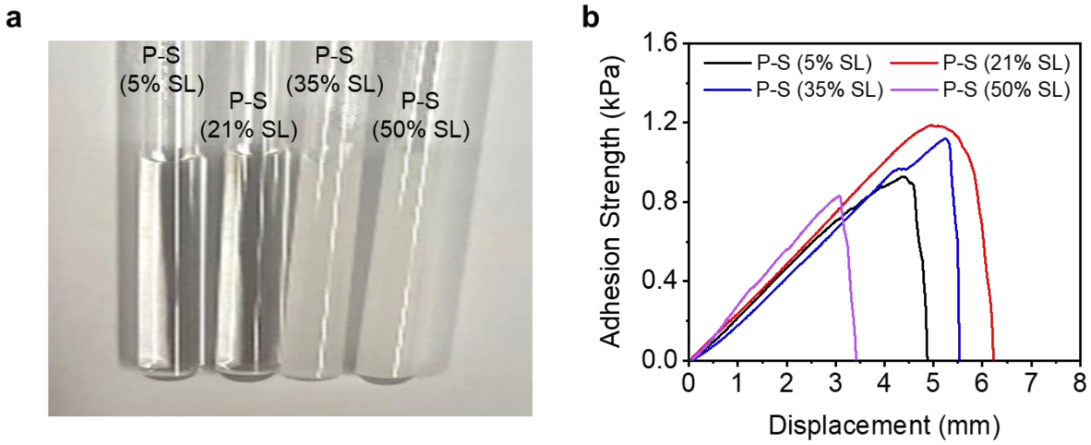

Supplementary Figure 51. (a) Photograph of the aqueous solutions of P-S (6 wt%), which had different contents of thioethers. P-S (5% SL) and P-S (21% SL) were water soluble, and transparent solutions were obtained. In contrast, P-S (35% SL) and P-S (50% SL) were not fully soluble in water, and cloudy dispersions were obtained. (b) Adhesion measurements of two P1 gels that were glued with 6 wt% P-Ru/P-S (5% SL), P-Ru/P-S (21% SL), P-Ru/P-S (35% SL), and P-Ru/P-S (50% SL). We infer that the adhesion strength of P-Ru/P-S (5% SL) was weaker than that of P-Ru/P-S (21% SL) because P-Ru/P-S (5% SL) formed fewer crosslinks. We infer that the adhesion strengths of P-Ru/P-S (35% SL) and P-Ru/P-S (50% SL) were weaker than

that of P-Ru/P-S (21% SL) because of their worse water solubility. P-Ru/P-S (21% SL) showed the highest adhesion, which is the P-Ru/P-S adhesive used throughout this work.

SEM images of P-Ru/P-S gel and P1 gel

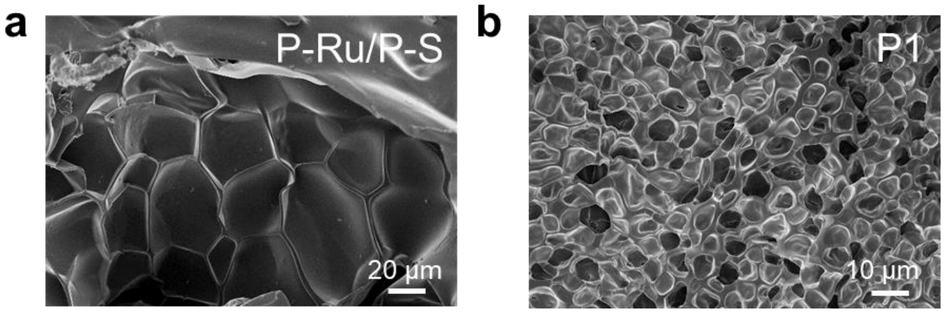

Supplementary Figure 52. SEM images of pure P-Ru/P-S gel (a) and pure P1 gel (b). The SEM images confirmed that the average pore diameter of the P-Ru/P-S gel was approximately three times larger than that of the P1 gel. P1 gel was prepared using a precursor with a concentration of 118.5 mg/mL, and P-Ru/P-S gel was prepared using a precursor with a concentration of 60 mg/mL. A higher concentration may lead to a more densely packed gel structure. Additionally, the chemical structures of the P1 and P-Ru/P-S gels are different, which may also affect their morphology.

EDS results of P-Ru/P-S-glued P1 gel

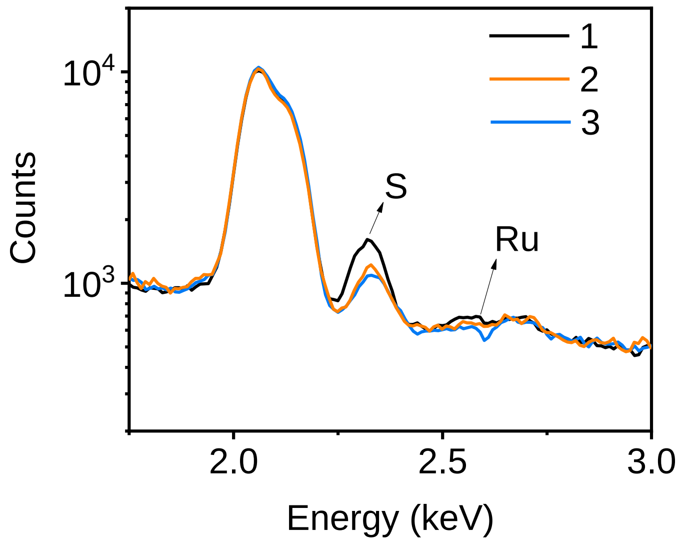

Supplementary Figure 53. EDS data of regions 1, 2 and 3 in Fig. 3a in the manuscript. The signal of Ru was detected in these regions. However, the contents of Ru were lower than 0.1%, which is the lower limit for quantitative analysis. The contents of Ru were lower than those of S because the ratio of Ru/S in P-Ru/P-S was 4.7/21.

Raman spectra and imaging

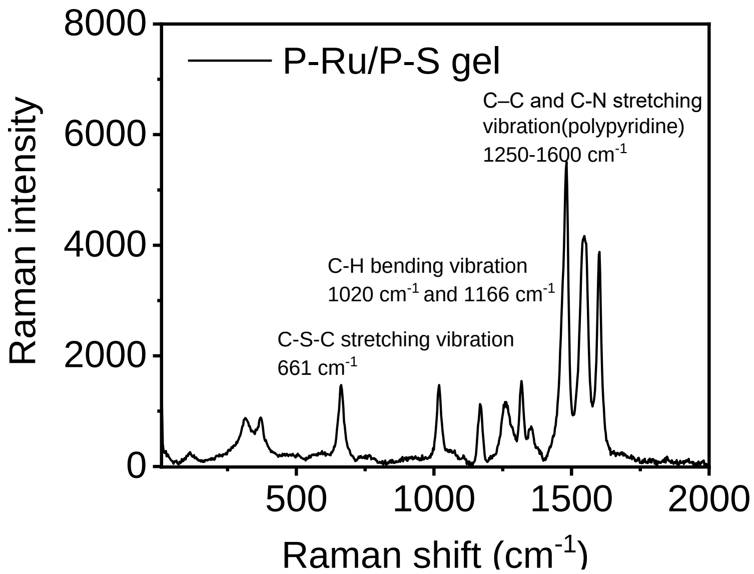

Supplementary Figure 54. Raman spectrum of a P-Ru/P-S gel.

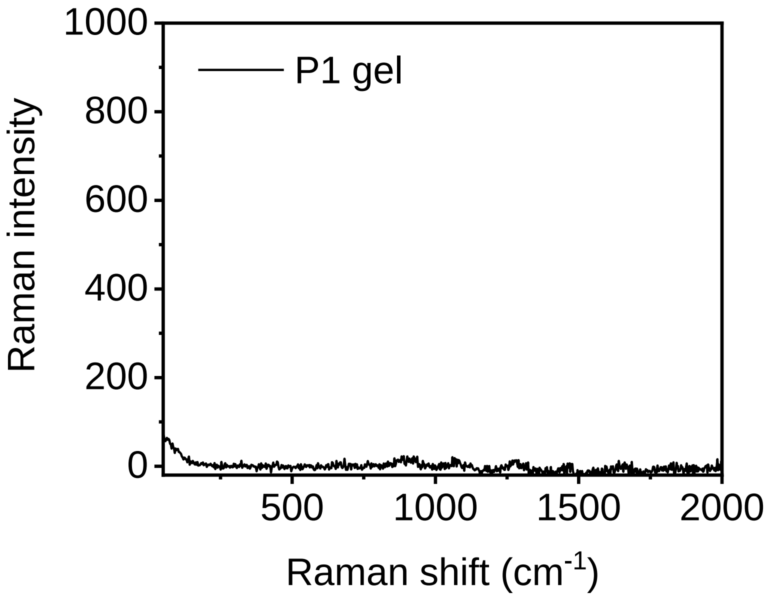

Supplementary Figure 55. Raman spectrum of a P1 gel.

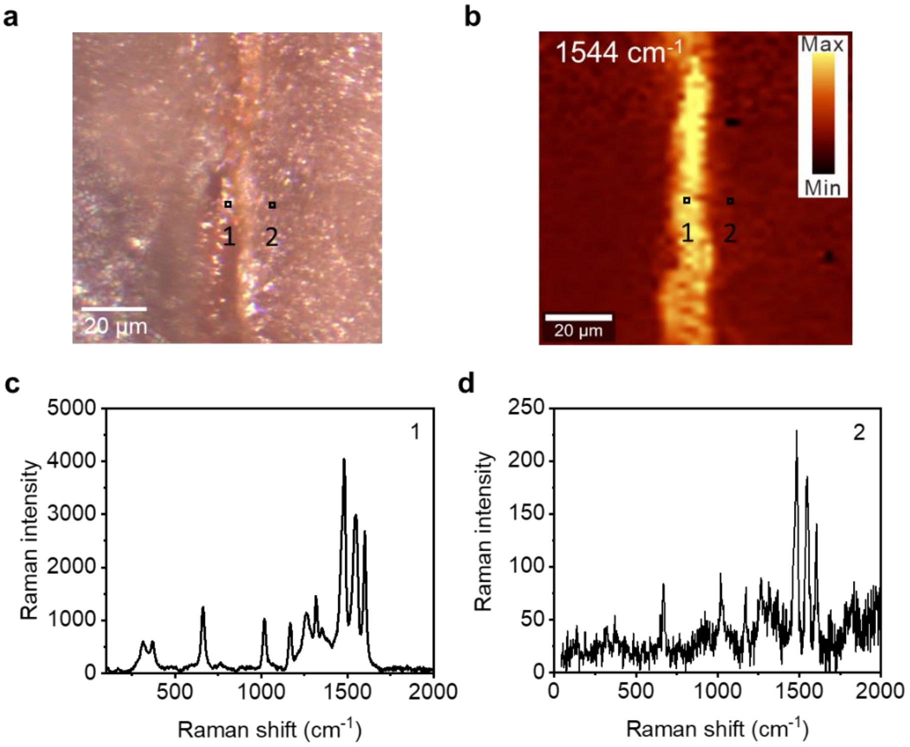

Supplementary Figure 56. (a) Optical microscopy image and (b) Raman map at 1544 cm^{-1} of the two P1 gels glued by a P-Ru/P-S adhesive. Raman spectra at (c) Point 1 and (d) Point 2 of the optical microscopy image and Raman map. Point 1 is in a P-Ru/P-S-rich region and Point 2 is in a P1-rich region. The signals of P-Ru/P-S were observed in the P-rich region, which suggests that P-Ru/P-S penetrated into P1.

Computer simulations

A simulation unit cell was built to model the penetration of P-Ru and P-S chains into P1. The simulation unit cell contains a crosslinked P1, 4 P-Ru chains, 6 P-S chains, and 9000 H_2O molecules. The crosslinked P1 was constructed using 10 segments of P1 chains, which were crosslinked by the covalent crosslinker (N,N'-methylenebisacrylamide). A mixture of the crosslinked P1 and 6000 water molecules was at the bottom of the simulation unit cell; a mixture of 4 P-Ru chains, 6 P-S chains and 3000 water molecules was at the top of the simulation unit cell. Periodic boundary conditions were applied to the simulation system. The final model contains 32075 atoms, including 3715 atoms for the crosslinked P1, 640 atoms for P-Ru chains, 720 atoms for P-S chains, and 27000 atoms for the water molecules. The dimensions of the model were 60.00 × 60.00 × 96.45 $Å^3$. The molecular models of the segment of P1, crosslinked P1, P-Ru, P-S, and water are shown in Supplementary Fig. 57. P1 segments are bonded together at the boundary, which shows that the crosslinked P1 could be considered an infinite 3D polymer network because a periodic boundary is adopted. The system was minimized to a relaxed initial configuration using the conjugate gradient minimization algorithm with the following parameters:

```
min_style    cg
minimize     1.0e-5 1.0e-5 5000 50000.
```

It took 264 steps to reach the relaxed configuration. The initial configuration of the system is shown in Supplementary Fig. 58.

To observe the penetration of P-Ru and P-S into P1, a 50.0 ns NPT molecular dynamics simulation was performed at 343 K and 1.0 atm. During the MD simulations, the system spontaneously evolved towards a more thermodynamically stable state. The temperature and

pressure for the simulations are in line with the experiments, which were performed at 70 ℃ and 1.0 atm.

LAMMPS was used to perform the molecular simulations[4]. All the force field parameters for P1, P-S, and H_2O were taken from the PCFF-INTERFACE force field[5]. The force field for Ru, which is absent in the PCFF-INTERFACE force field, was obtained by the following method. First, a cluster model of the Ru moiety, which is related to P-Ru, was built (Supplementary Fig. 59). The Ru moiety has two positive charges. The geometry of the Ru moiety was optimized by DFT calculation, which was performed by Gaussian 16[6] using the M06L functional[7] and Lanl2DZ basis set for Ru and the 6-31G* basis set for other atoms. Based on the obtained quantum mechanical data, the parameters of harmonic bonds and angles of Ru were generated with the ForceFit program[8] (Supplementary Table 1). We used the parameters of dihedral terms and Lennard-Jones interactions that were developed by Brandt et al.[9] and derived from the UFF force field[10].

The temperature and pressure were controlled using the Nosé-Hoover thermostat and barostat [11]. The equations of motions were integrated using the velocity-Verlet method with a 1.0 fs time step. The cutoffs for all the nonbonded interactions are 12 Å, and the PPPM method was used to account for the long-range electrostatic interactions[12]. The non-bonded van der Waals interactions with the same atom types are determined based on the Lennard-Jones potential. The potential well depth (ε) and equilibrium distance (σ) between different atom types are calculated with a sixth power mixing rule. The atomic coordinates were collected every 0.5 ns for postanalysis.

The interaction energies of P-Ru and P-S with P1 were calculated using the following equation:

$$E_{Inter} = E_{total} - (E_{P\text{-}Ru/P\text{-}S} + E_{P1}) \qquad \text{Equation (2)}$$

where E_{Inter} represents the interaction energy, and E_{total}, $E_{P\text{-}Ru/P\text{-}S}$ and E_{P1} are the energies of the complex, P-Ru/P-S, and P1, respectively. The interaction energies at different time periods are shown in Supplementary Table 2.

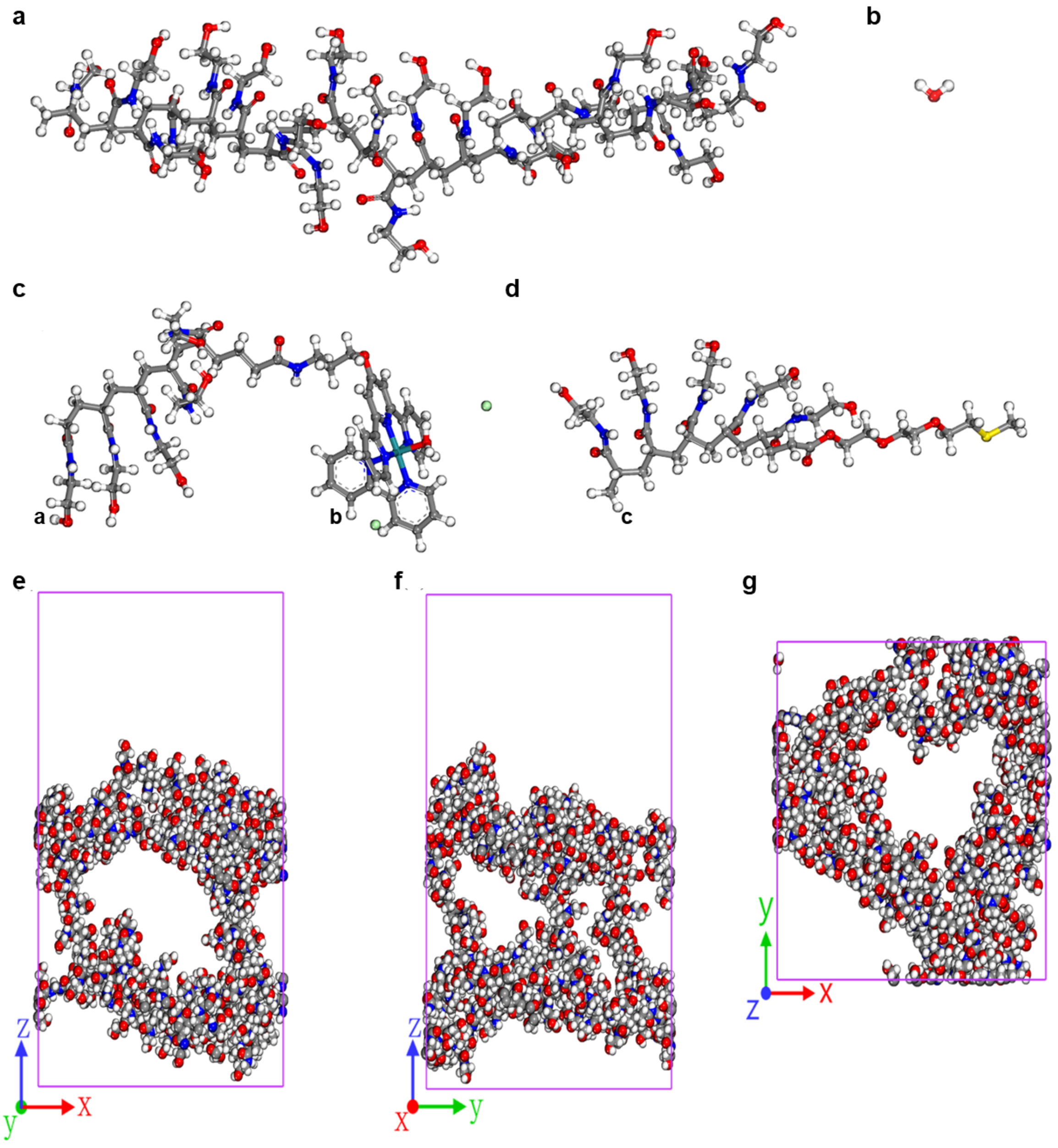

Supplementary Figure 57. Structures of (a) a segment of P1, (b) H_2O, (c) P-Ru, (d) P-S and (e-g) front view, side view, and top view of a crosslinked P1. Color codes for different atoms: white, H; red, O; gray, C; blue, N; yellow, S; cyan, Ru; pale green, Cl.

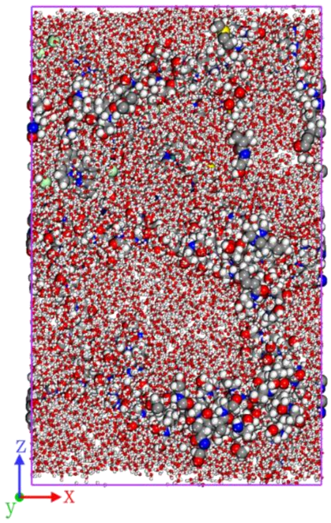

Supplementary Figure 58. The initial configurations of P1, P-Ru, P-S and H_2O. Color codes for different atoms: white, H; red, O; gray, C; blue, N; yellow, S; cyan, Ru; pale green, Cl.

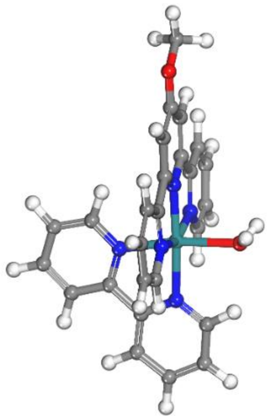

Supplementary Figure 59. The model of the Ru moiety that is related to P-Ru. Color codes for different atoms: white, H; red, O; gray, C; blue, N; cyan, Ru.

Supplementary Table 1. Harmonic bond and angle parameters

Bond type	K (kcal/mol/Å^2)	Equilibrium bond distance (Å)	Angle type	K (kcal/mol/rad^2)	Equilibrium angle (degrees)
Ru–N	81.8	2.082	C–N–Ru	76.9	120.5
Ru–O	48.7	2.251	H–O–Ru	41.5	115.6
			N–Ru–N	87.5	112.2
			N–Ru–O	65.4	106.7

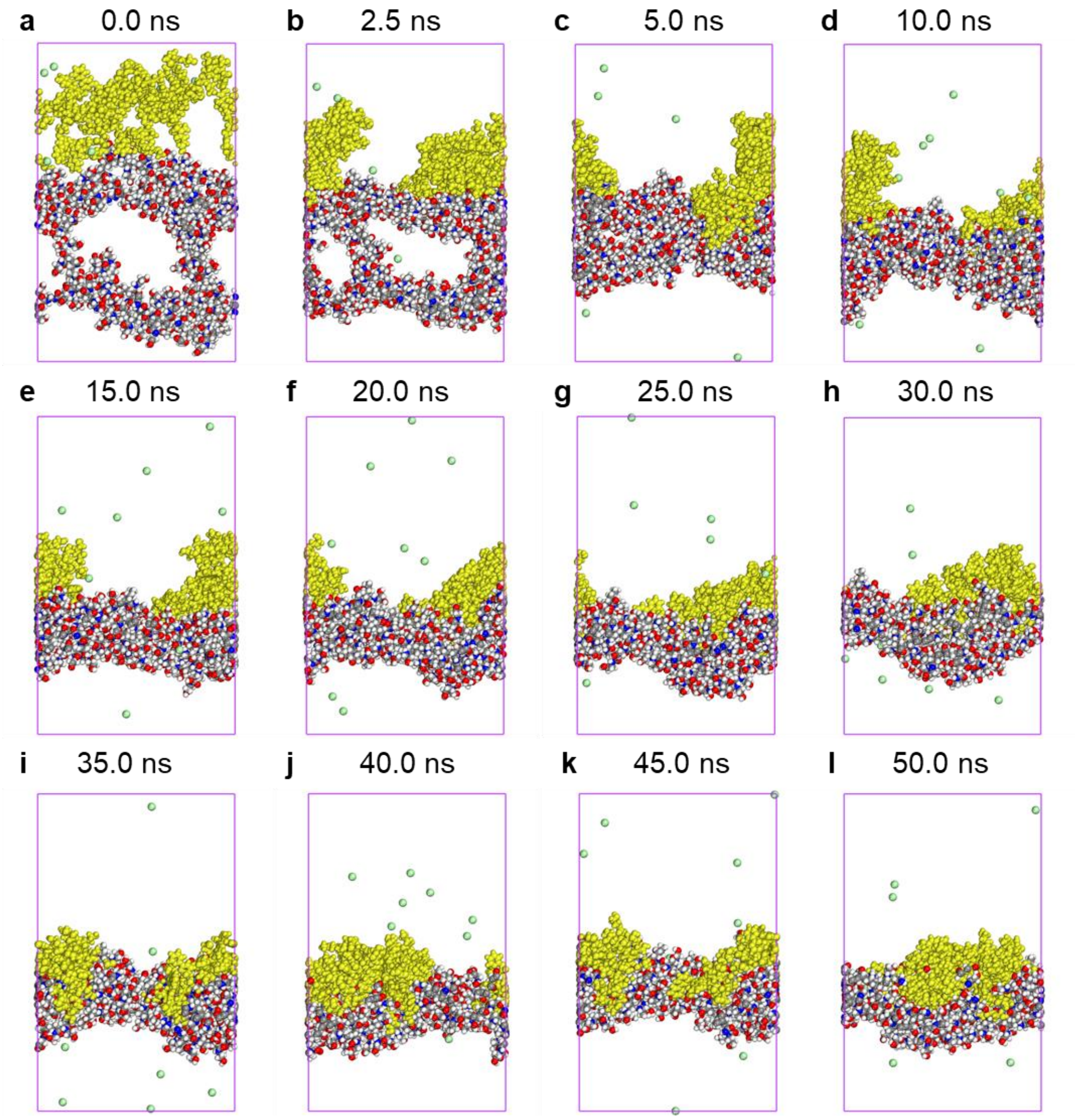

Supplementary Figure 60. Snapshots of the penetration of P-Ru and P-S into P1. For clarity, H_2O molecules are omitted, and P-Ru and P-S chains (except for Cl^-) are highlighted with a yellow color. The configurations of the system at 0.0 ns (a), 2.5 ns (b), 5.0 ns (c), 10.0 ns (d), 15.0 ns (e), 20.0 ns (f), 25.0 ns (g), 30.0 ns (h), 35.0 ns (i), 40.0 ns (j), 45.0 ns (k) and 50.0 ns

(l) of MD simulation at 343 K are shown. Color codes for different atoms: white, H; red, O; gray, C; blue, N; Cl, cyan.

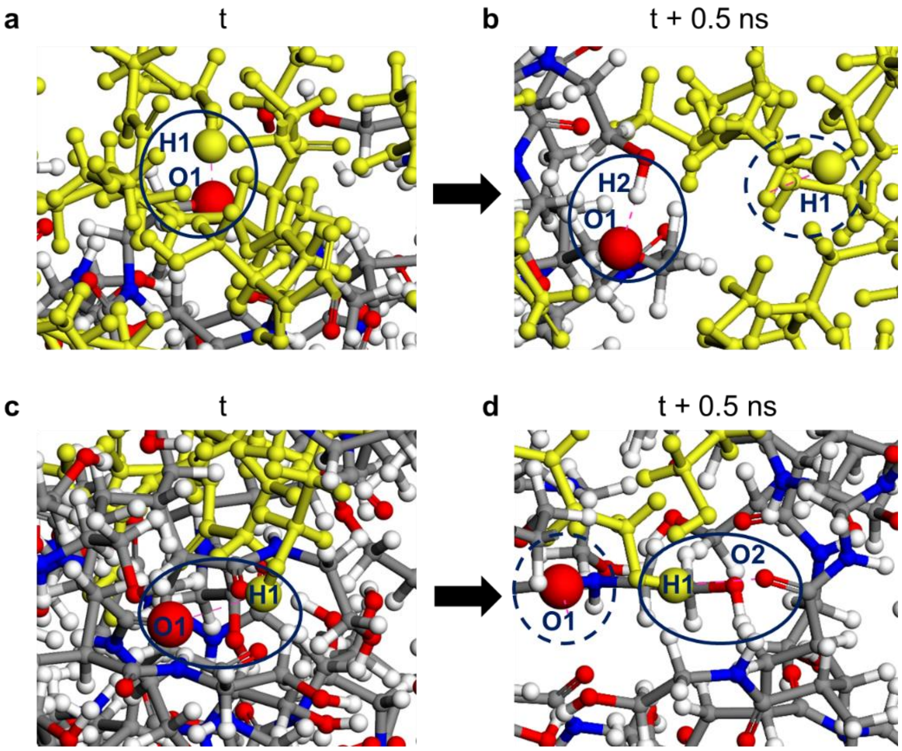

Supplementary Figure 61. The formation and reconfiguration of hydrogen bonds during the penetration of P-Ru and P-S into P1. For clarity, H_2O molecules are omitted, and P-Ru and P-S chains (except for Cl^-) are highlighted with a yellow color. (a) A typical hydrogen bond between an oxygen (O1) of P1 and a hydrogen (H1) of P-S. (b) After 0.5 ns, the hydrogen bond between O1 and H1 dissociated, and a new hydrogen bond between H2 and O1 formed. (c) A typical hydrogen bond between an oxygen (O1) of P1 and a hydrogen (H1) of P-Ru. (d) After 0.5 ns, the hydrogen bond between O1 and H1 dissociated and a new hydrogen bond between H1 and O2 formed. Color codes for different atoms: white, H; red, O; gray, C; blue, N; Cl, cyan.

The computer simulation showed that the hydrogen bonds between P1 and P-Ru/P-S are dynamic, and can form, dissociate and reconfigure. Thus, P-Ru and P-S can diffuse in P1 via reconfiguration of hydrogen bonding.

Supplementary Table 2. Interaction energies of P-Ru and P-S with P1 at different time periods in Supplementary Fig. 60.

Time (ns)	Interaction energy (kcal/mol)
0.0	−0.37
5.0	−288.17
10.0	−434.22
15.0	−396.47
20.0	−439.23
25.0	−420.51
30.0	−500.09
35.0	−537.22
40.0	−528.60
45.0	−509.18
50.0	− 628.59

The data revealed that the interaction energy increased as P-Ru and P-S penetrated into P1.

Adhesion strength of different substrates glued by P-Ru/P-S adhesives

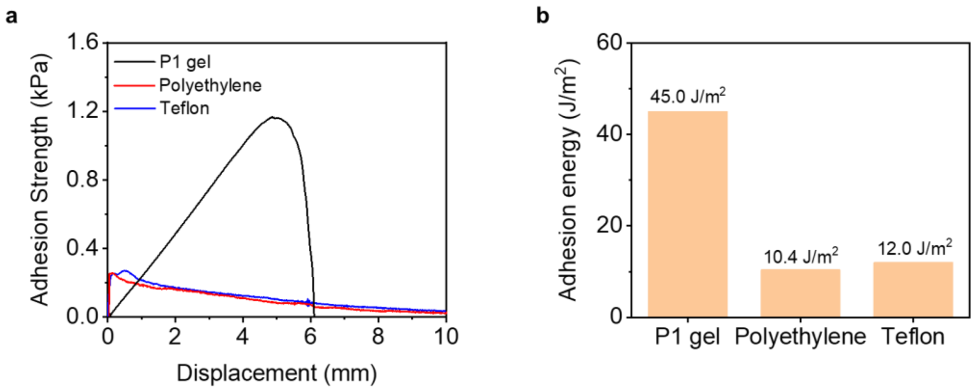

Supplementary Figure 62. (a) Adhesion strength curves and (b) adhesion energies of two P1 gels, two polyethylene substrates, and two Teflon substrates glued by P-Ru/P-S adhesives.

Because polyethylene and Teflon substrates are hydrophobic and water-insoluble, the aqueous solution of P-Ru/P-S cannot penetrate into them. The adhesion energy of P-Ru/P-S-glued P1 gels is more than 375% of that of P-Ru/P-S-glued polyethylene or Teflon substrates,

which revealed that the interpenetration of P-Ru/P-S with P1 enhanced the adhesion. This result is in line with the computer simulation results, which showed that interpenetration increased the interaction energy of P-Ru/P-S with P1 (Supplementary Table 2).

Adhesion strength of P-Ru/P-S-glued P1 gels in different aqueous environments

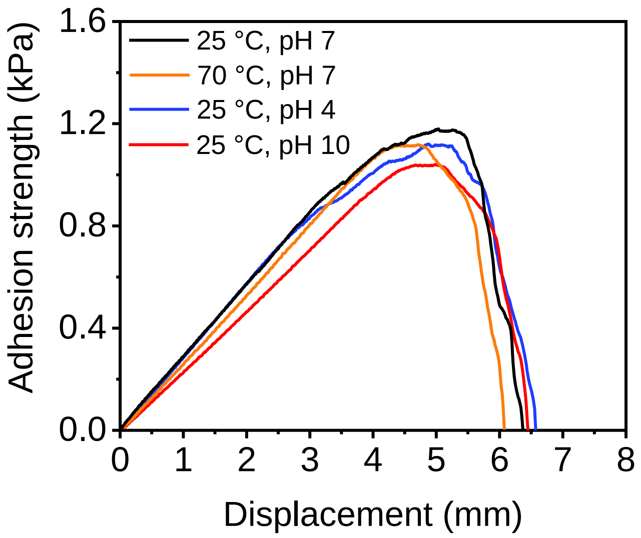

Supplementary Figure 63. The adhesion strengths of P1 gels glued with P-Ru/P-S after immersion in different aqueous environments for 24 h.

Volume changes of P2 and P3 gels under different stimuli

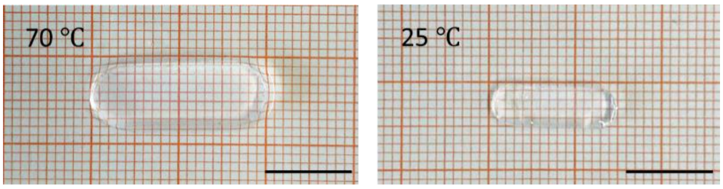

Supplementary Figure 64. Photos of P2 gel in water at 70 °C (left) and 25°C (right). First, a photo of the P2 gel at 70 °C was taken. The gel was then immersed in water at 25 °C for 1 h, and another photo was taken. Scale bars: 10 mm.

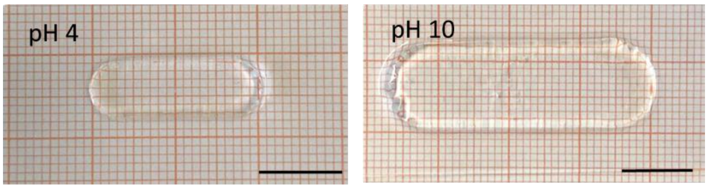

Supplementary Figure 65. Photos of P3 gel at pH 4 (left) and pH 10 (right). First, a photo of the P3 gel with pH = 4 was taken. The gel was then immersed in a solution with pH = 10 for 30 min, and another photo was taken. Scale bars: 10 mm.

Measurements of adhesion strength of P1/P2 and P1/P3 gel assemblies

A P2 gel was placed in water at 70 °C to reach swelling equilibrium before the adhesion measurements. Then, a P-Ru/P-S sol (6 wt%, 5 μL/cm^2) was spread on the surfaces of a P1 gel and a P2 gel. The length, width and thickness of each piece of gel were 3.0 cm, 1.5 cm and 0.2 mm, respectively. The P1 gel was pressed on top of the P2 gel (overlap area 2 cm^2). The gels were placed in a sealed container at 70 °C for 6 h. Then, the obtained gel assembly was immersed in water before further investigation. Lap shear adhesion tests for the glued P1/P2 gel assembly were performed using a tensile testing machine (Zwick/Roell) after immersing the glued P1/P2 gel assembly at 70 °C in water for 24 h (the initial gel assembly) and after the glued P1/P2 gel assembly was bent and unbent for 10 cycles in response to temperature changes.

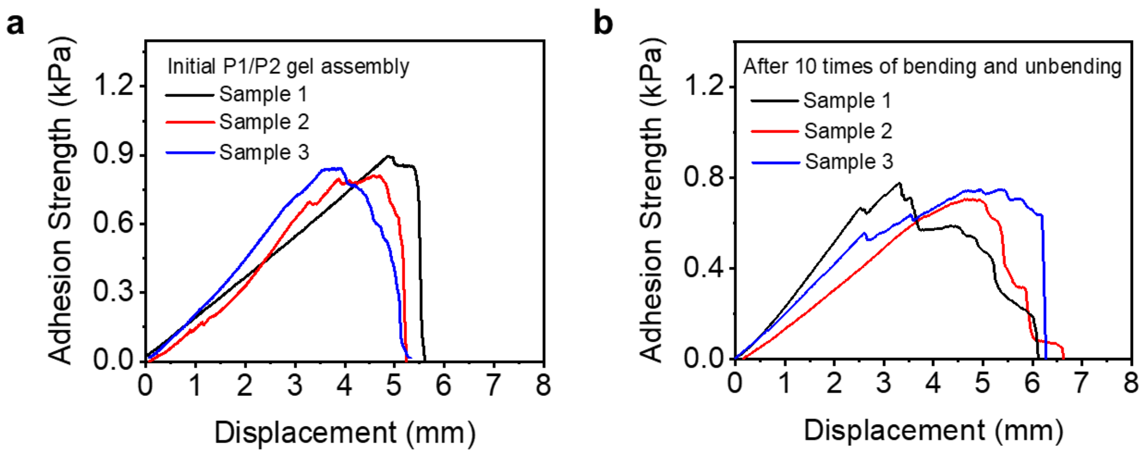

Supplementary Figure 66. The adhesion strengths of a P1/P2 gel assembly (a) before and (b) after actuation in response to temperature changes for 10 cycles. Three measurements were conducted under each condition, which showed that the adhesion measurements were reproducible.

A P3 gel was placed in water at pH 4 to reach swelling equilibrium before the adhesion measurements. Then, a P-Ru/P-S sol (6 wt%, 5 μL/cm^2) was spread on the surfaces of a P1 gel and P3 gel. The length, width and thickness of each piece of gel were 3.0 cm, 1.5 cm and 0.2 mm, respectively. After that, the P1 gel was pressed on top of the P3 gel (overlap area 2 cm^2). The gels were placed in a sealed container at 70 ℃ for 6 h. Then, the obtained hydrogel assembly was immersed in water at pH 4 before further investigation. Lap shear adhesion tests for the glued P1/P3 gel assembly were performed using a tensile testing machine (Zwick/Roell) after immersing the glued P1/P3 gel assembly in water at pH 4 for 24 h (the initial gel assembly) and after the glued P1/P3 gel assembly was bent and unbent for 10 cycles in response to pH changes.

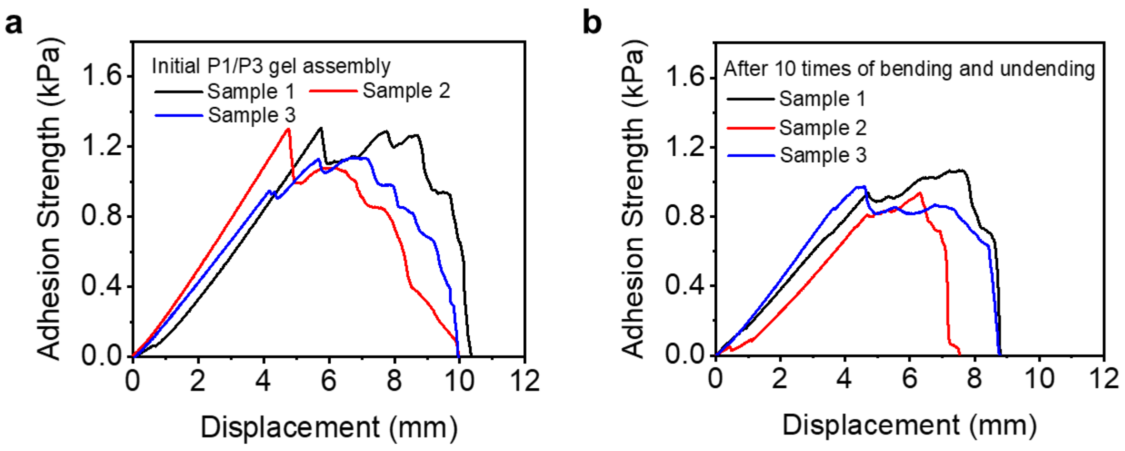

Supplementary Figure 67. The adhesion strengths of a P1/P3 gel assembly (a) before and (b) after actuation in response to pH changes for 10 cycles. Three measurements were conducted under each condition, which showed that the adhesion measurements were reproducible.

Shape changes of a P-Ru/P-S gel and swelling of a P-Ru/P-S gel in water

The mixture of P-Ru (1 wt%) and P-S (1 wt%) in water was heated at 70 °C for 6 h to form a P-Ru/P-S gel. The P-Ru/P-S gel was flexible and changed its shape upon pressing (Supplementary Fig. 68a). A P-Ru/P-S gel was placed in water for 24 h. The gel maintained its integrated network structure during swelling (Supplementary Fig. 68b).

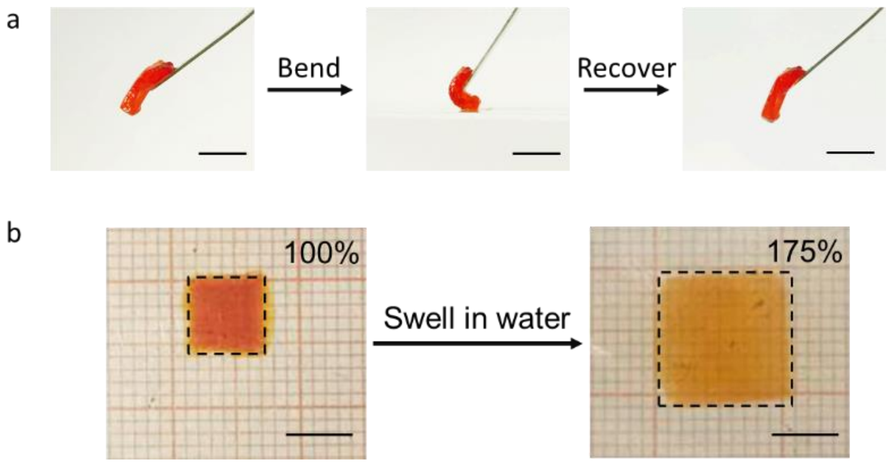

Supplementary Figure 68. (a) Bending and shape recovery of a P-Ru/P-S gel. (b) Swelling of a P-Ru/P-S gel in water. Scale bars: 5 mm.

Soft robot based on a responsive hydrogel assembly for maze navigation

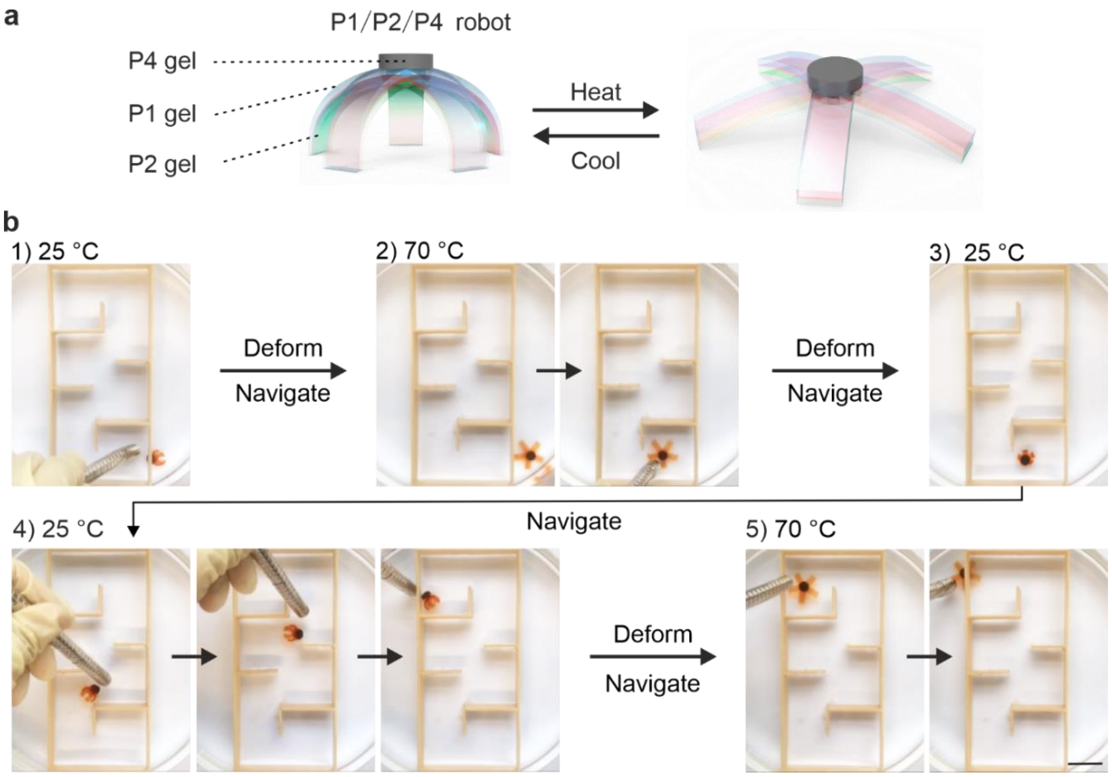

Supplementary Figure 69. Soft robot based on a thermoresponsive and magnetic-particle-containing hydrogel assembly for maze navigation. P1 is a nonresponsive gel, P2 is a thermoresponsive gel, and P4 is a magnetic-particle-containing gel. (a) Schematic of a P1/P2/P4 robot prepared by gluing the gel units using the P-Ru/P-S adhesive. (b) Photos of the P1/P2/P4 robot passing through a maze controlled by temperature and a magnetic field. Scale bar: 20 mm.

References

1. Koynov, K. & Butt, H.-J. Fluorescence correlation spectroscopy in colloid and interface science. *Current Opinion in Colloid & Interface Science* **17**, 377–387 (2012).

2. Raccis, R. et al. Probing mobility and structural inhomogeneities in grafted hydrogel films by fluorescence correlation spectroscopy. *Soft Matter* **7**, 7042-7053 (2011)

3. Rose, S. et al. Nanoparticle solutions as adhesives for gels and biological tissues. *Nature* 2014, **505**, 382-385 (2014).

4. Plimpton, S. Fast Parallel Algorithms for Short-Range Molecular Dynamics. *J. Comput. Phys.* **117**, 1-19 (1995).

5. Heinz, H., Vaia, R., Farmer, B. & Naik, R. Accurate simulation of surfaces and interfaces of face-centered cubic metals using 12− 6 and 9− 6 Lennard-Jones potentials. *J. Phys. Chem. C* **112**, 17281-17290 (2008).

6. Frisch, M.J et al. *Gaussian 16*. Gaussian, Inc. Wallingford, CT (2016).

7. Zhao, Y. & Truhlar, D.G. A new local density functional for main-group thermochemistry, transition metal bonding, thermochemical kinetics, and noncovalent interactions. *J. Chem. Phys.* **125**, 194101 (2006).

8. Chen, Q. & Lu, T. Forcefit code, http://www.keinsci.com/research/forcefit.

9. Brandt, P., Norrby, T., Åkermark, B. & Norrby, P.-O. Molecular Mechanics (MM3*) Parameters for Ruthenium(II)−Polypyridyl Complexes. *Inorg. Chem.* **37**, 4120-4127 (1998).

10. Rappe, A.K., Casewit, C.J., Colwell, K.S., Goddard, W.A. & Skiff, W.M. UFF, a full periodic table force field for molecular mechanics and molecular dynamics simulations. *J. Am. Chem. Soc*. **114**, 10024-10035 (1992).

11. Nose, S. Constant temperature molecular dynamics methods. *Prog. Theor. Phys. Supp.* **103**, 1-46 (1991).

12. Hockney, R.W. & Eastwood, J.W. Computer simulation using particles; CRC Press: New York, London, 1988.
